# Synthesis of 2-acetylnoviosamine derivatives by hydrogenolytic cleavage of a spirocyclopropane†

**DOI:** 10.1039/d5ob00469a

**Published:** 2025-04-17

**Authors:** Maruan D. Salim, Isabella Ferrara, Olivier Blacque, Karl Gademann

**Affiliations:** a Department of Chemistry, University of Zurich Winterthurerstrasse 190 8057 Zürich Switzerland karl.gademann@chem.uzh.ch

## Abstract

The preparation of a 2-acetamido derivative of the rare 5,5-*gem*-dimethyl-deoxy carbohydrate noviose is reported in this study. The synthesis starts from readily available *N*-acetyl-d-mannosamine and tackles the introduction of the *gem*-dimethyl structural feature *via* a cyclopropanation and hydrogenolytic cleavage strategy, which can enable the synthesis of 2-amino noviose derivatives of both l- and d-noviose.

## Abstract 2

The preparation of a 2-acetamido derivative of the rare 5,5-*gem*-dimethyl-deoxy carbohydrate noviose is reported in this study.

## Graphical abstract

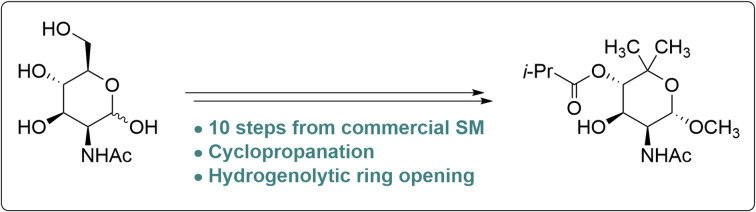

## Introduction

Noviose is a natural yet rare 5,5-*gem*-dimethyl-deoxyhexose which was initially discovered as a structural constituent of the aminocoumarin antibiotic novobiocin.^1–3^ Structural analogues have since been identified as carbohydrate moieties in other biologically active natural product antibiotics such as aminocoumarins^4^ (l-noviosyl analogues) and fidaxomicin^5^ (d-noviosyl analogue). The noviose-derived moiety plays a pivotal role in the binding of these glycosylated natural products to their respective targets.^6–8^ Structural analogues lacking this carbohydrate unit exhibit significantly decreased biological activity.^9,10^ Starting with the first total synthesis of l-noviose by Kiss and Spiegelberg in 1964,^11^ numerous other syntheses of d-^12–14^ and l-noviose^15–18^ derivatives, including stereoselective^19–23^ as well as enantiodivergent^24^ approaches, have been reported in the literature.

Modified carbohydrate fragments offer the advantage of altering the pharmacokinetic/pharmacodynamic (PK/PD) profile and can help gain insight into the structure–activity relationship (SAR) of biologically active molecules.^25,26^ Amino sugars are carbohydrates with at least one hydroxyl group being substituted with an amine. They are widespread in nature and contribute to the biological activity of many natural products.^27^ The basicity of nitrogen has for example been linked to an improvement in the active transport of macrolide antibiotics into cells.^28^ The most prevalent members are 2-amino sugars.^29^

In connection with our group's research on novel fidaxomicin-derived antibiotics,^30–32^ access to a 2-amino-d-noviose derivative could enable further glycodiversification of fidaxomicin derivatives. We envisage an altered PK/PD profile by merging the noviose moiety with the biologically relevant 2-amino sugar functionality.

**Fig. 1:**
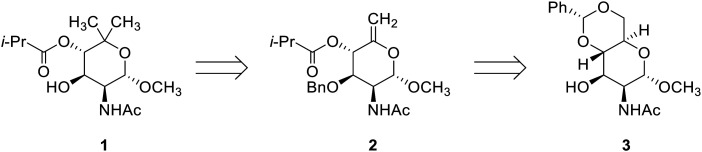
Synthetic plan towards 2-acetylnoviosamine 1.

Access to the *gem*-dimethyl structural feature in the 2-amino sugar 1 (Fig. 1) would be possible *via* a cyclopropanation and hydrogenolytic cleavage strategy based on the assumption that ring-opening preferentially occurs at the sterically most accessible site. The 5-*exo*-methylene-bearing precursor 2 can be traced back to the literature-known^33^ mannosamine derivative 3, which was accessed *via* an alternative route of two steps starting from *N*-acetyl-d-mannosamine. The mannose scaffold has previously been utilized by our group as a starting point towards other noviose derivatives.^32^ The use of orthogonal protecting groups which allow for site-selective functionalization of C(3)–O and C(4)–O would render the present approach a generally applicable route to synthesize 2-amino-noviose derivatives.

## Results and discussion

**Fig. 2:**
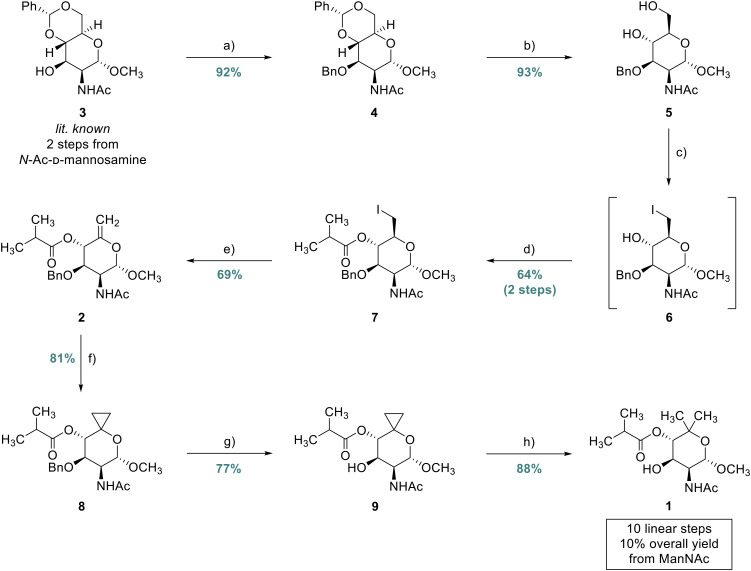
Synthesis of methyl 2-acetamido-2-deoxy-4-*O*-isobutyryl-4-*O*-demethyl-d-noviopyranoside (1): (a) BnBr (1.5 equiv.), BaO (3.0 equiv.), Ba(OH)_2_·8H_2_O (0.5 equiv.), DMF (0.2 M), 23 °C, 16 h, 92%; (b) AcOH/H_2_O (4 : 1 (v : v), 0.4 M), 70 °C, 4 h, 93%; (c) I_2_ (1.5 equiv.), PPh_3_ (1.5 equiv.), ImH (2.0 equiv.), THF (0.1 M), reflux, 3.5 h; (d) *i*-PrCOCl (1.5 equiv.), pyridine (2.0 equiv.), CH_2_Cl_2_ (0.1 M), 0 to 23 °C, 12 h, 64% (2 steps); (e) AgF (3.0 equiv.), pyridine (0.5 M), 23 °C, 24 h, 69%; (f) ZnEt_2_ (5.0 equiv.), CF_3_CO_2_H (5.0 equiv.), CH_2_I_2_ (6.0 equiv.), CH_2_Cl_2_ (35 mM), 0 to 23 °C, 36 h, 81%; (g) NBS (1.2 equiv.), CaCO_3_ (4.5 equiv.), *hv* (456 nm), CCl_4_/H_2_O (20 : 1, 0.25 M), 23 °C, 20 min, 77%; and (h) H_2_ (100 bar), PtO_2_ (2.0 equiv.), AcOH (0.18 mM), 23 °C, 14 d, 88% (qNMR purity: 84.2%, SD = 2.1%, *n* = 3, see the ESI† for details).

We started with the preparation of the literature-known compound 3 (previously prepared from different starting materials)^33^ in two steps from commercially available *N*-acetyl-d-mannosamine by Fischer glycosylation^34^ and benzylidenation.^35^ Benzylation at C(3)–O was then mediated by a mixture of benzyl bromide, barium oxide, and barium hydroxide octahydrate which delivered the fully protected carbohydrate 4 in 92% yield (Fig. 2).^35^ Subsequent hydrolysis of the benzylidene acetal in a mixture of acetic acid and water proceeded smoothly to provide the diol 5 in 93% yield.^36^ The sequence from methyl 2-acetamido-2-deoxy-d-mannopyranoside to 4,6-diol 5 was also carried out in one run with a single chromatographic purification to afford 9.4 g (3 steps, 61%) of the desired diol 5. With compound 5 in hand, regioselective iodination of the primary alcohol was achieved under Garegg–Samuelsson conditions^37^ to deliver primary iodide 6 which was acylated with isobutyryl chloride to afford the iodo-ester 7 (2 steps, 64%). Preliminary experiments to perform the elimination of the iodo group using sodium hydride resulted in poor yields of the *exo*-methylene-bearing pyranoside 2 (data not shown). The reaction yield and reproducibility were markedly improved by using silver(i) fluoride in pyridine to deliver olefin 2 in 69% yield (crystallographic data in the ESI, CCDC 2430253†).^38,39^ Formal hydromethylation of olefin 2 was attempted to provide a more step-efficient approach to synthesize the target compound but only trace amounts of the desired reaction product could be obtained (data not shown).^40^

**Fig. 3:**
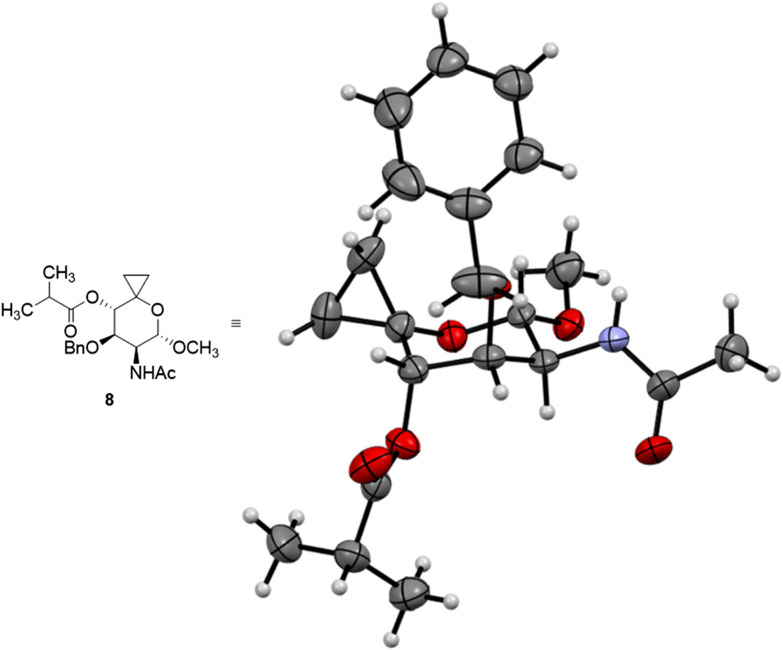
Oak Ridge thermal-ellipsoid plot of spirocyclopropane 8 obtained from single crystal X-ray diffraction analysis. The thermal ellipsoids are depicted at the 50% probability level. For crystallographic data see the ESI or CCDC 2430254.†

Next, a Simmons–Smith cyclopropanation was applied to convert olefin 2 into the respective spiro-[2,5]-system 8. After screening a variety of conditions (for selected conditions, see Table 1), Shi's carbenoid was found to efficiently provide the desired product 8 in 81% yield and its structure could also be confirmed by single-crystal X-ray diffraction analysis (Fig. 3).^41^ Simultaneous debenzylation at C(3)–O and hydrogenolytic cleavage of the cyclopropane in a single step from compound 8 would constitute a practical reaction sequence. Therefore, we attempted to directly subject the spiro compound 8 to Adams’ catalyst and molecular hydrogen. However, mainly dearomatization of the benzyl group was observed (based on UHPLC-MS/UV and NMR analysis of the crude reaction mixture, see Fig. S2–S4†). As a consequence, we tried to first deprotect the benzyl-protected C(3)–O group utilizing palladium on activated charcoal under elevated hydrogen pressure (50 bar), but the conversion of the substrate only proceeded slowly (see Fig. S5†). A light-mediated debenzylation process using *N*-bromosuccinimide was found to be an improved alternative, offering suitable functional group compatibility to provide the desired product 9 in 77% yield after 20 min of reaction time.^42^

**Table 1:** Zinc-carbenoids and selected conditions utilized in screening of the Simmons–Smith cyclopropanation reaction

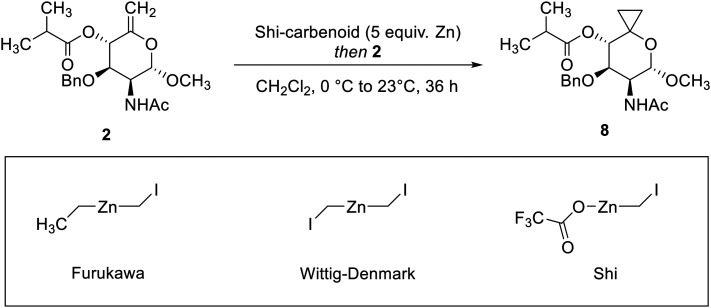			
Entry	Deviation from the standard protocol (see ESI† synthetic procedures)	Scale	Yield^a^
1	None	1021 mg	81%
2	None	50 mg	79%
3	Furukawa-carbenoid (formed *in situ*) instead of Shi-carbenoid (preformed), 23 °C to 40 °C	50 mg	12%
4	Wittig–Denmark-carbenoid (preformed) instead of Shi-carbenoid (preformed)	70 mg	26%
5	As entry 4, but Et_2_O/CH_2_Cl_2_ (3 : 1) instead of CH_2_Cl_2_	70 mg	46%

a Yields refer to isolated yield after chromatographic purification.

Hydrogenolytic cleavage of the cyclopropane moiety with Adams’ catalyst in acetic acid proceeded with high selectivity towards the presumably sterically more accessible site to provide the targeted 2-amino noviose derivative 1 in 88% yield with a purity of 84.2% (qNMR, SD = 2.1%, *n* = 3, see the ESI† for details) and could be further purified by preparative HPLC if required. In addition, we were able to establish in preliminary experiments that methyl pyranoside 1 could be hydrolyzed to its hemiacetal (see the ESI†).

In conclusion, the present approach constitutes the first synthesis of a d-noviosamine derivative. Furthermore, we describe access to the noviose C-skeleton by employing a hydrogenolytic cyclopropane opening. The outlined sequence provides the target compound 1 in ten linear steps starting from commercially available *N*-acetyl-d-mannosamine with an overall yield of 10%. The synthesis could represent a generally applicable method to synthesize 2-amino-noviose derivatives with various substitution patterns. A disadvantage of the route is the use of a photochemical method for effective debenzylation, which has only been demonstrated on a small scale (0.5 mmol). Difficulty in scale-up could potentially be remediated by conducting the respective step in a flow setup or utilizing the slow hydrogenolytic debenzylation with palladium on activated charcoal. The outlined sequence to access sugar 1 provides a valuable framework for the synthesis of 2-amino noviose derivatives of both l- and d-noviose. In addition, the free hemiacetal can be readily accessed from protected carbohydrate 1. These 2-amino noviose moieties have significant potential for use in the synthesis of glycoside antibiotics, offering a promising avenue to develop compounds with modified PK/PD profiles.

## Materials and methods

ESI tables, figures, detailed experimental procedures, and additional analytical data (*e.g.* NMR and MS) can be found in the ESI.† Additional references are also included in the ESI.†^43^

## Chemical synthesis and characterization of compounds

Unless indicated differently, all chemicals used were of reagent grade, purchased from commercial sources, and used as received. All solvents used in the reactions were obtained from commercial sources and used as received if not indicated differently. Reactions were carried out under an inert atmosphere (N_2_) using flame-dried glassware and anhydrous solvents, if not indicated otherwise. Detailed information on the reaction conditions, experimental procedures, instruments used, and the characterization of all newly synthesized compounds including 1- and 2-D NMR spectral data can be found in the ESI.†

## Author contributions

Maruan D. Salim: methodology, investigation, formal analysis, writing – original draft, and visualization. Isabella Ferrara: conceptualization, project administration, supervision, validation, and writing – review & editing. Olivier Blacque: investigation and formal analysis. Karl Gademann: conceptualization, funding acquisition, project administration, resources, supervision, validation, and writing – review & editing.

## Data availability

A preprint has been deposited on ChemRxiv at https://doi.org/10.26434/chemrxiv-2025-45f9c.

The data supporting this article have been included as part of the ESI.†

Selected raw data for this article, including NMR, IR, HRMS, and XRD data, are available in Zenodo at https://doi.org/10.5281/zenodo.15012672.

Crystallographic data for compounds 2 and 8 have been deposited at the CCDC: 2430253 (2) and 2430254 (8).†

## Conflicts of interest

The authors declare no competing interests.

## Supplementary Material

OB-023-D5OB00469A-s001

OB-023-D5OB00469A-s002
