# Supplementary material for: Synthesis of 2-acetylnoviosamine derivatives by hydrogenolytic cleavage of a spirocyclopropane
Source: Org Biomol Chem. 2025 Apr 17;23(20):4873–8. doi: 10.1039/d5ob00469a (PMC12035602; doi:10.1039/d5ob00469a)
Supplement: OB-023-D5OB00469A-s001 [file OB-023-D5OB00469A-s001.pdf]

Supplementary Information (SI) for Organic & Biomolecular Chemistry.
This journal is © The Royal Society of Chemistry 2025

Supporting Information

Synthesis of 2-Acetylnoviosamine Derivatives by Hydrogenolytic Cleavage of a Spirocyclopropane

Maruan D. Salim[a], Isabella Ferrara[a], Olivier Blacque[a], and Karl Gademann*[a]

[a]Department of Chemistry, University of Zurich, Winterthurerstrasse 190, 8057 Zürich, Switzerland.

*Correspondence to Karl Gademann. E-Mail: karl.gademann@chem.uzh.ch

Contents

I. Overlay ^{1}H-NMR spectra of 2-acetylnoviosamine 1, directly after the reaction (top) and after HPLC purification (bottom) 3

II. Undesired dearomatization of compound 8 4

III. Slow debenzylation of compound 8 using Pd/C 6

IV. Preliminary results for hydrolysis of methyl pyranoside 1 6

V. General information 9

V.I. Synthesis and purification 9

V.II. Devices, methods, and related information 9

VI. Synthetic procedures 13

VI.I. Methyl (10*R*)-2-acetamido-4,6-*O*-benzylidene-2-deoxy-α-D-mannopyranoside (3) 13

VI.II. Methyl 2-acetamido-3-*O*-benzyl-2-deoxy-α-D-mannopyranoside (5) 15

VI.III. Methyl 2-acetamido-2-deoxy-3-*O*-benzyl-4-*O*-isobutyryl-6-iodo-6-deoxy-α-D-mannopyranoside (7) 17

VI.IV. (3*S*,4*R*,5*S*,6*S*)-5-Acetamido-4-(benzyloxy)-6-methoxy-2-methylenetetrahydro-2*H*-pyran-3-yl isobutyrate (2) *Exp: MDS-114* 19

VI.V. (5*S*,6*S*,7*R*,8*S*)-6-Acetamido-7-(benzyloxy)-5-methoxy-4-oxaspiro[2.5]octan-8-yl isobutyrate (8) *Exp: MDS-125* 20

VI.VI. (5*S*,6*S*,7*R*,8*S*)-6-Acetamido-7-hydroxy-5-methoxy-4-oxaspiro[2.5]octan-8-yl isobutyrate (9) *Exp: MDS-124* 21

VI.VII. Methyl 2-acetamido-2-deoxy-4-*O*-isobutyryl-4-*O*-demethyl-α-D-noviopyranoside (1) *Exp: MDS-107* 22

VII. Purity determination of crude 2-acetylnoviosamine 1 by qNMR 23

VIII. NMR spectra 26

IX. Crystallographic data XRD 53

X. References SI 54

I. Overlay ^{1}H-NMR spectra of 2-acetylnoviosamine 1, directly after the reaction (top) and after HPLC purification (bottom)

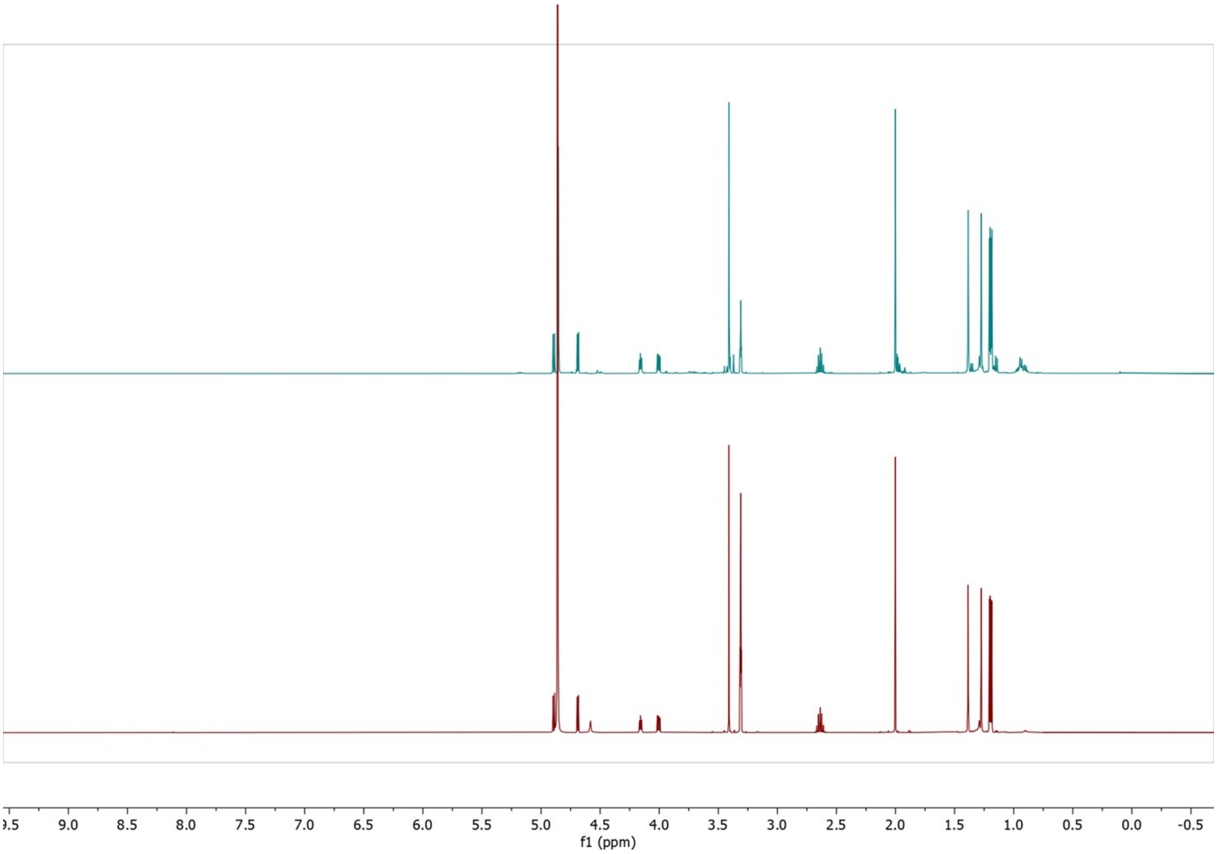

*Figure S1: Overlay of ^{1}H-NMR spectra (500 MHz, CD_3OD) of target compound **1**, directly after the reaction (top) and purified by preparative HPL chromatography (bottom).*

II. Undesired dearomatization of compound 8

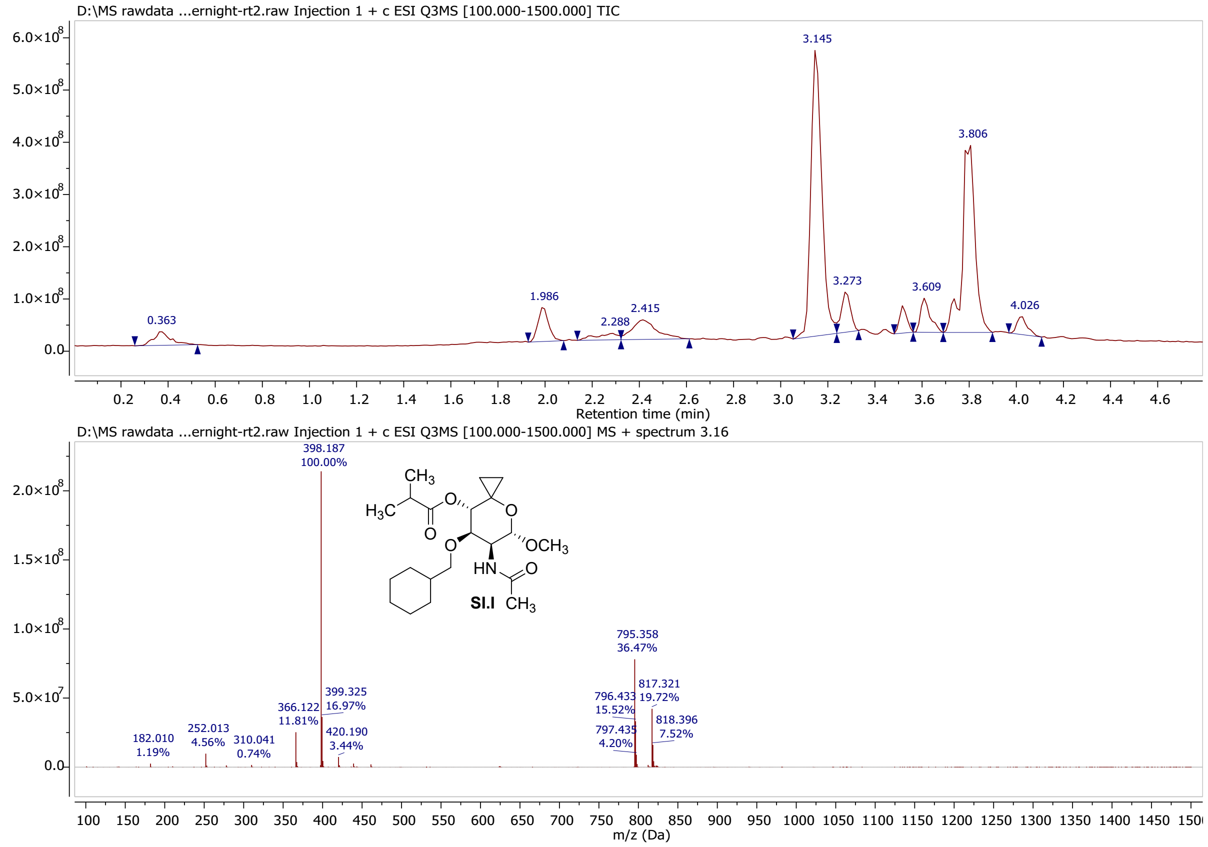

*Figure S2: Top: UHPLC-DAD-ESI-MS mass trace of the reaction mixture (MDS-083) obtained by subjecting compound **8** to PtO_2 (1.0 equiv.), H_2 (20 bar) in AcOH (8.5 mM) for 18 h at 23 °C. The peak at 3.806 min was not assigned, see figure S3 for blank. Bottom: Mass spectrum (pos. mode) of highest intensity peak and suggested structure of the main reaction product (**SI.I**, calc. 397.246 m/z).*

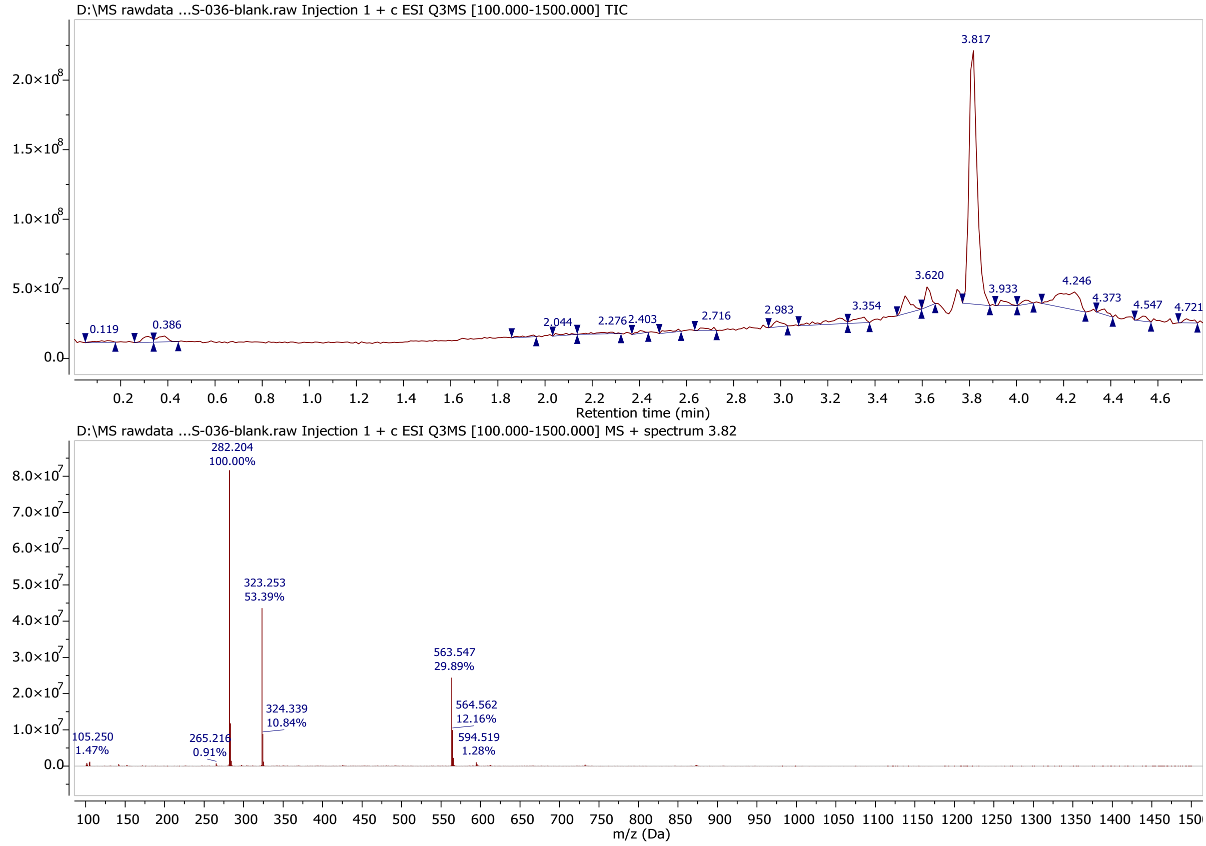

Figure S3: Blank (MeOH). Top: UHPLC-DAD-ESI-MS mass trace. Bottom: Mass spectrum (pos. mode)

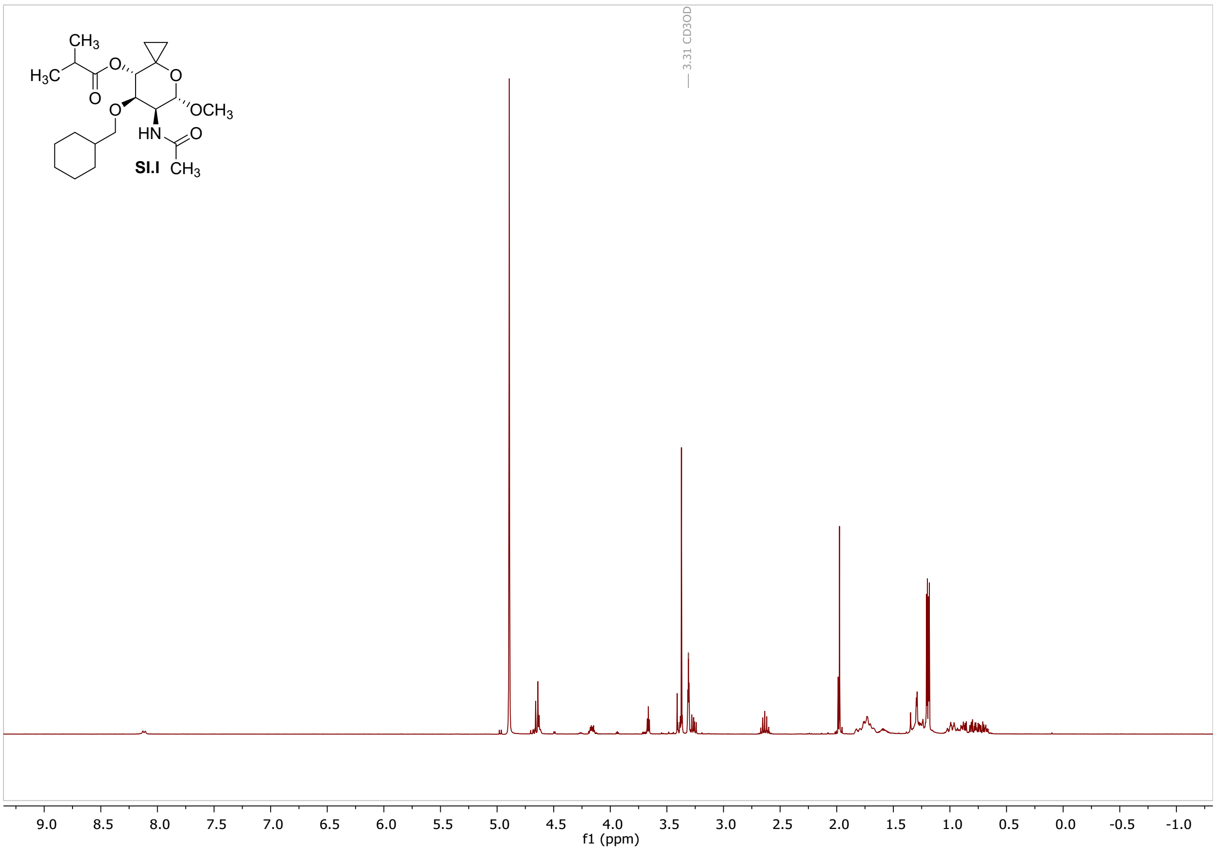

*Figure S4: ^{1}H-NMR analysis of the crude reaction mixture (MDS-083) obtained by subjecting compound **8** to PtO_2 (1.0 equiv.), H_2 (20 bar) in AcOH (8.5 mM) for 18 h at 23 °C (AcOH neutralized with NaHCO3 and extracted with CH_2Cl_2). Suggested structure of the main constituent of the mixture **SI.I**.*

III. Slow debenzylation of compound 8 using Pd/C

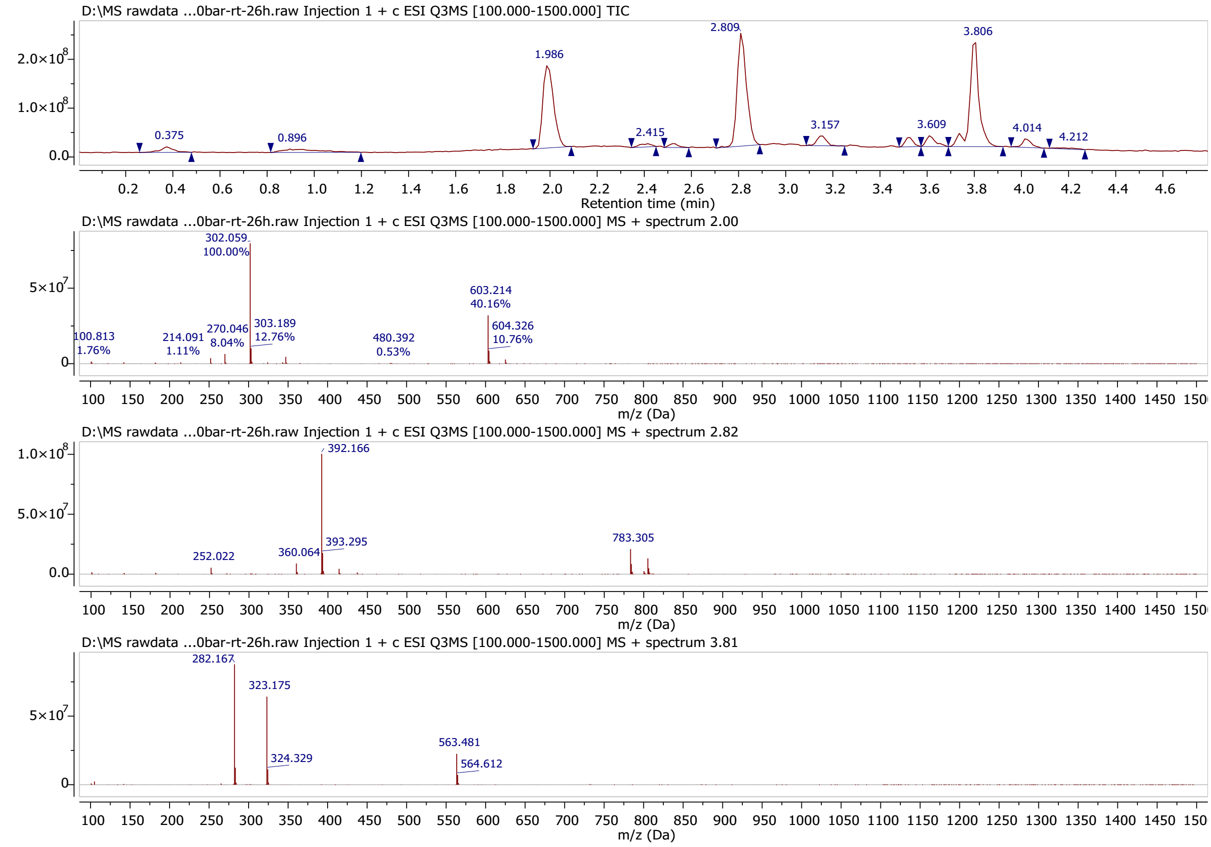

*Figure S5: Top: UHPLC-DAD-ESI-MS mass trace of reaction mixture of slow debenzylation of Compound **8** after 26 h (Fastest condition tested shown: H_2 (50 bar), Pd/C (10 mol%), EtOAc (8.5 mM)). Upper middle: Mass spectrum (pos. mode) of desired product **9**. Lower middle: Mass spectrum (pos. mode) of reaction substrate **8**. Bottom: Mass spectrum (pos. mode) at 3.81 min not assigned, see figure S3 for blank.*

IV. Preliminary results for hydrolysis of methyl pyranoside 1

There is indication that hydrolysis of methyl pyranoside **1** to the corresponding hemiacetal **SI.II** can be achieved with triphenylcarbonium tetrafluoroborate according to the procedure of Kumar and coworkers.[1]

Compound **1** (2.0 mg, 6.6 µmol, 1.0 equiv.) and triphenylcarbonium tetrafluoroborate (2.2 mg, 6.6 µmol, 1.0 equiv.) were dissolved in anhydrous CH_2Cl_2 (1.0 mL, 6.6 mM) and stirred at 23 °C for 24 h before 0.5 mL aq. $NaHCO_3$ soln. (sat., 0.5 mL) was added. Solvents were removed *in vacuo* to give a colorless solid which was purified by column chromatography (gradient CH_3OH/CH_2Cl_2, 1:20 to 1:7) to give a product enriched fraction (1.2 mg)

HR-ESI-MS (+) (CH_3OH) calculated for $C_{13}H_{23}O_6NNa^+$ [*M*+Na]$^+$: 312.14176, found: 312.14133.

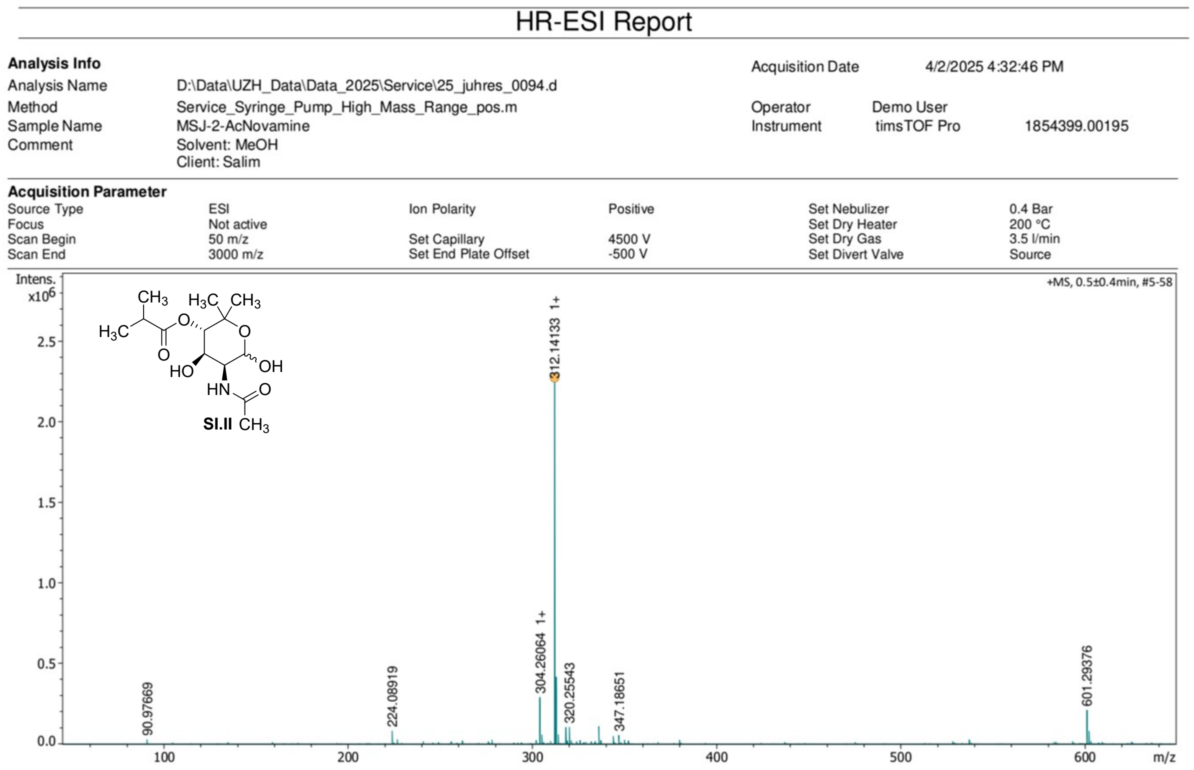

*Figure S6: HR-ESI-MS mass trace for product enriched fraction of hemiacetal **SI.II.***

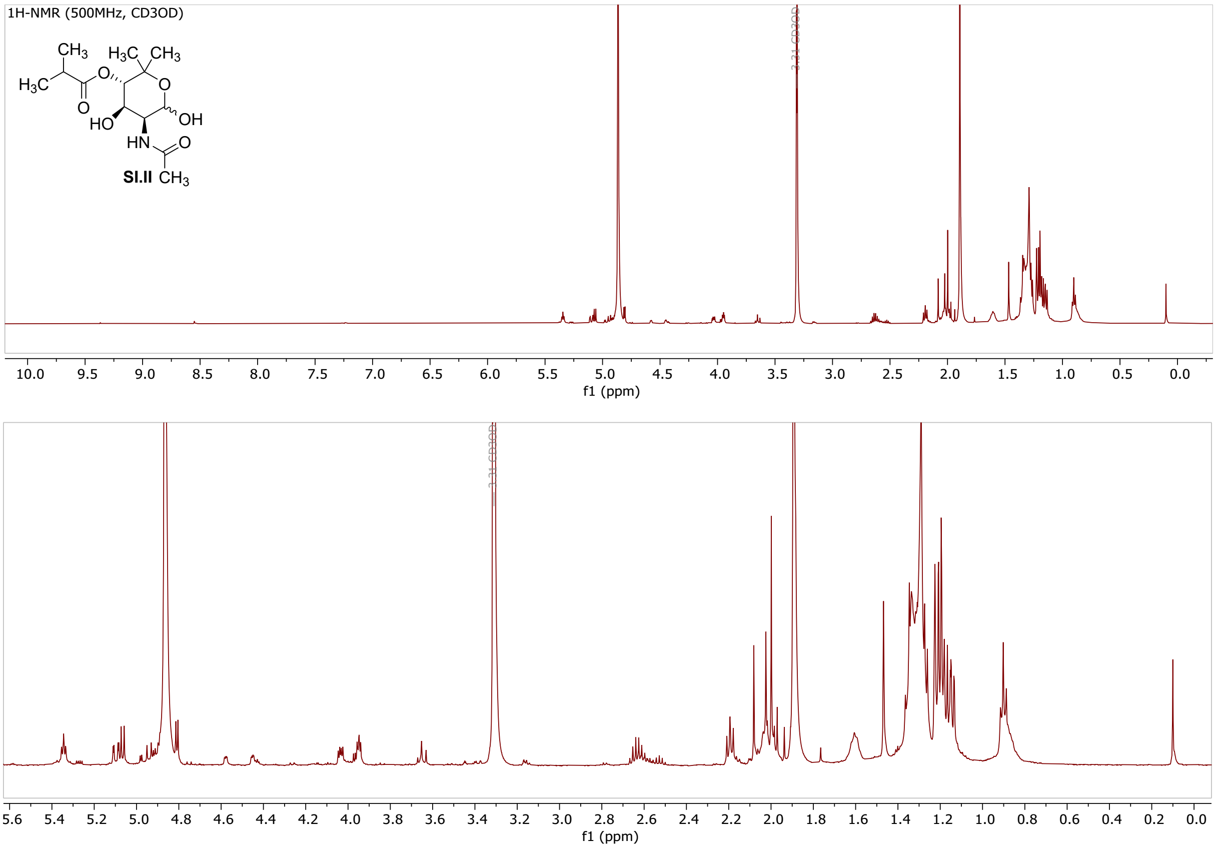

*Figure S7: ^{1}H-NMR spectrum of a chromatographic fraction enriched with compound **SI.II**. Signal at 1.90 ppm presumably contamination with AcOH.*

V. General information

V.I. Synthesis and purification

Unless otherwise stated, all chemicals were of reagent grade, purchased from commercial sources (*Abcr*, *Acros organics*, *Alfa Aesar*, *Apollo Scientific*, *Combi-Blocks*, *Fluka*, *Fluorochem*, *Merck*, *Sigma-Aldrich*, *TCI*, *Thermo Fisher Scientific*, *VWR*) and used without further purification. Solvents for reactions were anhydrous unless otherwise stated and of p. a. grade or distilled prior to their use; THF, Et_2O, CH_2Cl_2, toluene and CH_3CN were dried and supplied by a *mBraun MB-SPS* solvent purification system, using the columns *MB-KOL- MT2-160°* (THF), *MB-KOL-A* (2×, CH_2Cl_2), *MB-KOL-A* and *MB-KOL-C* (CH_3CN, toluene), *MB-KOL-A* and *MB-KOL-MT2* (Et_2O). Volatile amines such as NEt_3 and DIPEA were freshly distilled. The synthesized compounds were stored under N_2 or Ar atmosphere at −20 °C. Reactions were stirred magnetically and carried out on a Schlenk line under N_2 with standard Schlenk/syringe techniques in oven-dried (160 °C) glass equipment which was additionally flame dried with a blow torch under HV. HV was equipped to the Schlenk manifold by an *Edwards RV 12* rotary vane vacuum pump equipped with a liquid N_2 cooled cold trap which achieved effective line pressures around $5 \cdot 10^{-3}$ mbar. When solvent ratios are mentioned they always refer to *volume:volume.* Reaction monitoring was performed by TLC and UPLC-DAD-ESI-MS. For TLC, silica coated aluminium plates (silica gel 60 F_{254}, *Merck*) were used. Spots on TLC were visualized using UV light (λ = 254 nm), iodine chamber or staining solutions of ethanolic sulfuric acid, cerium ammonium molybdate, *p*-anisaldehyde, and potassium permanganate (treated TLC-plates were gently heated with a heat gun when necessary). Evaporation of the solvents *in vacuo* was done with a rotary evaporator, and solvent traces were removed under HV. Reactions above 23 °C were heated in an oil bath, temperatures given in the procedures were measured in the oil bath. Column chromatography was conducted using silica (high-purity grade (w/ Ca, 0.1%), pore size 60 Å, 230-400 mesh, *Merck*) as stationary phase. Samples for purification by column chromatography were loaded onto the column dissolved in a minimal amount of the eluent or CH_2Cl_2 if not indicated differently.

V.II. Devices, methods, and related information

FT-IR

FT-IR spectra were acquired using a *SpectrumTwo* FT-IR Spectrometer (*Perkin–Elmer*) equipped with a *Specac Golden Gate*® ATR accessory. Samples were applied neat or as thin films by evaporation from the solvent indicated in parentheses. The absorption bands listed are designated with the letters s (strong), m (medium), and w (weak).

HPLC

Analytical and preparative HPLC was conducted on a *Prominence* modular HPLC instrument (*Shimadzu*) equipped with a *SPD-20A* UV/Vis detector (*Shimadzu*) using *Gemini NX* C18 RP columns (*Phenomenex*). The column used for analytical runs has internal dimensions of 150 mm × 4.6 mm, 3 µm particle size, and 110 Å pore size. The column used for preparative

runs has internal dimensions of 250 mm × 21.2 mm, 5 µm particle size, and 110 Å pore size. The LC was equipped with a *CBM-20A* system controller, *LC-20A* solvent delivery unit, *DGU-20A* degassing unit, *FRC-10A* fraction collector (all *Shimadzu*). The following solvents were used: H_2O + 0.1% HCOOH (A), CH_3CN + 0.1% HCOOH (B).

HR-ESI-MS:

Mass spectra were acquired using a *QExactive* (*Thermo Fisher Scientific, Bremen, Germany*) mass spectrometer equipped with a heated ESI source connected to a *Dionex Ultimate 3000* UPLC system. Samples were dissolved in CH_3OH, CH_3OH/CH_2Cl_2 3:1, CH_3OH/H_2O 1:1, DMSO/H_2O 1:10, or H_2O at ca. 50 µg mL^{-1} before injection of 1 µL on-flow with an *XRS* auto- sampler (*CTC, Zwingen, Switzerland*)(mobile phase: CH_3OH + 0.1% HCOOH or CH_3CN/H_2O (2:8) + 0.1% HCOOH; flow rate 120 µL mL^{-1}; ion source parameters: spray voltage 3.0 kV, capillary temperature 280 °C, sheath gas 30 L min^{-1}, s-lens RF level 55.0; aux gas temperature 250 °C; full scan MS in alternating (+)/(−)-ESI mode; mass ranges 80–1200, 133–2000, or 200– 3’000 amu; resolution (full width half-maximum) 70000; automatic gain control (AGC) target 3.00×10^6; maximum allowed ion transfer time (IT) 30 ms; mass calibration < 2 ppm accuracy for m/z 130.06619–1621.96509 in (+)-ESI with *Pierce*® ESI calibration solutions (*Thermo Fisher Scientific, Rockford, USA*); lock masses: ubiquitous erucamide (m/z 338.34174, (+)-ESI)).

Melting Point

Device: *B-545* melting point apparatus (*Büchi*). The samples were measured in soda glass melting point tubes *l* = 80 mm utilizing a temperature gradient of 2 °C/min

Nuclear Magnetic Resonance (NMR)

The spectra were acquired in deuterated solvent (supplied by *Deutero* and *Eurisotop*) at 300 K on a *Bruker AV2-400* (400 MHz) or *Bruker AV2-500* (500 MHz) device. Chemical shift (*δ*) was given in ppm relative to residual solvent resonances. The used solvent is indicated prior to the shifts in parentheses respectively. The chemical shift of residual solvent was gathered from literature.
Diastereotopic proton resonances are indicated using indices a and b (H_a and H_b) or for rigid ring systems such as trans-decalin-type ring fusion indices ax and eq (H_{ax} and H_{eq}) respectively, where ax is used for the (pseuo-)axial proton and eq for the (pseudo-)equatorial proton. Chemically equivalent carbons (including those related by fast molecular motion compared to the NMR timescale) are assigned with the same number, and the prime symbol (‘) is used to distinguish them. Visualization and processing of the acquired data was performed with the program *MestReNova* (version 12.0.0-20080).

Single Crystal X-Ray Diffraction (SC-XRD)

Single crystal X-ray diffraction data were collected at 160.0(1) K on a *Rigaku OD*

Supernova/Atlas diffractometer for compounds **2** and **8** using the Cu Kα radiation (λ = 1.54184 Å) from a dual wavelength X-ray source and an Oxford Instruments Cryojet XL cooler. For each analysis the selected suitable single crystal was mounted using polybutene oil on a flexible loop fixed on a goniometer head and immediately transferred to the diffractometer. Pre-experiment, data collection, data reduction and analytical absorption correction[2] were performed with the program suite *CrysAlisPro* (version 1.171.43.143a, *Rigaku Oxford Diffraction Ltd*, Yarnton, Oxfordshire, England). Using *Olex2*,[3] the structure was solved with the *SHELXT*[4] small molecule structure solution program and refined with the *SHELXL* program package[5] by full-matrix least-squares minimization on F^2. *PLATON*[6] was used to check the result of the X-ray analysis.

In the crystal structure of **2**, two crystallographically independent molecules are present in the asymmetric unit. During the refinement, the CO^{i}Pr group of one molecule was found to be disordered over two orientations (named A and B) with the site occupancy factors refined as 0.725(5) and 0.275(5), respectively. Restraints were applied in *SHELXL* to the bond distances and angles in the disordered parts using the *DANG*, *DFIX* and *SADI* instructions, and to the displacement parameters of the disordered atoms using the *SIMU* instruction. The H-atoms on the N-atoms were located in a difference Fourier map, and were freely refined with $U_{iso}(H) = 1.2U_{eq}(N)$. All remaining H-atoms were placed geometrically and refined isotropically using a riding model, with C—H = 0.95 Å (C-aromatic), 0.98 Å (C-methyl), 0.99 Å (C-methylene) and 1.00 Å (C-methine), in association with $U_{iso}(H) = 1.2U_{eq}(C)$ or $1.5U_{eq}$(C-methyl).

In the crystal structure of **8**, three crystallographically independent molecules are present in the asymmetric unit. During the refinement, the ^{i}Pr group of one molecule was found to be disordered over two orientations with the site occupancy factors refined as 0.527(6) and 0.473(6), respectively. The O-atom of a C=O group of a second molecule was also disordered over two positions with site-occupancy factors of 0.746(9) and 0.254(9). Similarity restraints were applied in *SHELXL* to the bond distances in the disordered parts using the *SADI* instruction, and to the displacement parameters of the disordered atoms using the *SIMU* instruction. The H-atoms on the N-atoms were located in a difference Fourier map, and were freely refined (positions and isotropic displacement parameters). All remaining H-atoms were placed geometrically and refined isotropically using a riding model, with C—H = 0.95 Å (C-aromatic), 0.98 Å (C-methyl), 0.99 Å (C-methylene) and 1.00 Å (C-methine), in association with $U_{iso}(H) = 1.2U_{eq}(C)$ or $1.5U_{eq}$(C-methyl).

CCDC- 2430253 (**2**) and CCDC- 2430254 (**8**) contain the supplementary crystallographic data for this paper. The data can be obtained free of charge from The Cambridge Crystallographic Data Centre via www.ccdc.cam.ac.uk/structures.

Specific Optical Rotary Power

Specific optical rotary powers were measured using a *Jasco P-2000* polarimeter in the solvents indicated in brackets (c is given in g/100 mL)

UPLC-DAD-ESI-MS

The utilized setup consists of a *Ultimate 3000 LC* instrument (*Thermo Fisher Scientific*) coupled to a triple quadrupole *Quantum Ultra EMR* MS (*Thermo Fisher Scientific*) using a reversed-phase column (*Kinetex® EVO* C18; 1.7 µm; 100 Å, 50 × 2.1 mm; *Phenomenex*). The UPLC was equipped with a *HPG-3400RS* pump, a *WPS-3000TRS* autosampler, a *TCC-3000RS* column oven and a *Vanquish* DAD detector (all *Thermo Fisher Scientific*). The following solvents were applied: H_2O + 0.1% HCOOH (A), CH_3CN + 0.1% HCOOH (B). The applied method had a constant flowrate of 0.4 mL min^{-1} and used a linear gradient: 0 min, 5% (B); 0.5 min, 5% (B); 3.5 min, 95% (B); 3.55 min, 100% (B); 4.8 min, 100% (B). Samples were prepared using HPLC grade solvents (CH_3CN, CH_3OH, H_2O) and filtered using a 4 mm syringe filter, PTFE (hydrophilic), pore size: 0.22 µm obtained from *BGB Analytik AG*. The MS was equipped with a *H-ESI II* ion source (*Thermo Fisher Scientific*). The source temperature was 250 °C, the capillary temperature 270 °C and capillary voltage 3500 V. Datasets were acquired at resolution 0.7 on Q3 in centroid mode.

VI. Synthetic procedures

VI.I. Methyl (10*R*)-2-acetamido-4,6-*O*-benzylidene-2-deoxy-α-D-mannopyranoside (3)

<u>Methyl 2-acetamido-2-deoxy-α-D-mannopyranoside (**SI.IIIα**) and methyl 2-acetamido- 2-deoxy-β-D-mannopyranoside (**SI.IIIβ**) *Exp: MDS-083*</u>

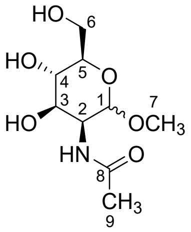

The compound was previously described in literature and the synthetic procedure was adapted from Petrović et al.[7] and implemented with minor modifications.

N-acetyl-D-mannosamine (97%, 34.5 g, 0.151 mol, 1.00 equiv.) was dissolved in CH_3OH (0.3 L, 0.5 M). Dowex 50W X8 (H^+-form, 17.3 g, 50.0 wt.%) was added in one portion and the reaction mixture was heated to reflux (85 °C) for 16 h. The reaction mixture was then allowed to cool to 23 °C before it was vacuum filtered. The filter cake was washed with CH_3OH (3 × 50 mL) before the filtrate was dried in vacuo to yield a pale yellow solid. Purification was achieved by column chromatography (CH_3OH/CH_2Cl_2, 14:86). The combined product containing fractions were evaporated in vacuo to afford the desired product as an amorphous colorless solid (68%, 24.2 g, 0.151 mol) (mixture of both anomeric epimers, α/β ≈ 8:1, ratio estimated by ^{1}H-NMR analysis from combined prod. containing fractions (figure S10, MDS-002). Anomer assignment based on NOESY (figure S15, MDS-002) and supported by XRD measurements at later stages).

$\boldsymbol{R}_\mathbf{F}$ (CH_3OH/CH_2Cl_2, 14:86) = 0.19.

HR-ESI-MS (+) (MDS-002, CH_3OH) calculated for $C_9H_{17}O_6NNa^+$ $[M+Na]^+$: 258.09481, found: 258.09448.

FT-IR (MDS-005, neat; α/β, 10:1) $\tilde{\nu}$ 3300m, 2932w, 2838w, 1644m, 1543m, 1442m, 1376m, 1292m, 1200w, 1130s, 1027s, 962s, 901w, 833w, 803m, 660m, 578m, 496s.

Methyl 2-acetamido-2-deoxy-α-D-mannopyranoside (SI.IIα, S18, minor anomer enriched fraction):

^{1}H-NMR (MDS-002, 500 MHz, CD_3OD) *δ* 4.57 (d, *J* = 1.4 Hz, 1H, C1<u>H</u>), 4.28 (dd, *J* = 4.9, 1.5 Hz, 1H, C2<u>H</u>), 3.89 (dd, *J* = 9.5, 4.8 Hz, 1H, C3<u>H</u>), 3.81 (d, *J* = 3.5 Hz, 2H, C6<u>H$_a$</u> and C6<u>H$_b$</u>), 3.57 (t, *J* = 9.7 Hz, 1H, C4<u>H</u>), 3.52–3.48 (m, 1H, C5<u>H</u>), 3.36 (s, 3H, C7<u>H$_3$</u>), 2.00 (s, 3H, C9<u>H$_3$</u>).

^{13}C{^{1}H}-NMR (MDS-002, 126 MHz, CD_3OD) *δ* 173.98 (1C, C8), 101.71 (1C, C1), 74.00 (1C, C5), 70.75 (1C, C3), 68.31 (1C, C4), 62.24 (1C, C6), 55.24 (1C, C7), 54.29 (1C, C2), 22.59 (1C, C9).

Methyl 2-acetamido-2-deoxy-β-D-mannopyranoside (SI.IIβ, S18, minor anomer enriched fraction):

^{1}H-NMR (MDS-002, 500 MHz, CD_3OD) δ 4.53 (d, *J* = 1.5 Hz, 1H, C1<u>H</u>), 4.44 (dd, *J* = 4.3, 1.5 Hz, 1H, C2<u>H</u>), 3.85 (d, *J* = 3.3 Hz, 2H C6<u>H$_a$</u> and C6<u>H$_b$</u>), 3.63 (dd, *J* = 9.6, 4.3 Hz, 1H, C3<u>H</u>),

3.52–3.48 (m, 1H, C4H), 3.47 (s, 3H, C7H_3), 3.23 (dt, *J* = 9.7, 3.3 Hz, 1H, C5H), 2.01 (s, 3H, C9H_3).

$^{13}C\{^1H\}$-NMR (MDS-002, 126 MHz, CD_3OD) δ 174.66 (1C, C8), 101.93 (1C, C1), 78.26 (1C, C5), 74.44 (1C, C3), 68.19 (1C, C4), 61.91 (1C, C6), 56.95 (1C, C7), 54.72 (1C, C2), 22.66 (1C, C9).

Methyl (10*R*)-2-acetamido-4,6-*O*-benzylidene-2-deoxy-α-D-mannopyranoside (**3**) *Exp: MDS-051*

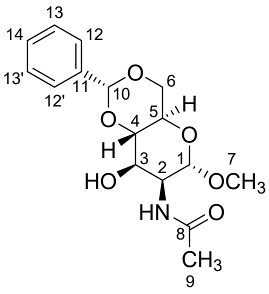

The compound was previously described in the literature[8]. In the present case the synthetic procedure for benzylidene acetal formation was adapted from Basu et al.[9] and implemented with minor modifications.

Methyl 2-acetamido-2-deoxy-D-mannopyranoside (**SI.II** ,α/β ≈ 8:1, 11.2 g, 47.5 mmol, 1.00 equiv.) was dissolved in CH_3CN (0.25 L, 0.19 M). Then benzaldehyde dimethyl acetal (10.7 mL, 71.3 mmol, 1.50 equiv.) and iron(III) chloride (1.5 g, 9.5 mmol, 0.20 equiv.) were added to the solution. The reaction was stirred for 14 h at 23 °C. The solvent was evaporated *in vacuo* and the resulting residue was taken up in EtOAc (0.25 L). The organic layer was washed with aq. $NaHCO_3$ soln. (5%, 3 × 50 mL), dried over anhydrous $MgSO_4$, filtered, and the solvent evaporated *in vacuo* to obtain a colorless solid, which was used in the next step without further purification. For analytical purposes the reaction was also carried out on smaller scale (*Exp: MDS-023*): starting from methyl 2-acetamido-2-deoxy-D-mannopyranoside (**SI.II**, α/β = 8.3:1, 201 mg, 0.854 mmol), and the product was purified by column chromatography (CH_3OH/CH_2Cl_2, 1:30) to obtain the desired benzylidene acetal **3** as colorless amorphous solid (73%, 202 mg, 0.625 mmol).

R_F (CH_3OH/CH_2Cl_2, 14:86) = 0.65.

HR-ESI-MS (+) (MDS-023, CH_3OH) calculated for $C_{16}H_{21}O_6NNa^+$ [*M*+Na]$^+$: 346.12611, found: 346.12618.

^1H-NMR (previously reported in $CDCl_3$[8], MDS-023, 500 MHz, CD_3OD) *δ* 7.53–7.47 (m, 2H, C12H and C12’H), 7.39–7.30 (m, 3H, C13H, C13’H, and C14H), 5.62 (s, 1H, C10H), 4.61 (d, *J* = 1.2 Hz, 1H, C1H), 4.38 (dd, *J* = 5.0, 1.4 Hz, 1H, C2H), 4.21 (dd, *J* = 10.1, 4.7 Hz, 1H, C6H_{eq}), 4.14 (dd, *J* = 10.2, 5.0 Hz, 1H, C3H), 3.88 (t, *J* = 10.1, 9.6 Hz, 1H, C4H), 3.85 (t, *J* = 10.2 Hz, 1H, C6H_{ax}), 3.74 (td, *J* = 9.9, 4.7 Hz, 1H, C5H), 3.38 (s, 3H, C7H_3), 2.04 (s, 3H, C9H_3).

$^{13}C\{^1H\}$-NMR (previously reported in $CDCl_3$[8],MDS-023, 126 MHz, CD_3OD) *δ* 174.10 (1C, C8), 139.18 (1C, C11), 129.93 (1C, C14), 129.05 (2C, C13 and C13’), 127.48 (2C, C12 and C12’), 103.37 (1C, C10), 102.58 (1C, C1), 80.15 (1C, C4), 69.74 (1C, C6), 67.45 (1C, C3), 64.76 (1C, C5), 55.36 (1C, C7), 54.84 (1C, C2), 22.55 (1C, C9).

VI.II. Methyl 2-acetamido-3-*O*-benzyl-2-deoxy-α-D-mannopyranoside (5)

The title compound was synthesized in two consecutive steps starting from methyl (10*R*)-2-acetamido-4,6-O-benzylidene-2-deoxy-α-D-mannopyranoside (**3**).

Methyl (10*R*)-2-acetamido-3-*O*-benzyl-4,6-*O*-benzylidene-2-deoxy-α-D-mannopyranoside (**4**)
Exp: MDS-058

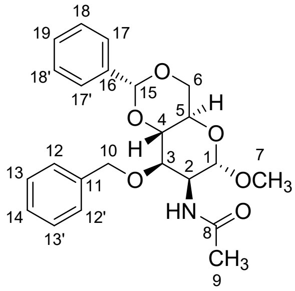

The procedure was adapted from Petrakova et al.[10] and implemented with minor modifications.

To a mixture of methyl (10*R*)-2-acetamido-4,6-O-benzylidene-2-deoxy-α-D-mannopyranoside **3** (unpurified, obtained from the experiment described above), barium oxide (21.9 g, 143 mmol, 3.00 equiv.), and barium hydroxide octahydrate (7.5 g, 24 mmol, 0.50 equiv.) was added in DMF (0.25 L, 0.19 M) and benzyl bromide (8.53 mL, 71.3 mmol, 1.50 equiv.). The resulting suspension was stirred at 23 °C for 16 h before the reaction mixture was filtered through a pad of celite. The solvent of the resulting solution was evaporated *in vacuo* to give a colorless oily residue which was taken up in CH_2Cl_2 (0.5 L) and washed with an aq. LiCl soln. (5%, 3 × 100 mL). The organic layer was then dried over anhydrous $MgSO_4$, filtered, and the solvent evaporated *in vacuo* to obtain an amorphous colorless solid, which was used in the next step without further purification. For analytical purposes, the reaction was also carried out on smaller scale (*Exp: MDS-027*): starting from purified methyl (10*R*)-2-acetamido-4,6-O-benzylidene-2-deoxy-α-D-mannopyranoside (**3**, 1005 mg, 3.108 mmol), and the product was purified by column chromatography (CH_3OH/CH_2Cl_2, 1:75) to obtain the desired compound **4** as a colorless amorphous solid (92%, 1180 mg, 2.854 mmol).

***R*$_F$** (CH_3OH/CH_2Cl_2, 1:75) = 0.19.

HR-ESI-MS (+) (MDS-026, $CH_3OH/CHCl_3$, 3:2) calculated for $C_{23}H_{27}O_6NNa^+$ $[M+Na]^+$: 436.17306, found: 436.17292.

^{1}H-NMR (MDS-026, 500 MHz, CD_3OD) δ 7.52 – 7.43 (m, 2H, C17H and C17’H), 7.42 – 7.20 (m, 8H, C12H, C12’H, C13H, C13’H, C14H, C18H, C18’H and C19H), 5.63 (s, 1H, C15H), 4.69 – 4.63 (m, 2H, C1H and C10H_a), 4.61 (dd, *J* = 4.4, 1.4 Hz, 1H, C2H), 4.56 (d, *J* = 11.8 Hz, 1H, C10H_b), 4.22 (dd, *J* = 10.1, 4.7 Hz, 1H, C6H_{eq}), 4.04 – 3.95 (m, 2H, C3H and C4H), 3.86 (t, *J* = 10.2 Hz, 1H, C6H_{ax}), 3.76 (ddd, *J* = 10.3, 8.6, 4.7 Hz, 1H, C5H), 3.37 (s, 3H, C7H_3), 2.04 (s, 3H, C9H_3).

^{13}C{^{1}H}-NMR (MDS-026, 126 MHz, CD_3OD) δ 173.70 (1C, C8), 139.50 (1C, C11), 139.20 (1C, C16), 129.95 (1C, C19), 129.26 (2C, C13 and C13’ or C18 and C18’), 129.11 (2C, C13 and C13’ or C18 and C18’), 128.90 (2C, C12 and C12’), 128.66 (1C, C14), 127.36 (2C, C17 and C17’), 103.14 (1C, C15), 102.69 (1C, C1), 79.52 (1C, C4), 74.72 (1C, C3), 72.56 (1C, C10), 69.77 (1C, C6), 64.80 (1C, C5), 55.34 (1C, C7), 51.80 (1C, C2), 22.51 (1C, C9).

Methyl 2-acetamido-3-*O*-benzyl-2-deoxy-α-D-mannopyranoside (**5**) *Exp: MDS-062*

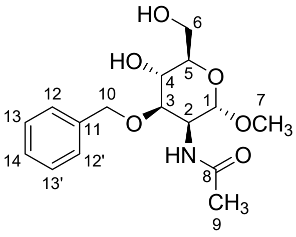

The procedure was adapted from Scaffidi et al.[11] and implemented with minor modifications.

Methyl (10*R*)-2-acetamido-3-*O*-benzyl-4,6-*O*-benzylidene-2-deoxy-α-D-mannopyranoside **4** (unpurified, obtained from the experiment described above) was dissolved in H_2O/AcOH (1:4, 125 mL, 0.380 M). The resulting solution was heated to 70 °C for 4 h before the solvent was evaporated *in vacuo*. The resulting oily residue was then purified by column chromatography (CH_3OH/CH_2Cl_2, 1:20) to give the desired product as an amorphous colorless solid in a yield of 61% (9.42 g, 29.0 mmol) over 3 steps starting from **SI.II**.

The reaction (*Exp: MDS-029*) starting from purified methyl (10*R*)-2-acetamido-3-*O*-benzyl-4,6-*O*-benzylidene-2-deoxy-α-D-mannopyranoside (**4**, 1075 mg, 2.60 mmol) delivered the desired compound **5** in 93% yield (783 mg, 2.41 mmol).

$\boldsymbol{R}_F$ (CH_3OH/CH_2Cl_2, 1:20) = 0.17.

HR-ESI-MS (+) (MDS-047, CH_3OH) calculated for $C_{16}H_{23}O_6NNa^+$ $[M+Na]^+$: 348.14176, found: 348.14183.

FT-IR (MDS-047, CH_3OH) $\tilde{\nu}$ 3300m, 3065w, 3032w, 2929w, 2838w, 1652s, 1544m, 1498m, 1454m, 1375m, 1294m, 1201m, 1126s, 1066s, 1041s, 969m, 899w, 849w, 835w, 800w, 740m, 699s, 666m, 605m, 581m, 498m, 462m.

$[\alpha]_D^{24.6}$ = −0.23 (MDS-047, c = 1.32, CH_3OH).

Melting range (MDS-047) 48.6–49.5 °C.

^{1}H-NMR (MDS-028, 500 MHz, CD_3OD) δ 7.40–7.34 (m, 2H, C12H and C12'H), 7.34–7.28 (m, 2H, C13H and C13'H), 7.28–7.22 (m, 1H, C14H), 4.70 (d, J = 11.2 Hz, 1H, C10H_a), 4.62 (d, J = 1.6 Hz, 1H, C1H), 4.56 (dd, J = 4.7, 1.6 Hz, 1H, C2H), 4.48 (d, J = 11.2 Hz, 1H, C10H_b), 3.83–3.80 (m, 2H, C6H_a and C6H_b), 3.75 (dd, J = 9.5, 4.6 Hz, 1H, C3H), 3.68 (t, J = 9.7 Hz, 1H, C4H), 3.56–3.51 (m, 1H, C5H), 3.36 (s, 3H, C7H_3), 1.99 (s, 3H, C9H_3).

^{13}C{^{1}H}-NMR (MDS-028, 126 MHz, CD_3OD) δ 173.42 (1C, C8), 139.67 (1C, C11), 129.23 (2C, C13 and C13'), 129.13 (2C, C12 and C12'), 128.60 (1C, C14), 101.80 (1C, C1), 78.49 (1C, C3), 73.91 (1C, C5), 72.22 (1C, C10), 67.17 (1C, C4), 62.23 (1C, C6), 55.25 (1C, C7), 50.46 (1C, C2), 22.51 (1C, C9).

VI.III. Methyl 2-acetamido-2-deoxy-3-*O*-benzyl-4-*O*-isobutyryl-6-iodo-6-deoxy-α-D-mannopyranoside (7)

The title compound was synthesized in two consecutive steps:

Methyl 2-acetamido-2-deoxy-3-*O*-benzyl-6-iodo-6-deoxy-α-D-mannopyranoside (**6**) *Exp: MDS-070*

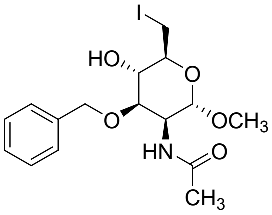

The procedure was adapted from Skaanderup et al.[12] and implemented with minor modifications.

Methyl 2-acetamido-2-deoxy-3-*O* -benzyl-α-D-mannopyranoside (6.86 g, 21.1 mmol, 1.00 equiv.), triphenylphosphane (8.30 g, 31.6 mmol, 1.50 equiv.), and imidazole (2.87 g, 42.2 mmol, 2.00 equiv.) were dissolved in THF (0.2 L, 0.1 M). The resulting solution was heated to reflux (85 °C) before a solution of iodine (8.03 g, 31.6 mmol, 1.50 equiv.) in THF (50 mL) was added dropwise over the course of 40 min. The resulting red/brown reaction mixture was then held at reflux for 3.5 h before it was allowed to cool to 23 °C. The mixture was filtered under vacuum, and the filter cake was rinsed with THF (2 × 50 mL). The filtrate was concentrated *in vacuo* to give an oily brown/red residue which was taken up in CH_2Cl_2 (300 mL) and washed with aq. HCl soln. (0.1 M, 3 × 100 mL), aq. $NaHCO_3$ soln.(5%, 1 × 100 mL), aq. $Na_2S_2O_3$ soln. (10%, 1 × 100 mL), and brine (1 × 100 mL). The organic layer was then dried over anhydrous $MgSO_4$, filtered, and the solvent evaporated *in vacuo* to obtain the reaction product **6** as a mixture with PPh_3O (identified by TLC and ^{1}H-NMR) in the form of a pale yellow solid, which was used in the next step without further purification.

$\boldsymbol{R}_F$ (CH_3OH/CH_2Cl_2, 1:60) = 0.21

HR-ESI-MS (+) (MDS-033, $CH_3OH/CHCl_3$, 3:2) calculated for $C_{16}H_{22}O_5NINa^+$ [M+Na]$^+$: 458.04349, found: 458.04338.

Methyl 2-acetamido-2-deoxy-3-*O*-benzyl-4-*O*-isobutyryl-6-iodo-6-deoxy-α-D-mannopyranoside (**7**) *Exp: MDS-071*

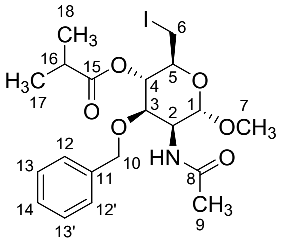

The residue **6** was dissolved in CH_2Cl_2 (0.2 L, 0.1 M) and the resulting solution was cooled to 0 °C before pyridine (3.40 mL, 42.2 mmol, 2.00 equiv.) and then isobutyryl chloride (3.32 mL, 31.7 mmol, 1.50 equiv.) were added. After 10 min, the reaction was allowed to warm to 23 °C and it was stirred for 12 h. The reaction mixture was then washed with aq. HCl soln. (0.1 M, 2 × 100 mL) and aq. $NaHCO_3$ soln. (5%, 3 × 100 mL) before the organic layer was dried over anhydrous $MgSO_4$, filtered, and the solvent evaporated in vacuo to obtain the crude product as pale a yellow residue which was purified by column chromatography (EtOAc/hexanes, 1:2.5) to give the desired product as a colorless amorphous solid (64%, 6.78 g, 13.4 mmol) over 2 steps starting from methyl 2-acetamido-2-deoxy-3-*O*-benzyl-α-D-mannopyranoside.

$\boldsymbol{R}_F$ (EtOAc/hexanes, 2:1) = 0.67

HR-ESI-MS (+) (MDS-034, $CH_3OH/CHCl_3$, 3:2) calculated for $C_{20}H_{28}O_6NINa^+$ $[M+Na]^+$: 528.08535, found: 528.08541.

FT-IR (MDS-038, CH_3OH) $\tilde{\nu}$ 3279w, 3063w, 2972w, 2932w, 1802w, 1738m, 1650m, 1546m, 1498w, 1468m, 1455m, 1373m, 1322w, 1290m, 1252w, 1188m, 1151s, 1127s, 1063s, 1027s, 971m, 897w, 795w, 773m, 746m, 734m, 698m, 632m, 605m, 583w, 554m, 503m, 481m, 465m, 434m.

$[\alpha]_D^{23.7}$ = +4.15 (MDS-038, c = 0.60, CH_3OH).

Melting range (MDS-038) 50.6–51.4 °C.

^{1}H-NMR (MDS-034, 500 MHz, CD_3OD) δ 7.33–7.21 (m, 5H, C12H, C12'H, C13H, C13'H, and C14H), 5.03 (t, J = 9.6 Hz, 1H, C4H), 4.68 (d, J = 1.7 Hz, 1H, C1H), 4.64–4.58 (m, 2H, C2H and C10H_a), 4.36 (d, J = 11.4 Hz, 1H, C10H_b), 3.91 (dd, J = 9.6, 4.8 Hz, 1H, C3H), 3.74 (td, J = 9.6, 2.3 Hz, 1H, C5H), 3.45 (s, 3H, C7H_3), 3.28 (dd, J = 10.7, 2.4 Hz, 1H, C6H_a), 3.20 (dd, J = 10.7, 9.6 Hz, 1H, C6H_b), 2.55 (hept, J = 7.0 Hz, 1H, C16H), 2.01 (s, 3H, C5H_3), 1.13 (d, J = 7.0 Hz, 3H, C17H_3 or C18H_3), 1.12 (d, J = 7.0 Hz, 3H, C17H_3 or C18H_3).

^{13}C{^{1}H}-NMR (MDS-034, 126 MHz, CD_3OD) δ 177.80 (1C, C15), 173.66 (1C, C18), 139.15 (1C, C11), 129.25 (2C, C13 and C13'), 128.87 (2C, C12 and C12'), 128.72 (1C, C14), 101.68 (1C, C1), 75.80 (1C, C3), 72.25 (1C, C4), 72.10 (1C, C10), 71.95 (1C, C5), 55.81 (1C, C7), 50.59 (1C, C2), 35.23 (1C, C16), 22.46 (1C, C9), 19.33 (1C, C17 or C18), 19.25 (1C, C17 or C18), 3.99 (1C, C6).

VI.IV. (3*S*,4*R*,5*S*,6*S*)-5-Acetamido-4-(benzyloxy)-6-methoxy-2-methylenetetrahydro-2*H*-pyran-3-yl isobutyrate (2) *Exp: MDS-114*

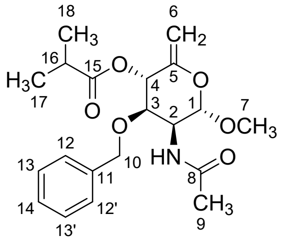

The procedure was adapted from Sieber et al.[13] and Hedberg et al.[14] and was implemented with minor modifications.

A mixture of methyl 2-acetamido- 2-deoxy-3-*O*-benzyl-4-*O*-isobutyryl-6-iodo-6-deoxy-α-D-mannopyranoside (**7**, 2.10 g, 4.16 mmol, 1.00 equiv.) and AgF (1.59 g, 12.5 mmol, 3.00 equiv.) was suspended in pyridine (8 mL, 0.5 M). The reaction mixture was stirred under the exclusion of light at 23 °C for 24 h. It was then diluted with EtOAc (0.2 L) and filtered through a pad of celite. The celite was then rinsed with EtOAc (3 × 50 mL) and the resulting suspension was vacuum filtered, washed with aq. HCl soln. (0.1 M, 2 × 100 mL), aq. $NaHCO_3$ soln. (5%, 1 × 100 mL), brine (1 × 50 mL), aq. $CuSO_4$ (sat., 2 × 50 mL), brine (2 × 50 mL), aq. $Na_2S_2O_3$ soln. (10%, 1 × 100 mL), and brine (1 × 50 mL). The organic layer was dried over anhydrous $MgSO_4$, filtered, and the solvent evaporated *in vacuo* to obtain a pale-yellow oil. Purification by column chromatography (EtOAc/hexanes, 1:2) afforded the desired product as a colorless solid (69%, 1.08 g, 2.87 mmol). Single crystals were obtained by vapor diffusion (solvent: EtOAc/*n*-hexane, 1:1.5; antisolvent: *n*-hexane).

R_F (EtOAc/hexanes, 1:2) = 0.18

HR-ESI-MS (+) (MDS-067, $CH_3OH/CHCl_3$, 3:2) calculated for $C_{20}H_{27}O_6NNa^+$ $[M+Na]^+$: 400.17306, found: 400.17279.

FT-IR (MDS-039, CH_3OH) $\tilde{\nu}$ 3283w, 3065w, 2973w, 2934w, 1743s, 1659s, 1548m, 1470w, 1455w, 1373m, 1293w, 1255m, 1192m, 1156s, 1119s, 1069s, 981w, 908w, 860w, 739m, 699m, 610w.

$[\alpha]_D^{24.6}$ = −0.32 (MDS-039, c = 0.79, CH_3OH).

Melting range (MDS-074, single crystals) 117.3–118.0 °C

^{1}H-NMR (MDS-039, 500 MHz, CD_3OD) δ 7.35–7.23 (m, 5H, C12H, C12'H, C13H, C13'H, and C14H), 5.61 (dt, J = 9.2, 1.8 Hz, 1H, C4H), 4.76 (d, J = 2.7 Hz, 1H, C1H), 4.70 (t, J = 1.6 Hz, 1H, C6H_a), 4.64–4.60 (m, 2H, C2H and C10H_a), 4.48 (t, J = 1.6 Hz, 1H, C6H_b), 4.45 (d, J = 11.6 Hz, 1H, C10H_b), 3.91 (dd, J = 9.1, 4.6 Hz, 1H, C3H), 3.43 (s, 3H, C7H_3), 2.62 (hept, J = 7.0 Hz, 1H, C16H), 1.99 (s, 3H, C9H_3), 1.19 (d, J = 7.0 Hz, 3H, C17H_3 or C18H_3), 1.18 (d, J = 7.0 Hz, 3H, C17H_3 or C18H_3).

^{13}C{^{1}H}-NMR (MDS-039, 126 MHz, CD_3OD) δ 177.42 (1C, C15), 173.75 (1C, C18), 153.92 (1C, C5), 139.17 (1C, C11), 129.31 (2C, C13 and C13'), 129.02 (2C, C12 and C12'), 128.81 (1C, C14), 102.71 (1C, C1), 97.22 (1C, C6), 75.73 (1C, C3), 72.41 (1C, C10), 69.76 (1C, C4), 55.84 (1C, C7), 50.69 (1C, C2), 35.26 (1C, C16), 22.46 (1C, C9), 19.32 (2C, C17 and C18).

VI.V. (5*S*,6*S*,7*R*,8*S*)-6-Acetamido-7-(benzyloxy)-5-methoxy-4-oxaspiro[2.5]octan-8-yl isobutyrate (8) *Exp: MDS-125*

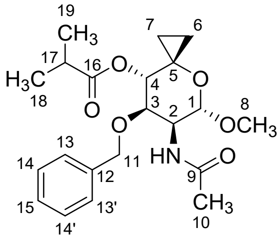

The procedure was based on literature from Lorenz et al.[15] and implemented with modifications.

A solution of trifluoroacetic acid (1.01 mL, 13.5 mmol, 5.00 equiv.) in CH_2Cl_2 (10 mL) was added dropwise to a cooled (0 °C) solution of $ZnEt_2$ (1 M in *n*-hexane, 13.5 mL, 13.5 mmol, 5.00 equiv.) in 25 mL CH_2Cl_2 over the course of 5 min. After stirring for 20 min at 0 °C, a solution of CH_2I_2 (1.31 mL, 16.2 mmol, 6.00 equiv.) in CH_2Cl_2 (10 mL) was slowly added to the reaction over the course of 5 min. After an additional 20 min of stirring at 0 °C, a solution of olefin **2** (1.02 g, 2.71 mmol, 1.00 equiv.) in CH_2Cl_2 (20 mL) was added to the reaction (c_{final} = 35 mM) and the resulting mixture was vigorously stirred for 10 min before it was allowed to warm to 23 °C. After stirring the reaction mixture for 36 h at 23 °C, aq. HCl soln. (0.1 M, 20 mL) was added under N_2 before the mixture was diluted with CH_2Cl_2 (200 mL) and washed with aq. HCl soln. (0.1 M, 1 × 100 mL), aq. $NaHCO_3$ soln. (5%, 1 × 50 mL), aq. $Na_2S_2O_3$ soln. (10%, 1 × 50 mL), and brine (1 × 50 mL). The organic layer was dried over anhydrous $MgSO_4$, filtered, and the solvent was removed *in vacuo* to obtain an oily, orange residue which was purified by column chromatography (EtOAc/hexanes, gradient: 1:1.8 to 1:1.3) to give the desired product as a colorless solid (81%, 0.856 g, 2.19 mmol). Single crystals were obtained by vapor diffusion (solvent: EtOAc/*n*-hexane, 1:1.3; antisolvent: *n*-hexane).

$\boldsymbol{R}_F$ (EtOAc/hexanes, 1:1) = 0.26

HR-ESI-MS (+) (MDS-069, $CH_3OH/CHCl_3$, 3:2) calculated for $C_{21}H_{29}O_6NNa^+$ $[M+Na]^+$: 414.18871, found: 414.18845.

FT-IR (MDS-069, CH_3OH) $\tilde{\nu}$ 3286w, 3065w, 2973w, 2935w, 2877w, 1732m, 1656m, 1547m, 1469m, 1455m, 1388m, 1372m, 1330w, 1254m, 1196m, 1153s, 1119m, 1100m, 1072m, 1037s, 993m, 865w, 741w, 700m, 593w, 529w, 472w.

$[\alpha]_D^{24.2}$ = +151.45 (MDS-069, c = 0.86, CH_3OH).

Melting range (MDS-073, single crystals) 98.5–99.5 °C

^{1}H-NMR (MDS-069, 500 MHz, CD_3OD) δ 7.40–7.25 (m, 5H, C13$\underline{H}$, C13'$\underline{H}$, C14$\underline{H}$, C14'$\underline{H}$, and C15$\underline{H}$), 4.71–4.64 (m, 3H, C1$\underline{H}$, C4$\underline{H}$, and C11$\underline{H}_a$), 4.53 (d, J = 11.9 Hz, 1H, C11$\underline{H}_b$), 4.17 (dd, J = 8.0, 3.7 Hz, 1H, C2$\underline{H}$), 3.79 (t, J = 3.7 Hz, 1H, C3$\underline{H}$), 3.36 (s, 3H, C8$\underline{H}_3$), 2.63 (hept, J = 7.0 Hz, 1H, C17$\underline{H}$), 1.89 (s, 3H, C10$\underline{H}_3$), 1.19 (d, J = 6.9 Hz, 6H, C18$\underline{H}_3$ and C19$\underline{H}_3$), 0.92–0.87 (m, 1H, C7$\underline{H}_a$), 0.84–0.75 (m, 2H, C6$\underline{H}_a$, C7$\underline{H}_b$), 0.72–0.66 (m, 1H, C6$\underline{H}_b$).

^{13}C{^{1}H}-NMR (MDS-069, 126 MHz, CD_3OD) δ 178.06 (1C, C16), 172.99 (1C, C9), 139.16 (1C, C12), 129.50 (2C, C13 and C13' or C14 and C14'), 129.43 (2C, C13 and C13' or C14 and C14'), 129.00 (1C, C15), 101.11 (1C, C1), 76.53 (1C, C3), 73.32 (1C, C11), 72.86 (1C, C4), 57.76 (1C, C5), 56.57 (1C, C8), 51.43 (1C, C2), 35.26 (1C, C17), 22.55 (1C, C10), 19.31 (2C, C18 and C19), 12.14 (1C, C7 or C6), 9.13 (1C, C6 or C7).

VI.VI. (5*S*,6*S*,7*R*,8*S*)-6-Acetamido-7-hydroxy-5-methoxy-4-oxaspiro[2.5]octan-8-yl isobutyrate (9) *Exp: MDS-124*

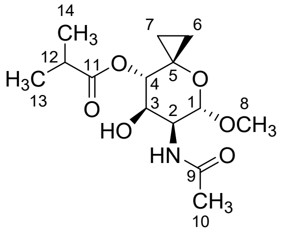

The procedure was adapted from Binkley et al.[16] and implemented with minor modifications.

To a mixture of (5*S*,6*S*,7*R*,8*S*)-6-acetamido-7-(benzyloxy)-5-methoxy-4-oxaspiro[2.5]octan-8-yl isobutyrate (**8**, 202 mg, 0.516 mmol, 1.00 equiv.), *N*-bromosuccinimide (110 mg, 0.619 mmol, 1.20 equiv.), and $CaCO_3$ (232 mg, 2.32 mmol, 4.50 equiv.) was added H_2O/CCl_4 (1:10,2.2 mL, 0.25 M). The resulting suspension was sparged with N_2 for 10 min before it was irradiated (2 opposing LEDs ,10 – 15 cm distance from the flask, λ = 456 nm, 2 × 40 W) for 20 min at 23 °C with vigorous stirring and fan cooling of the flask. The suspension was then filtered under vacuum, and the solvent of the filtrate was evaporated in vacuo to obtain a pale yellow, oily residue, which was purified by column chromatography (CH_3OH/CH_2Cl_2, 1:32) to give the desired product as a colorless solid (77%, 119 mg, 0.395 mmol).

$\boldsymbol{R}_F$ (CH_3OH/CH_2Cl_2, 1:32) = 0.22

HR-ESI-MS (+) (MDS-124, $CH_3OH/CHCl_3$, 3:2) calculated for $C_{14}H_{23}O_6NNa^+$ [*M*+Na]+: 324.14176, found: 324.14184.

FT-IR (MDS-096, CH_3OH) $\tilde{\nu}$ 3300w, 3094w, 2974w, 2936w, 2878w, 1732m, 1648m, 1544m, 1470m, 1447m, 1387m, 1373m, 1351m, 1316m, 1252m, 1192s, 1148s, 1116m, 1099m, 1069m, 1037s, 1017m, 953m, 929m, 908m, 869w, 817w, 703m, 659m, 598m, 527m, 468m, 435m, 407w.

$[\alpha]_D^{24.0}$ = +108.36 (MDS-096, c = 1.14, CH_3OH).

Melting range (MDS-124) 141.1–141.7 °C

^{1}H-NMR (MDS-096, 400 MHz, CD_3OD) δ 4.69 (d, *J* = 8.1 Hz, 1H, C1H), 4.49 (d, *J* = 3.9 Hz, 1H, C4H), 4.13 (dd, *J* = 8.1, 3.4 Hz, 1H, C2H), 3.94 (t, *J* = 3.6 Hz, 1H, C3H), 3.37 (s, 3H, C8H_3), 2.65 (hept, *J* = 7.0 Hz, 1H, C12H), 1.99 (s, 3H, C10H_3), 1.20 (d, *J* = 7.0 Hz, 3H, C13H_3 or C14$_3$), 1.19 (d, *J* = 7.0 Hz, 3H, C13H_3 or C14$_3$), 0.94–0.87 (m, 1H, C6H_a or C7H_a), 0.87 – 0.74 (m, 2H, C6H_b and C7H_a or C6H_a and C7H_b), 0.69 – 0.62 (m, 1H, C6H_b or C7H_b).

^{13}C{^{1}H}-NMR (MDS-096, 101 MHz, CD_3OD) δ 178.05 (1C, C11), 173.26 (1C, C9), 101.02 (1C, C1), 76.25 (1C, C4), 69.67 (1C, C3), 57.69 (1C, C5), 56.62 (1C, C8), 52.28 (1C, C2), 35.26 (1C, C12), 22.66 (1C, C10), 19.37 (1C, C13 or C14), 19.33 (1C, C13 or C14), 12.29 (1C, C6 or C7), 8.87 (1C, C6 or C7).

VI.VII. Methyl 2-acetamido-2-deoxy-4-*O*-isobutyryl-4-*O*-demethyl-α-D-noviopyranoside (1) *Exp: MDS-107*

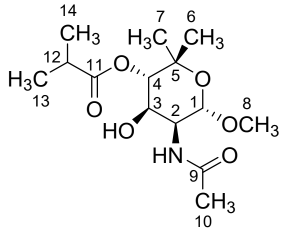

To a solution of alcohol **9** (107 mg, 0.355 mmol, 1.00 equiv.) in AcOH (2.0 mL, 0.18 mM) was added PtO_2 (168 mg, 0.710 mmol, 2.00 equiv.) and the resulting mixture was flushed with H_2 in a pressure reactor before it was pressurized with H_2 (50 bar). The resulting mixture was stirred at 23 °C for 14 d. Then aq. $NaHCO_3$ soln. (sat.). was added until pH ~ 8 before the mixture was extracted with CH_2Cl_2 (3 × 30 mL). The combined organic layers were then dried over anhydrous $MgSO_4$, filtered, and the solvent was evaporated in vacuo to give a colorless solid (88%, 95 mg, 0.313 mmol, 84% purity by qNMR). For analytical purposes an aliquot was purified using preparative HPLC (isocratic, 38% B, t_R = 55.5 min).

R_F (CH_3OH/CH_2Cl_2, 1:32) = 0.22

HR-ESI-MS (+) (MDS-107, $CH_3OH/CHCl_3$, 3:2) calculated for $C_{14}H_{25}O_6NNa^+$ $[M+Na]^+$: 326.15741, found: 326.15688.

FT-IR (MDS-098, CH_3OH) $\tilde{\nu}$ 3320m, 3075w, 2977m, 2934m, 2853w, 1736s, 1656s, 1547m, 1470m, 1446m, 1387m, 1371m, 1260m, 1197s, 1154s, 1120s, 1097m, 1059s, 1040s, 963m, 928w, 801w, 764w, 714w, 668w, 597w, 553w, 525w, 509w, 469w.

$[\alpha]_D^{23.3}$ = +52.85 (MDS-098, c = 0.19, CH_3OH).

Melting range 51.5–52.5 °C (MDS-098)

^{1}H-NMR (MDS-098, 500 MHz, CD_3OD) δ 4.89 (d, J = 6.9 Hz, 1H, C4H), 4.69 (d, J = 5.3 Hz, 1H, C1H), 4.16 (dd, J = 5.3, 4.1 Hz, 1H, C2H), 4.01 (dd, J = 6.8, 4.1 Hz, 1H, C3H), 3.41 (s, 3H, C8H_3), 2.64 (hept, J = 7.0 Hz, 1H, C12H), 2.00 (s, 3H, C10H_3), 1.39 (s, 3H, C6H_3), 1.27 (s, 3H, C7H_3), 1.20 (d, J = 7.0 Hz, 3H, C13H_3 or C14H_3), 1.19 (d, J = 7.0 Hz, 3H, C13H_3 or C14H_3).

^{13}C{^{1}H}-NMR (MDS-098, 126 MHz, CD_3OD) δ 177.97 (1C, C11), 173.78 (1C, C9), 100.23 (1C, C1), 76.99 (1C, C5), 75.75 (1C, C4), 67.97 (1C, C3), 56.20 (1C, C8), 53.56 (1C, C2), 35.38 (1C, C12), 26.54 (1C, C6), 25.30 (1C, C7), 22.61 (1C, C10), 19.40 (1C, C13 or C14), 19.26 (1C, C13 or C14).

VII. Purity determination of crude 2-acetylnoviosamine 1 by qNMR

Purity of crude 2-acetylnoviosamine **1** was determined in three independent quantitative ^{1}H-NMR analyses with 1,3,5-trimethoxybenzene (*Sigma-Aldrich*, ≥99%) as internal standard. The measurements were conducted on a 500 MHz spectrometer in CD_3OD. Inversion recovery experiments were carried out (figure S8) to estimate the T_1 times of the resonances at 4.92 ppm (d, ***J*** = 6.9 Hz, 1H, C4H, compound **1**) and 6.09 ppm (s, 3H, CH arom., 1,3,5-trimethoxybenzene). ^{1}H-NMR measurements were then conducted with a relaxation delay of 47.2 s (7 times longest T_1 (τ / ln2) minus the acquisition time (3.3 s)) so that more than 99.9% of the spin magnetization is relaxed. The weight of internal standard and compound **1** for each measurement is summarized in table S2. The integrals and their absolute values used for purity calculation are depicted in figure S9 to figure S11. Reasonable integration borders were estimated and set by hand and do not include ^{13}C satellites. The results of the purity determination are summarized in table S2.

*Table S1 Masses of crude 2-acetylnoviosamine **1** and internal standard (1,3,5-trimethoxybenzene) used for each qNMR measurement.*

Measurement	$m_{crude\ \mathbf{1}}$ [g]	m_{IntStd} [g]
1	0.013354	0.007028
2	0.011106	0.007432
3	0.012459	0.009220

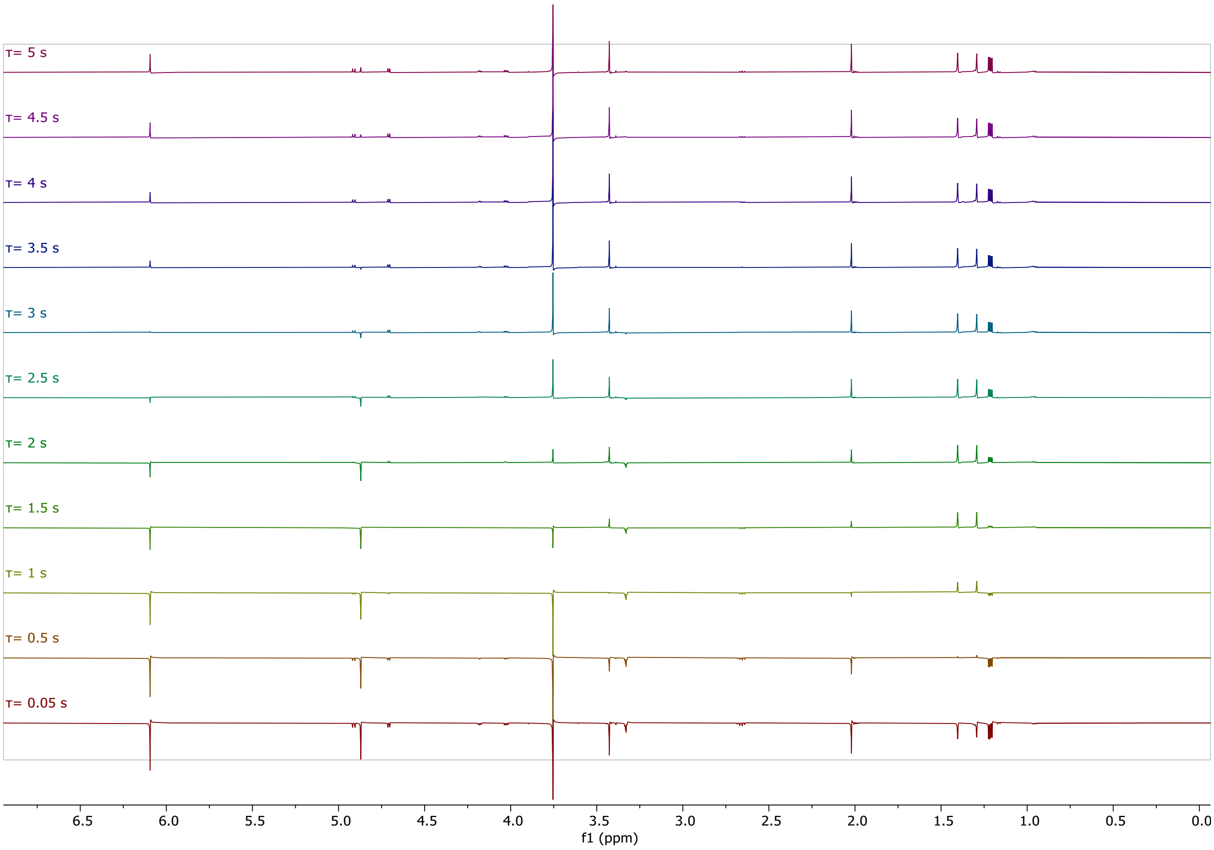

Figure S8: Stack of inversion recovery experiment spectra. The used delay time τ is shown on top left of the spectra.

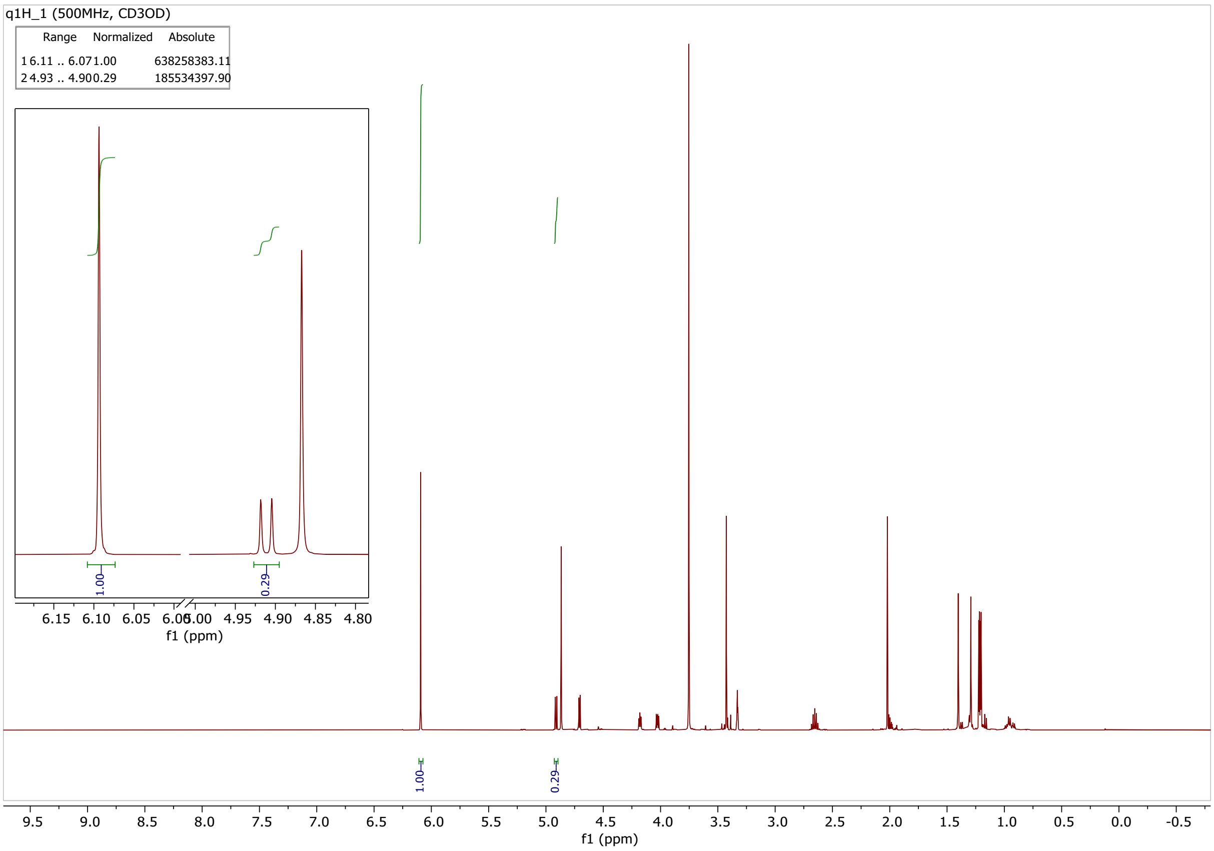

Figure S9: Integration values and signals for qNMR measurement 1.

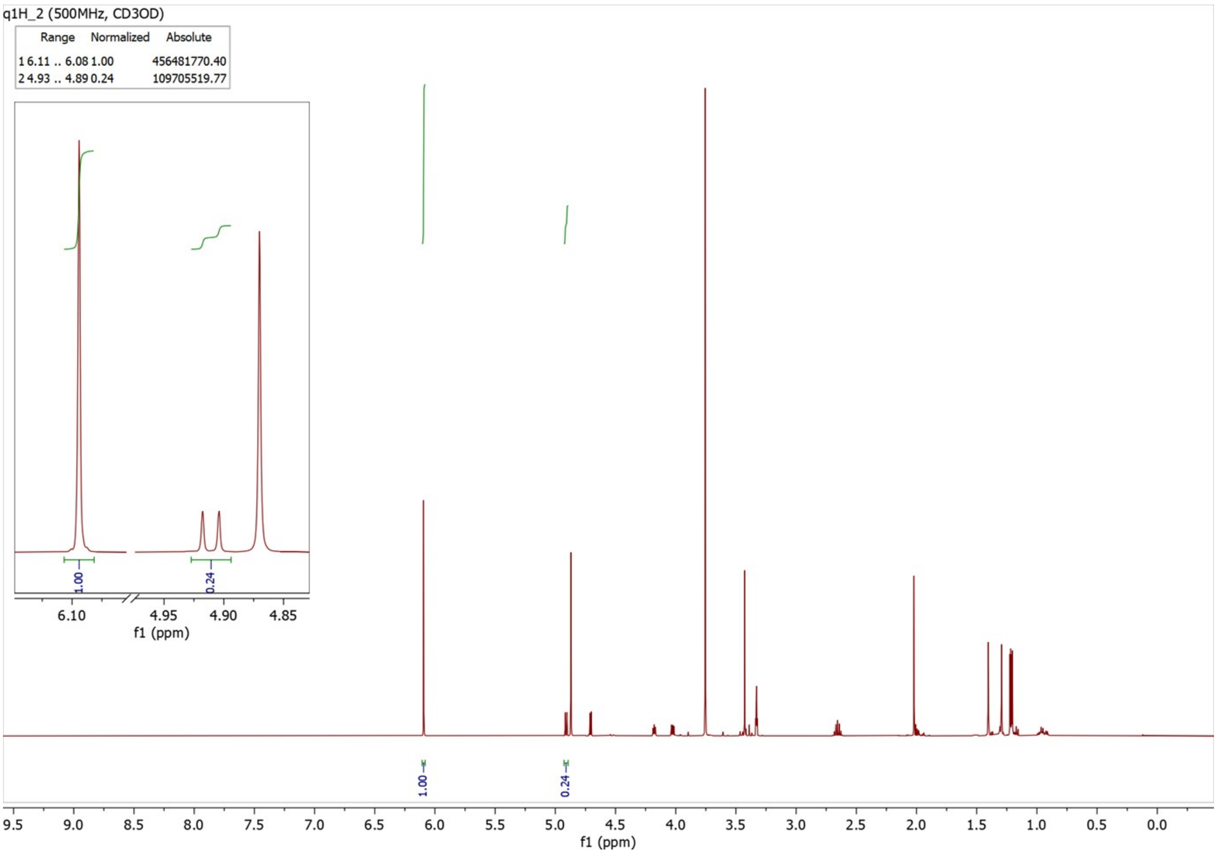

Figure S10: Integration values and signals for qNMR measurement 2.

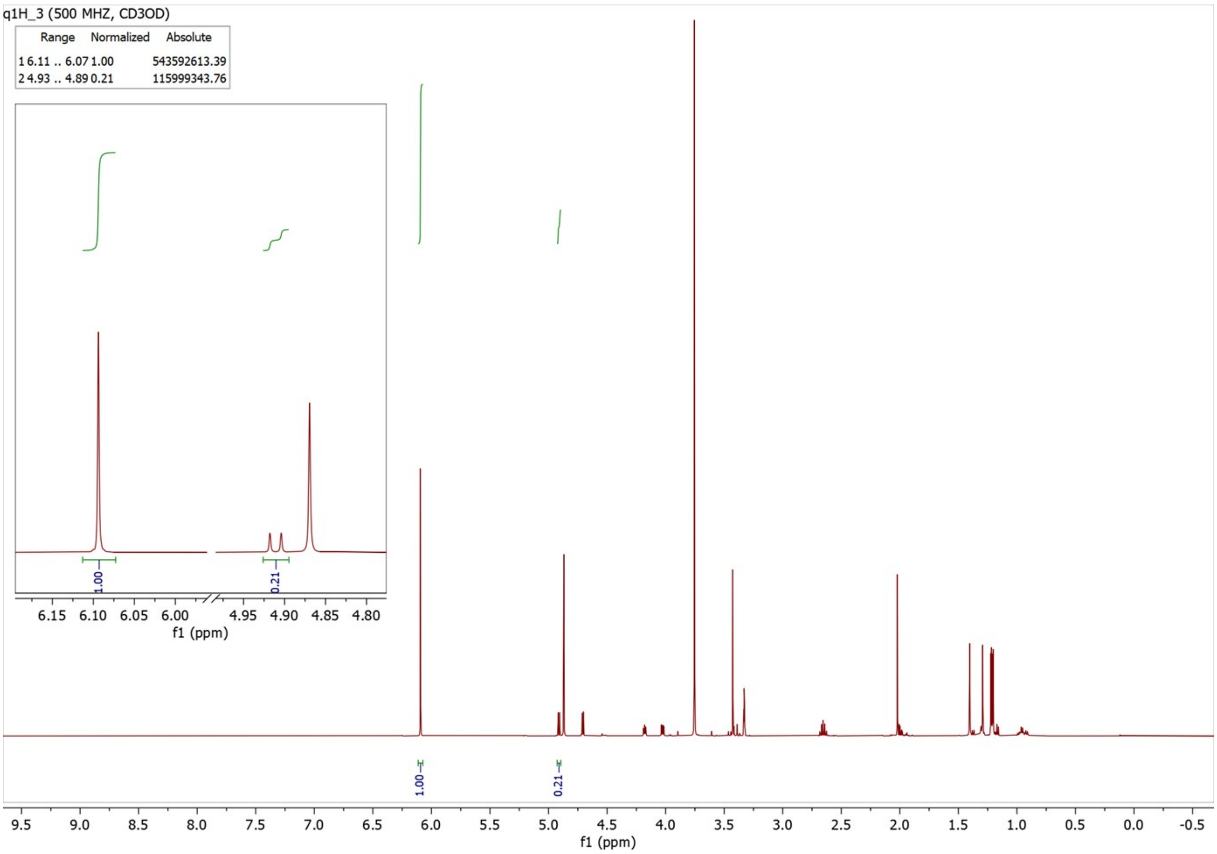

Figure S11: Integration values and signals for qNMR measurement 3.

*Table S2: Results purity determination of crude 2-acetylnoviosamine **1** by qNMR*

Measurement	Calculated purity crude **1** [%]	Average purity crude **1** [%]	Standard deviation purity crude **1** [%]
1	81.95		
2	86.14	84.23	2.12
3	84.59		

VIII. NMR spectra

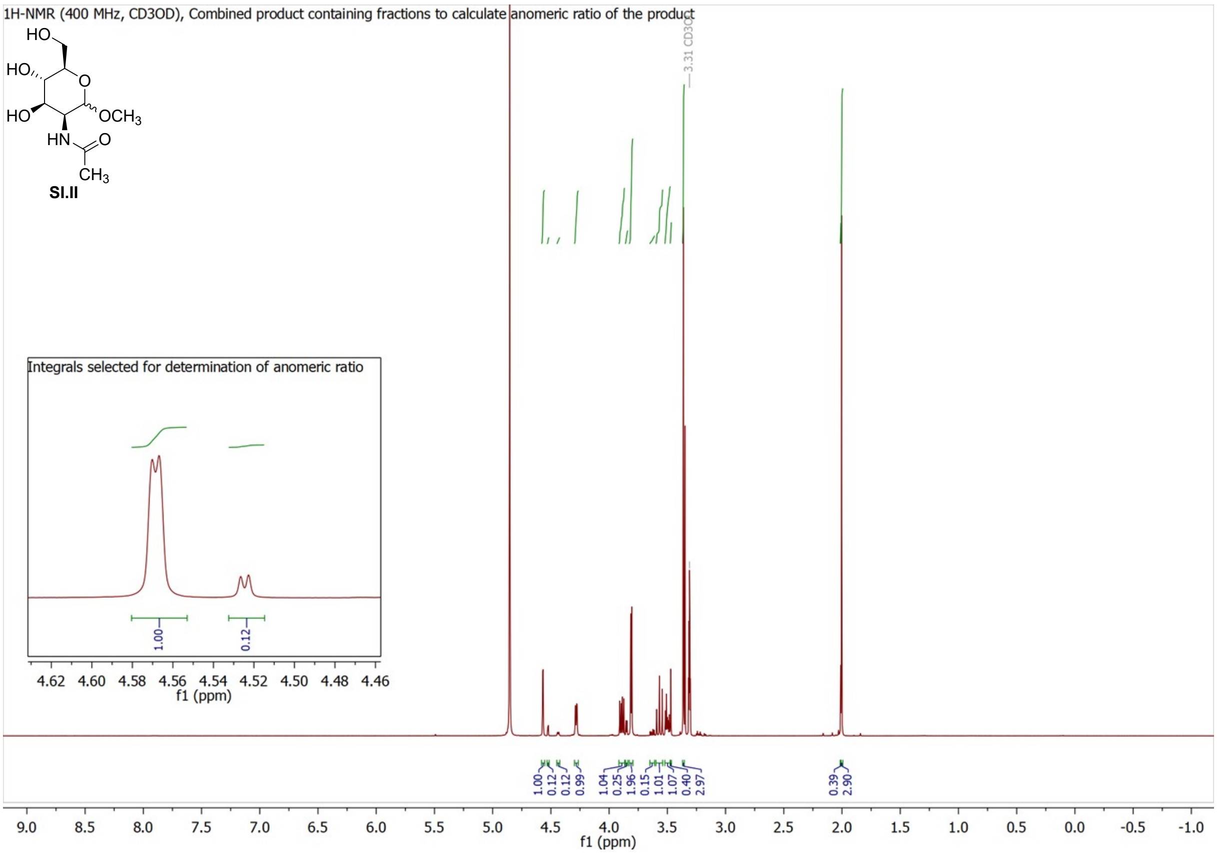

*Figure S12: ^{1}H-NMR spectrum of combined product containing fractions to calculate anomeric ratio of product **SI.II**.*

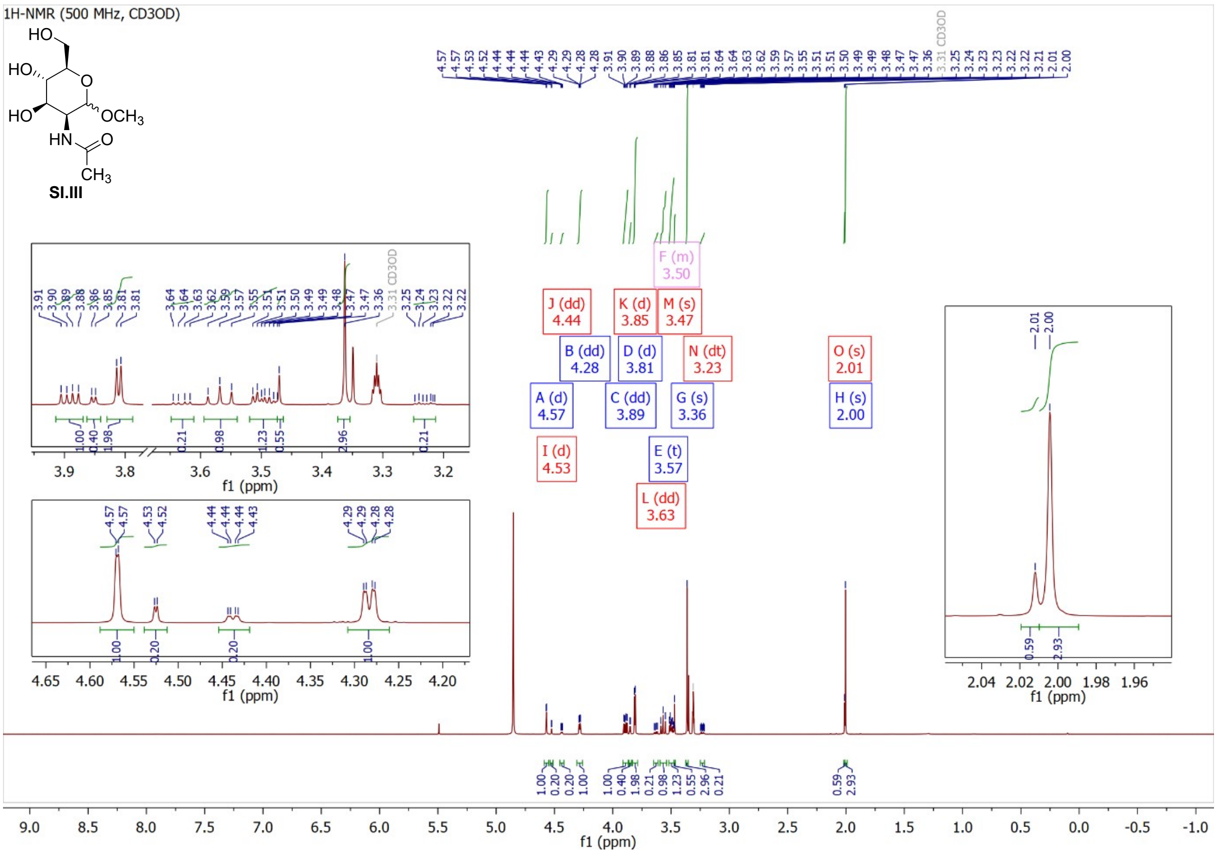

*Figure S13: ^{1}H-NMR spectrum of minor anomer enriched fractions of product **SI.III**. Color code resonnances: blue = α-anomer, red = β-anomer, pink = overlap α- & β-anomers.*

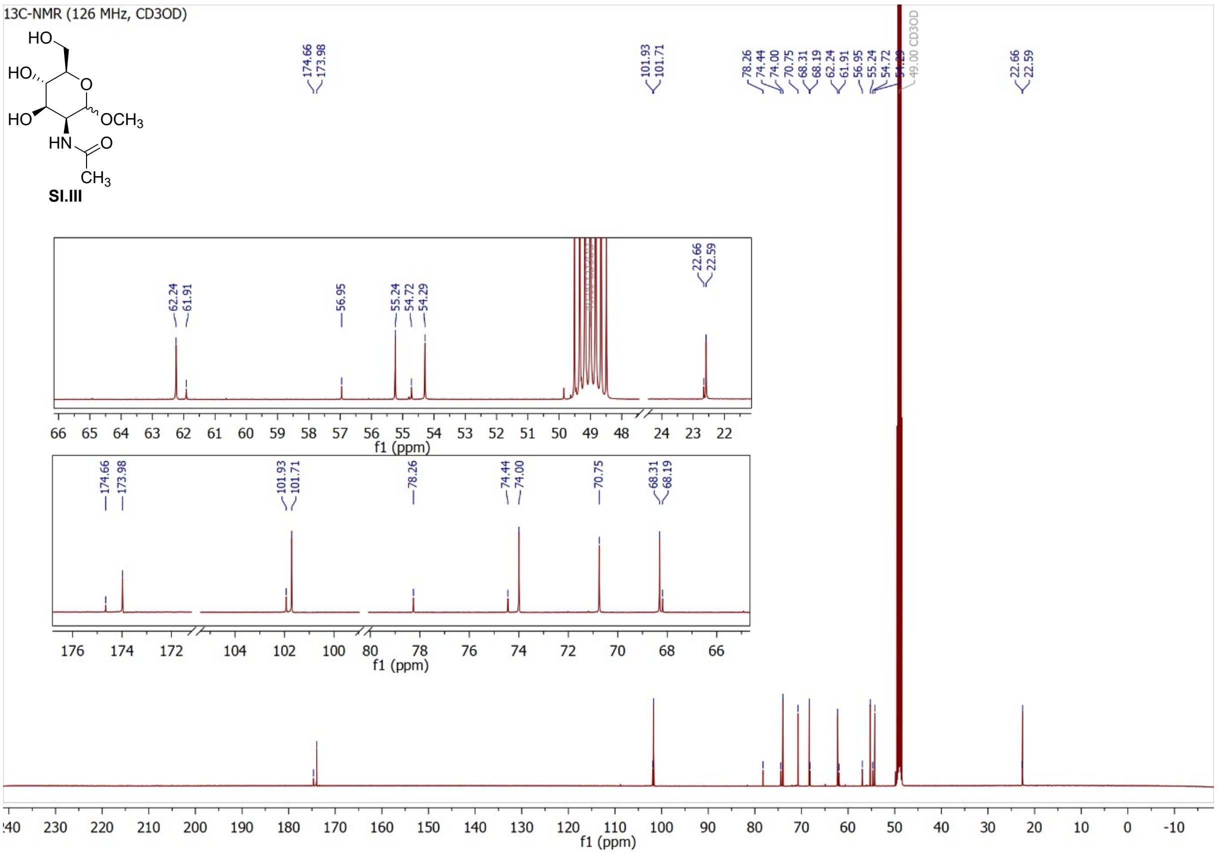

*Figure S14: ^{13}C{^{1}H}-NMR spectrum of minor anomer enriched fractions of product **SI.III**.*

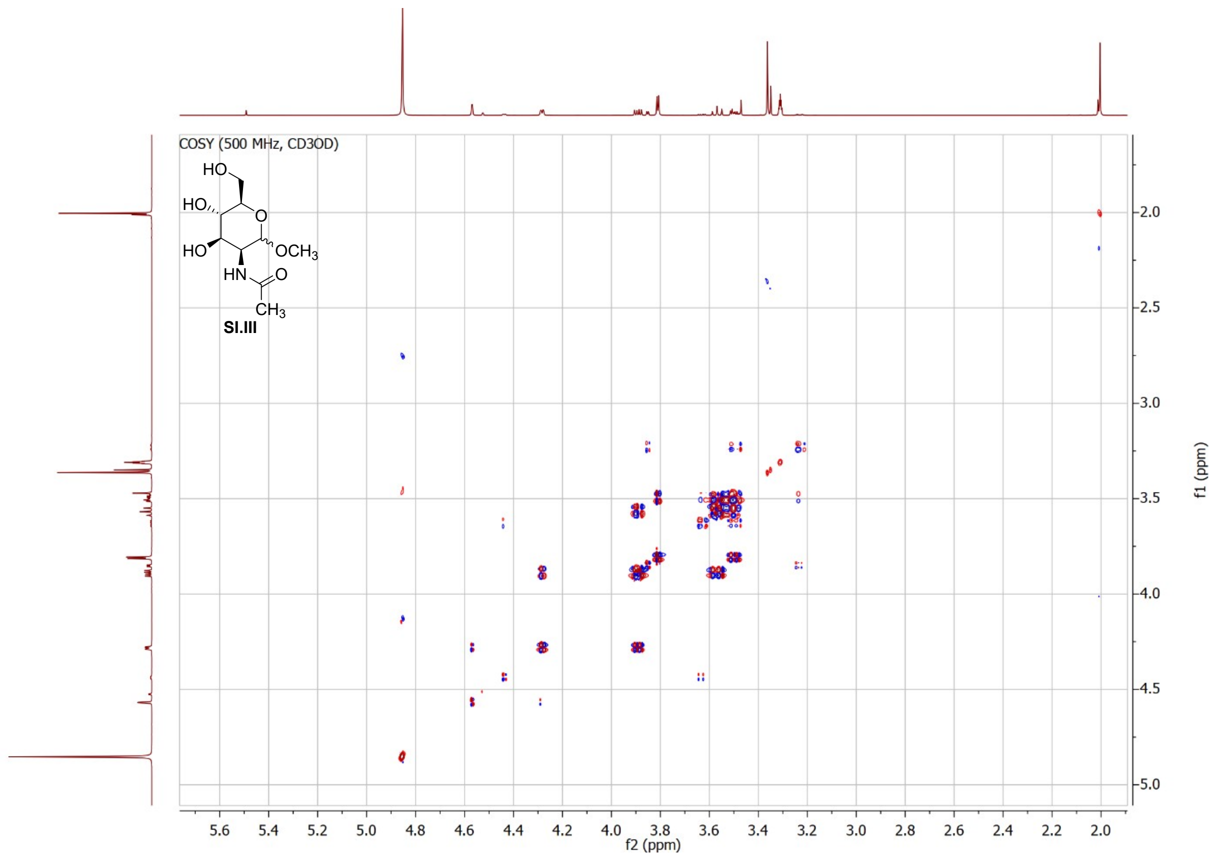

*Figure S15: COSY spectrum of minor anomer enriched fractions of product **SI.III**.*

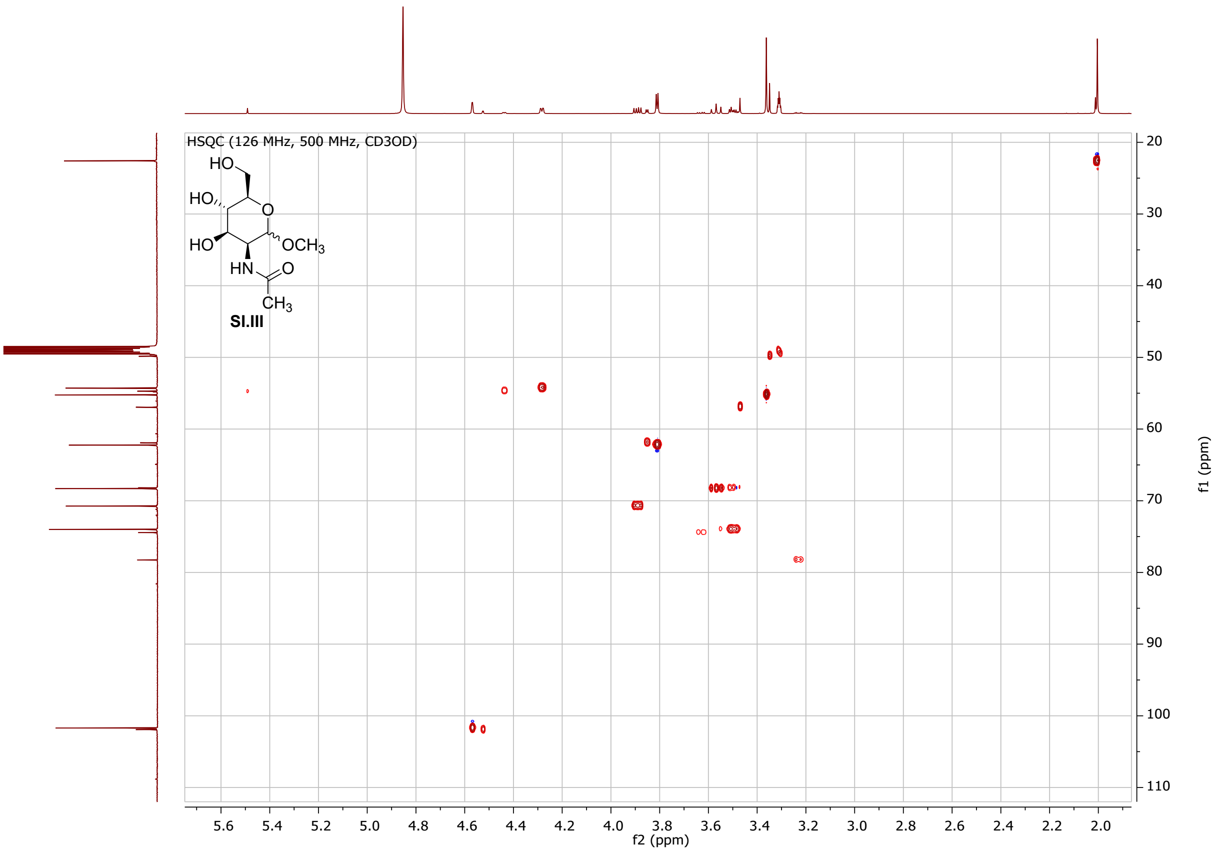

*Figure S16: HSQC spectrum of minor anomer enriched fractions of product **SI.III**.*

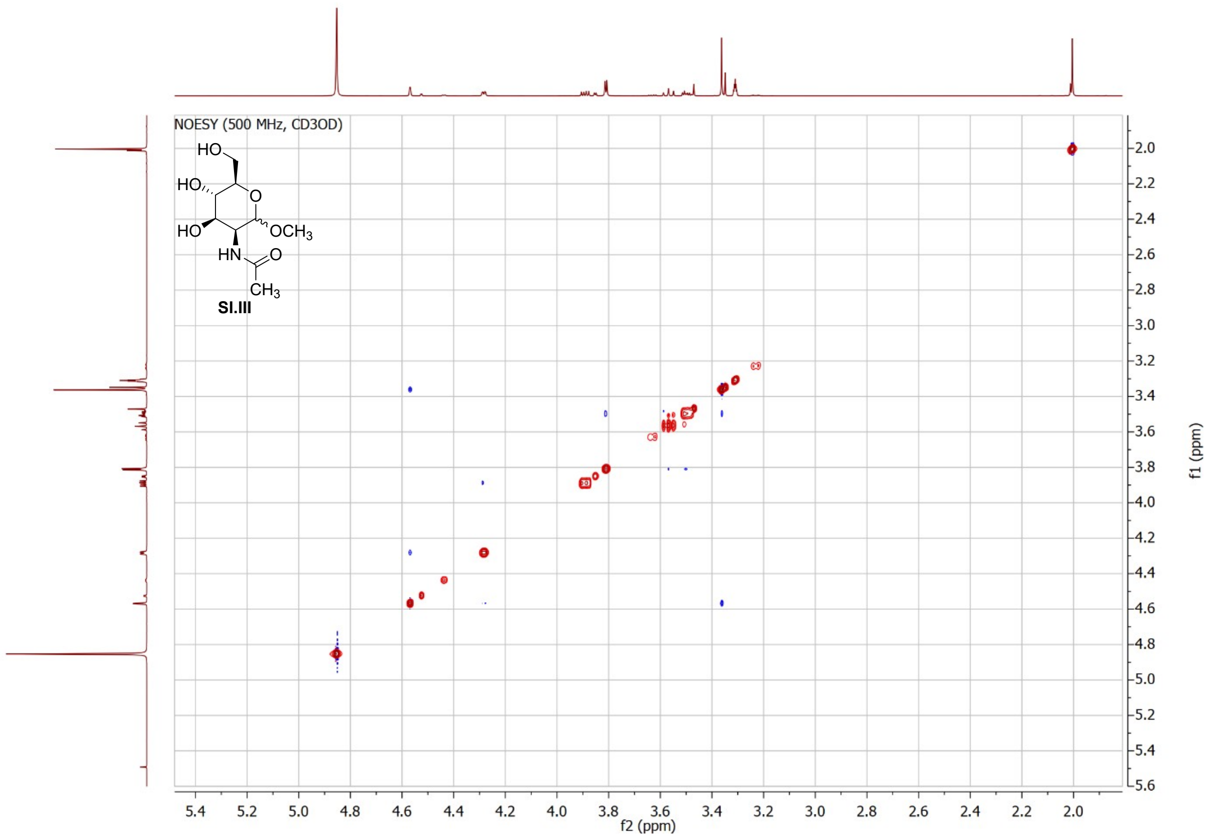

*Figure S17: NOESY spectrum of minor anomer enriched fractions of product **SI.III**.*

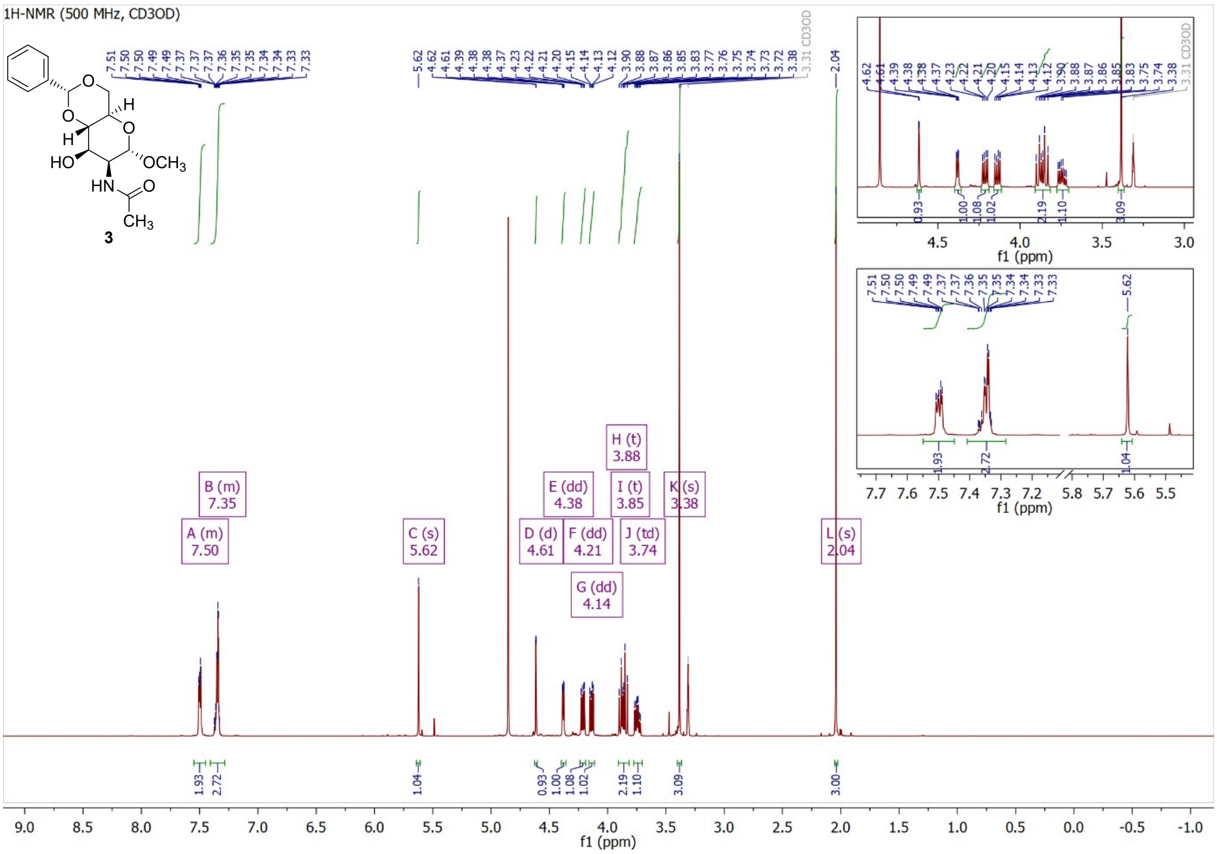

*Figure S18: ^{1}H-NMR spectrum of compound **3**.*

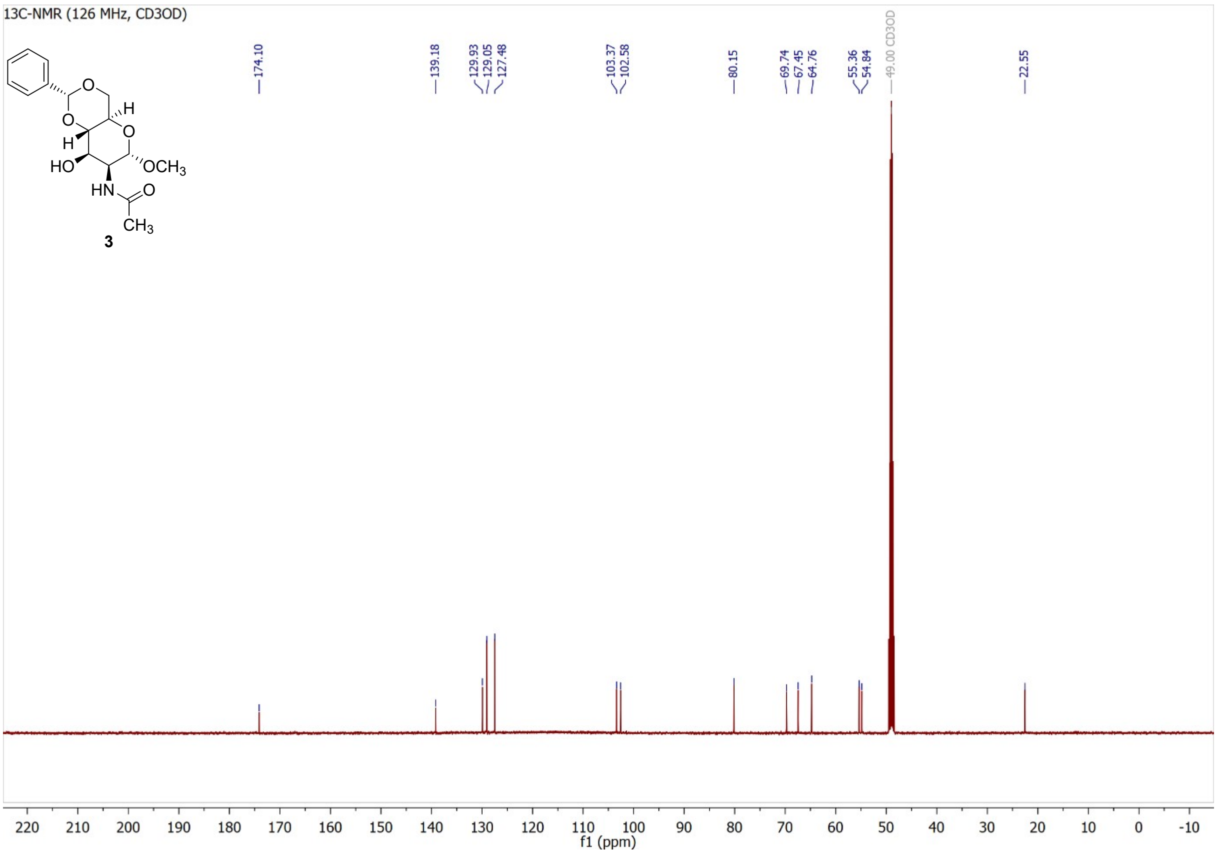

*Figure S19: $^{13}C\{^1H\}$-NMR spectrum of compound **3**.*

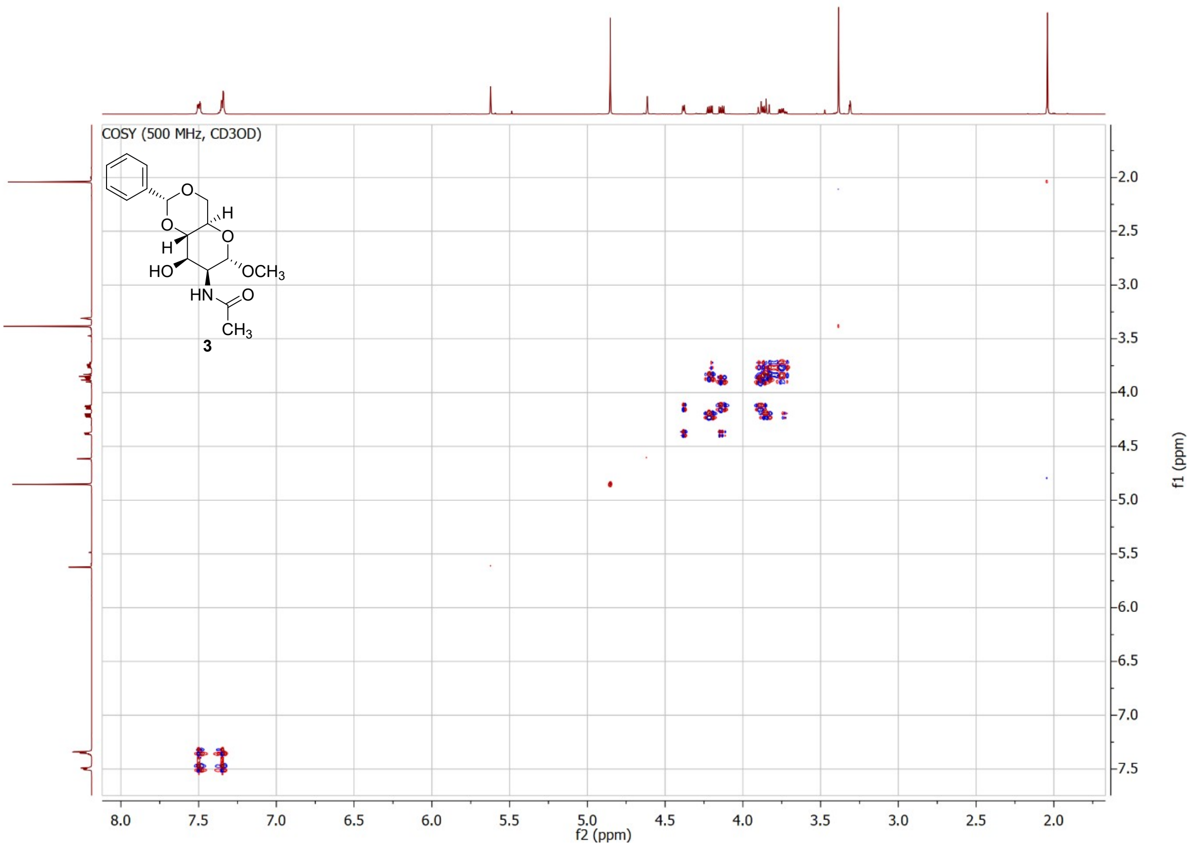

*Figure S20: COSY spectrum of compound **3**.*

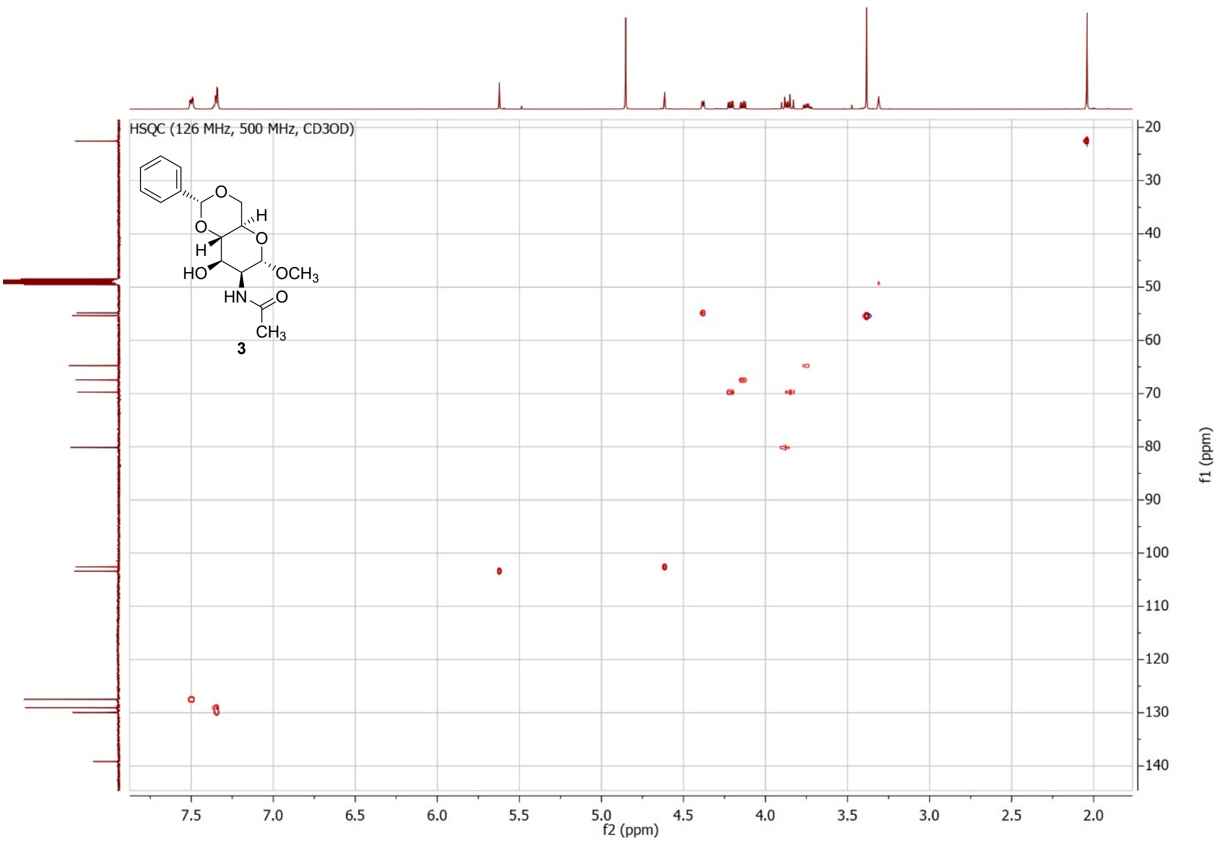

*Figure S21: HSQC spectrum of compound **3**.*

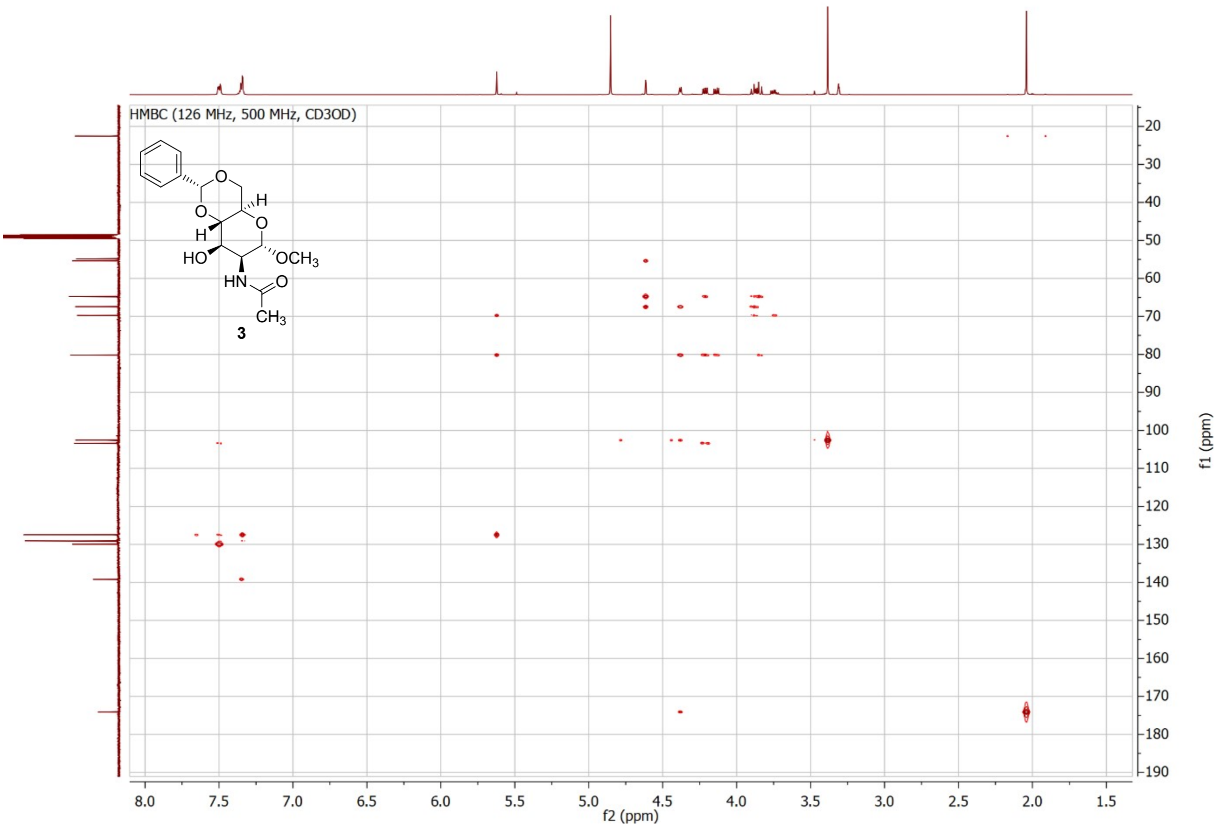

*Figure S22: HMBC spectrum of compound **3**.*

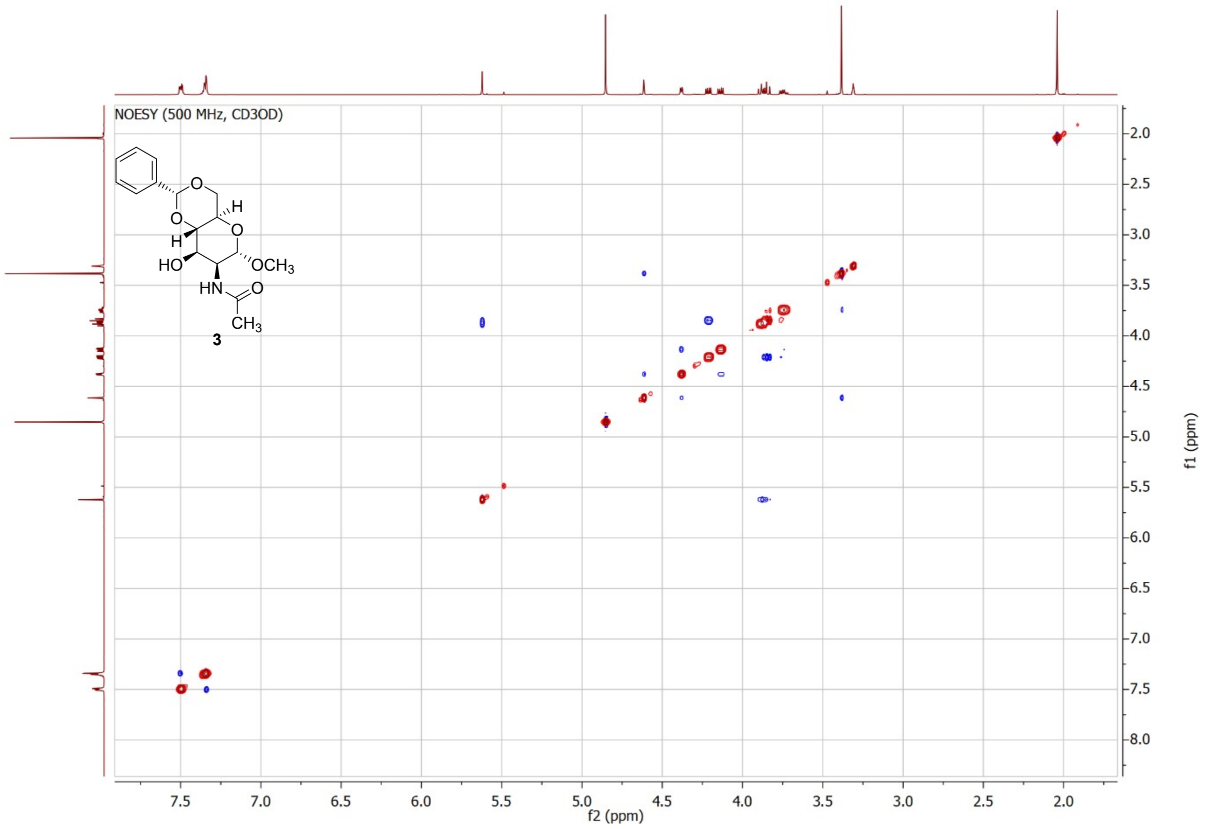

*Figure S23: NOESY spectrum of compound **3**.*

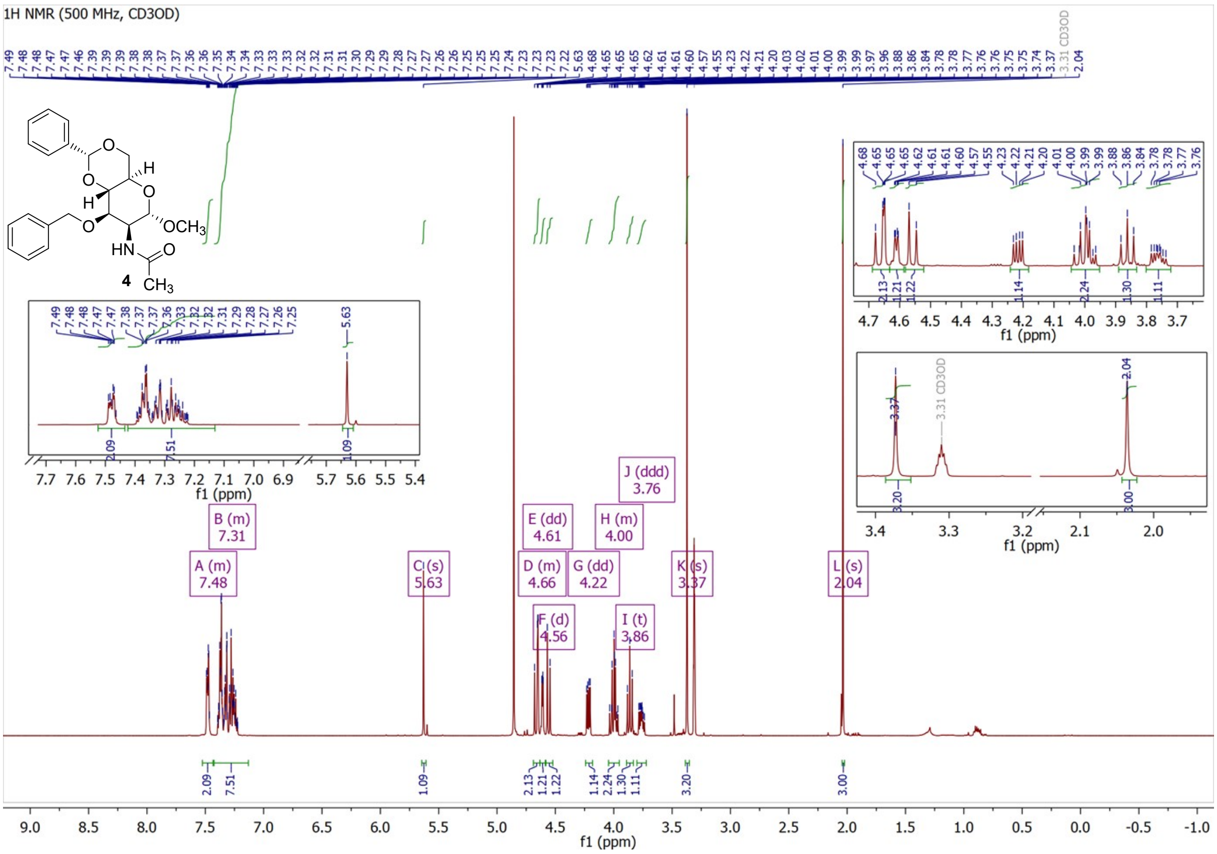

*Figure S24: ^{1}H-NMR spectrum of compound **4**.*

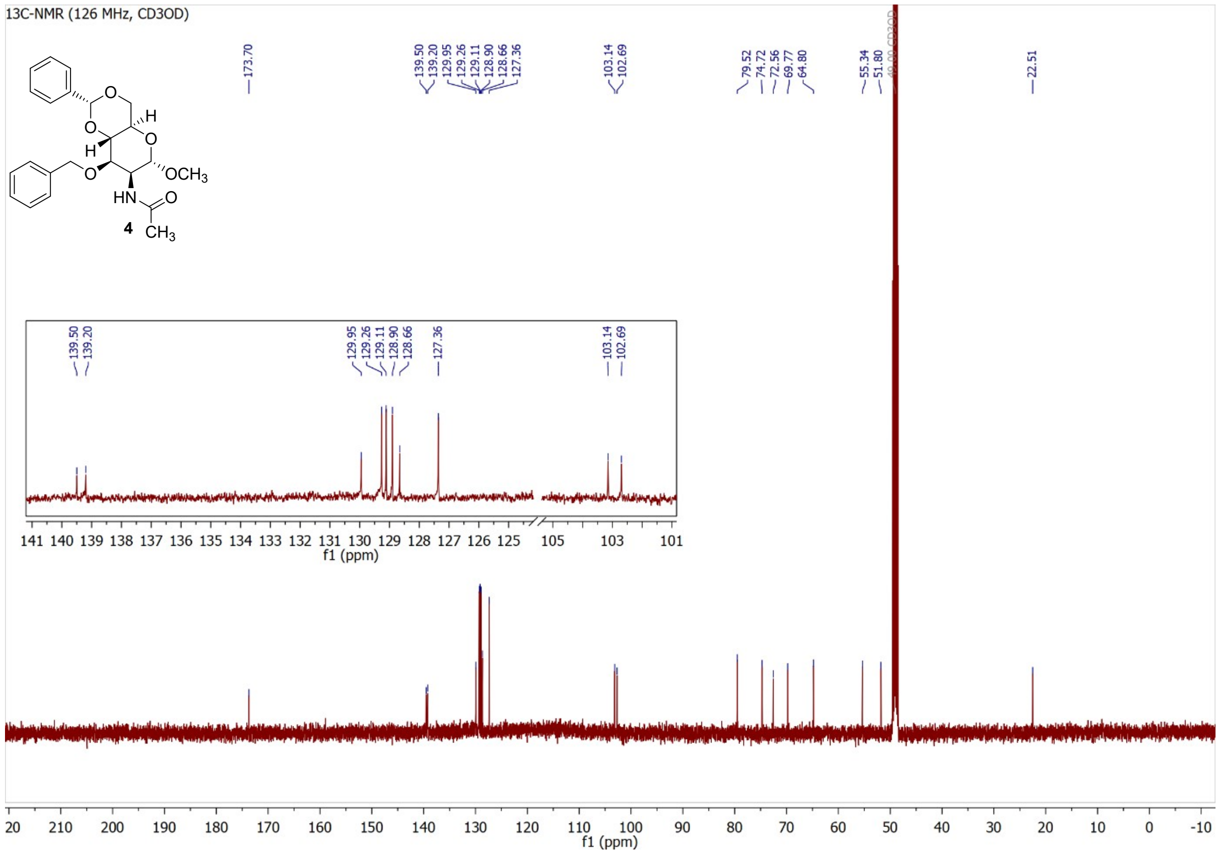

*Figure S25: $^{13}C\{^{1}H\}$-NMR spectrum of compound **4**.*

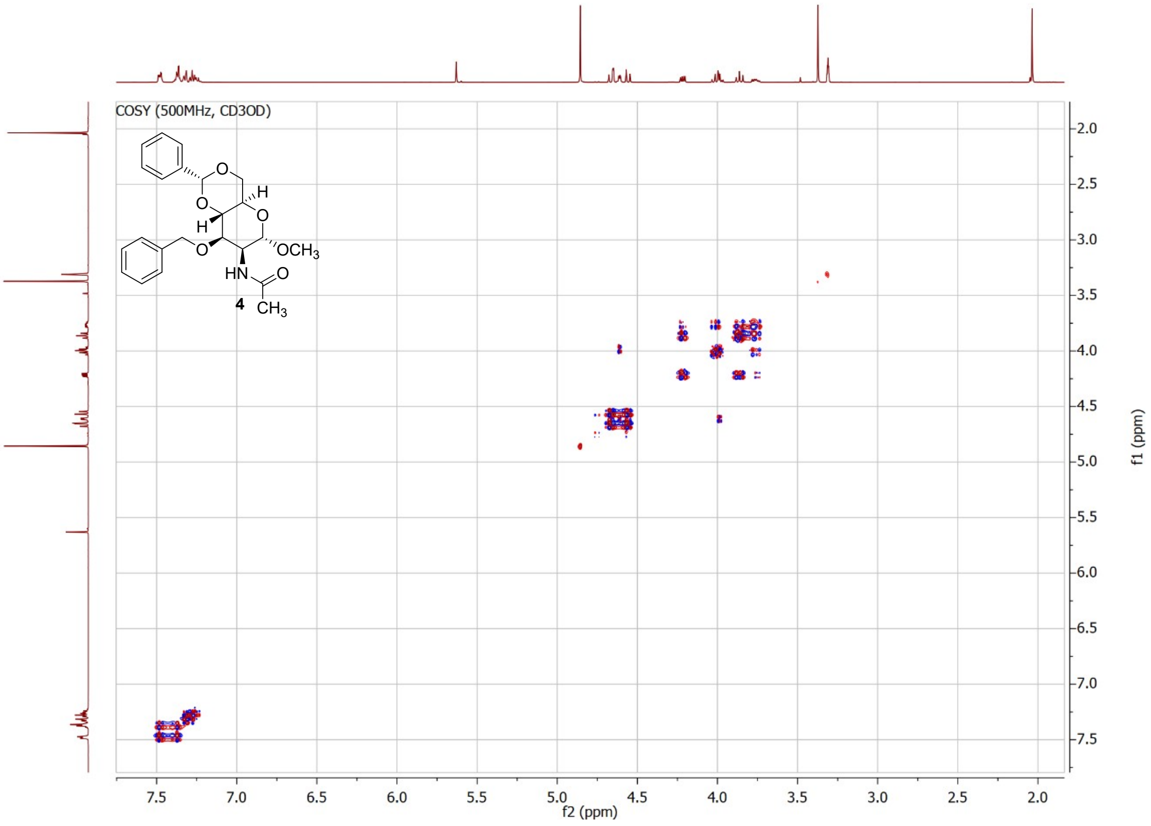

*Figure S26: COSY spectrum of compound **4**.*

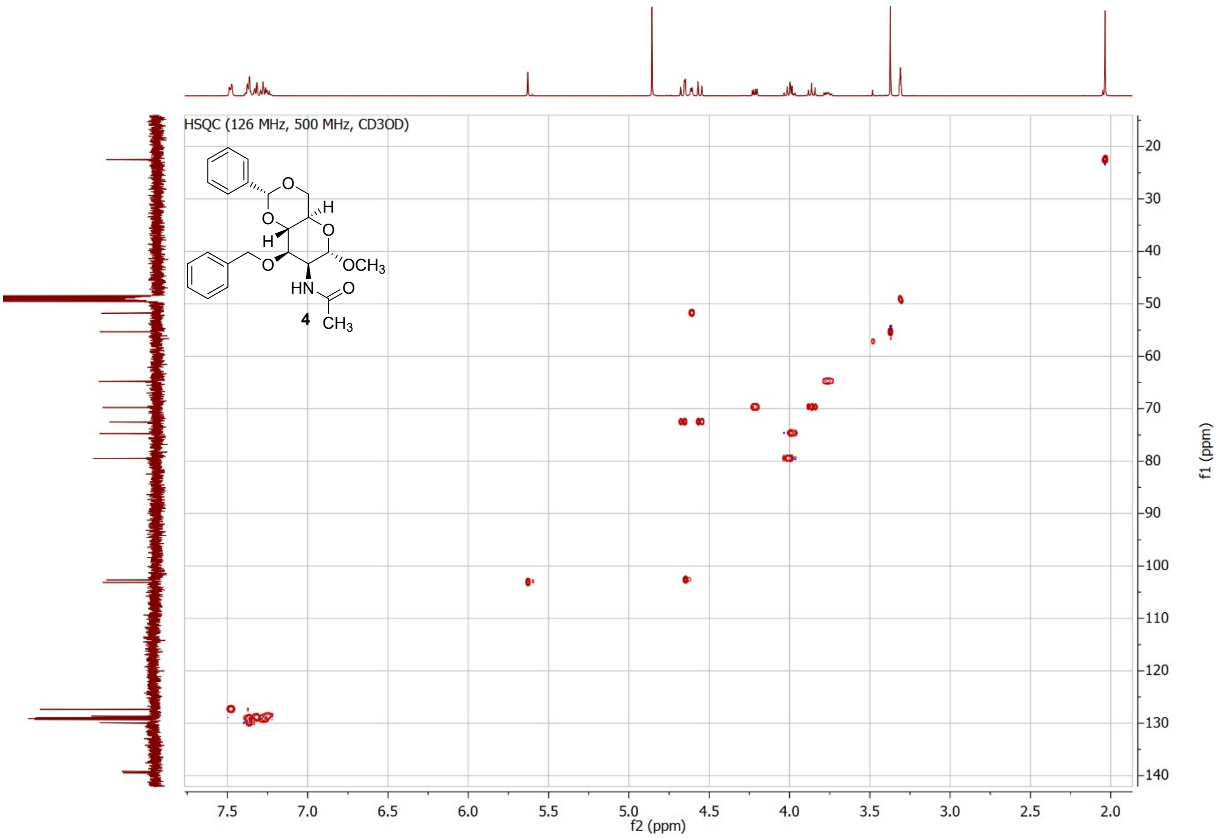

*Figure S27: HSQC spectrum of compound **4**.*

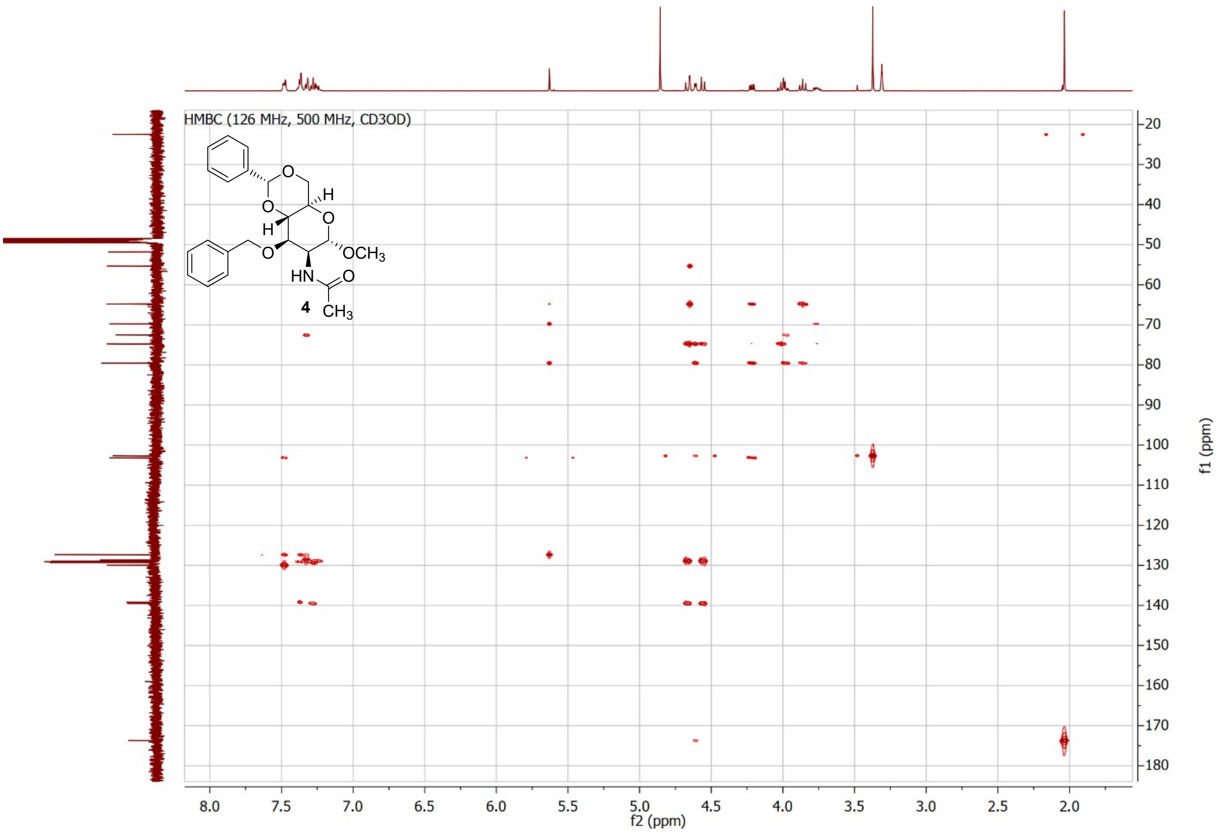

*Figure S28: HMBC spectrum of compound **4**.*

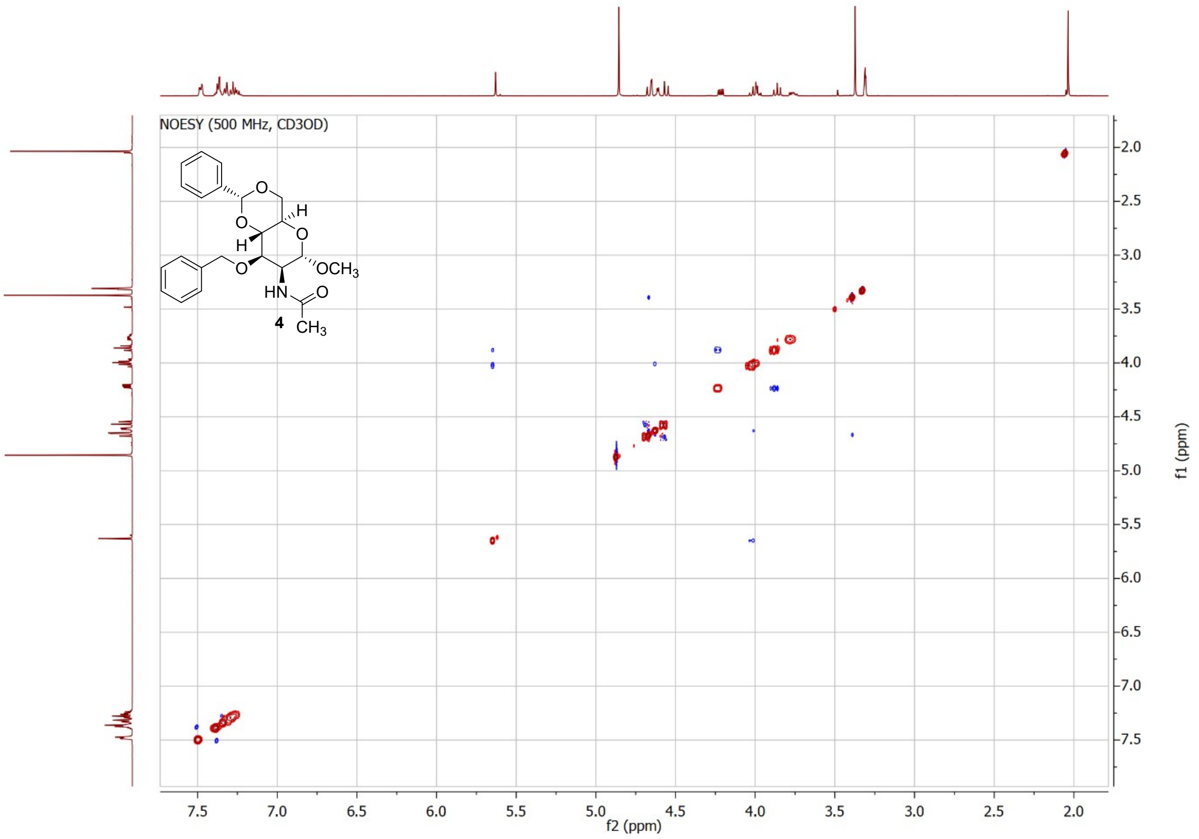

*Figure S29: NOESY spectrum of compound **4**.*

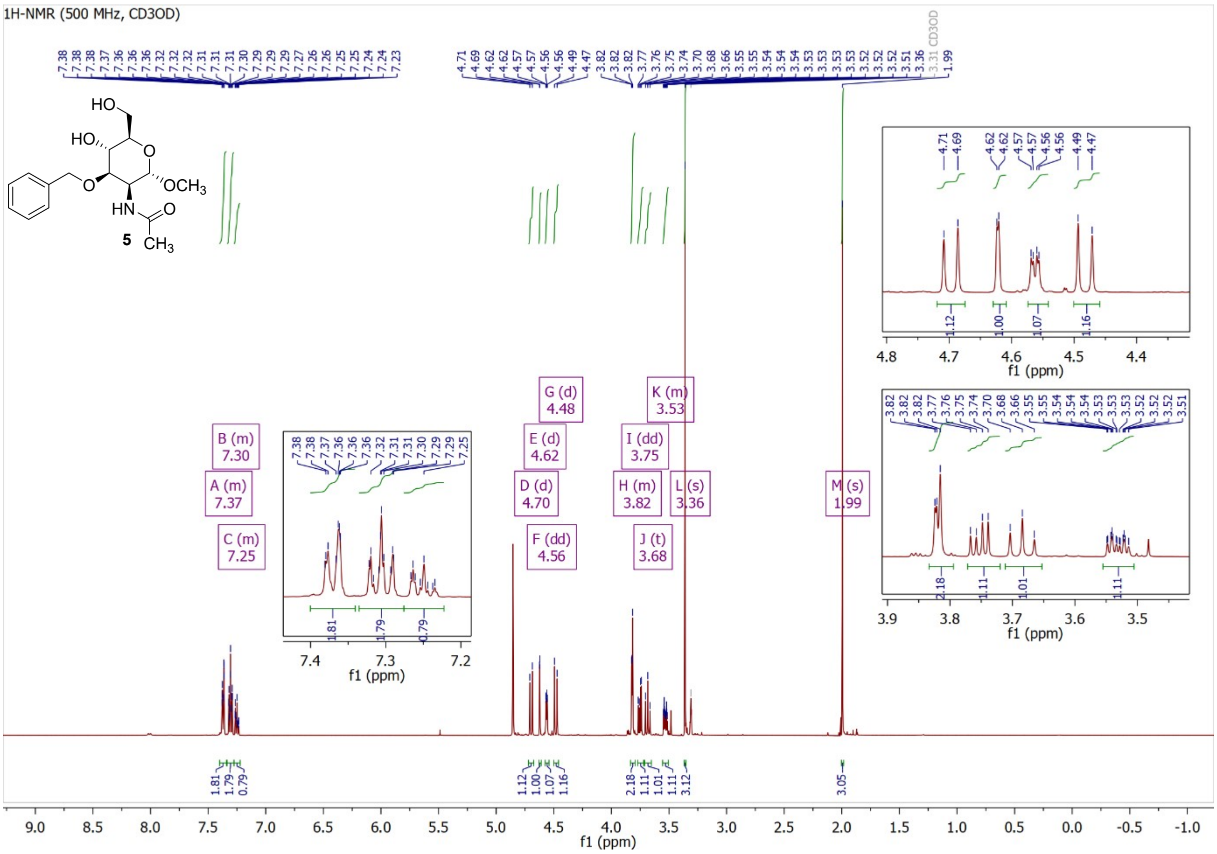

*Figure S30: ^{1}H-NMR spectrum of compound **5**.*

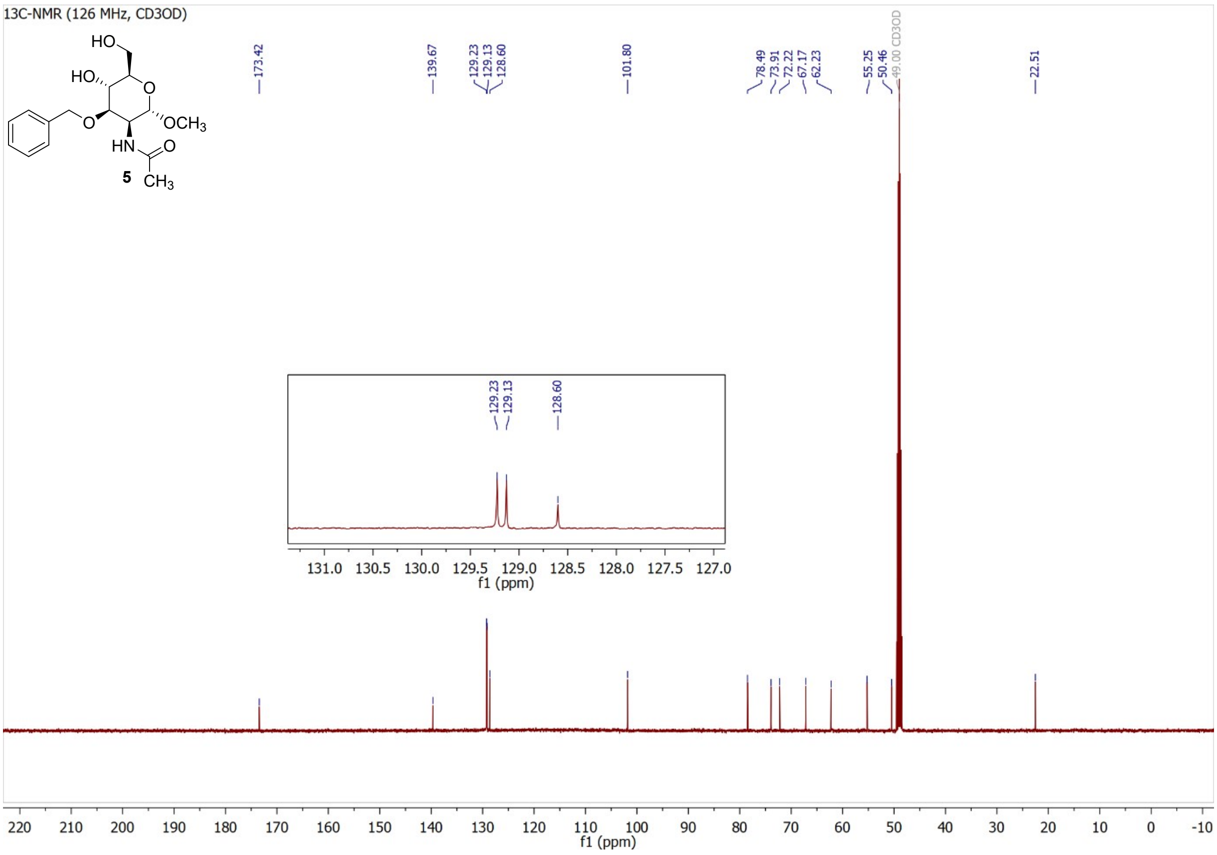

*Figure S31: $^{13}C\{^{1}H\}$-NMR spectrum of compound **5**.*

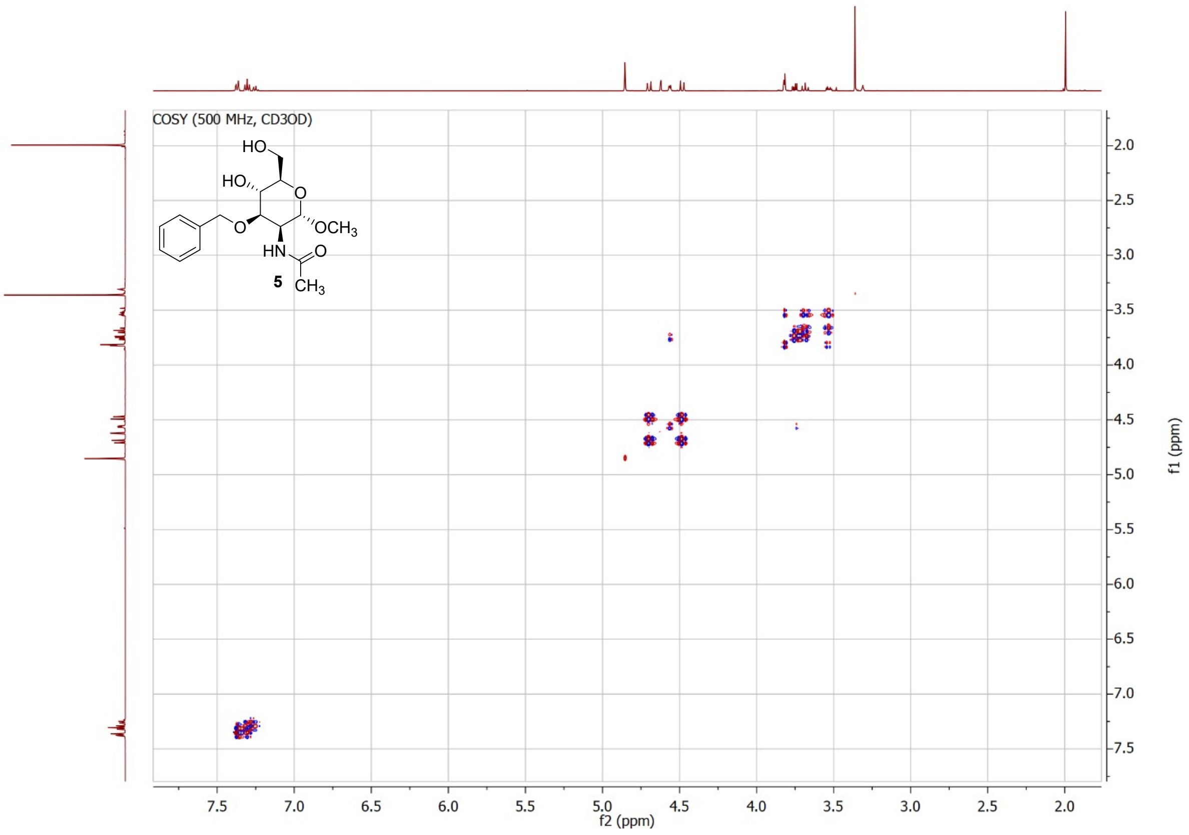

*Figure S32: COSY spectrum of compound **5**.*

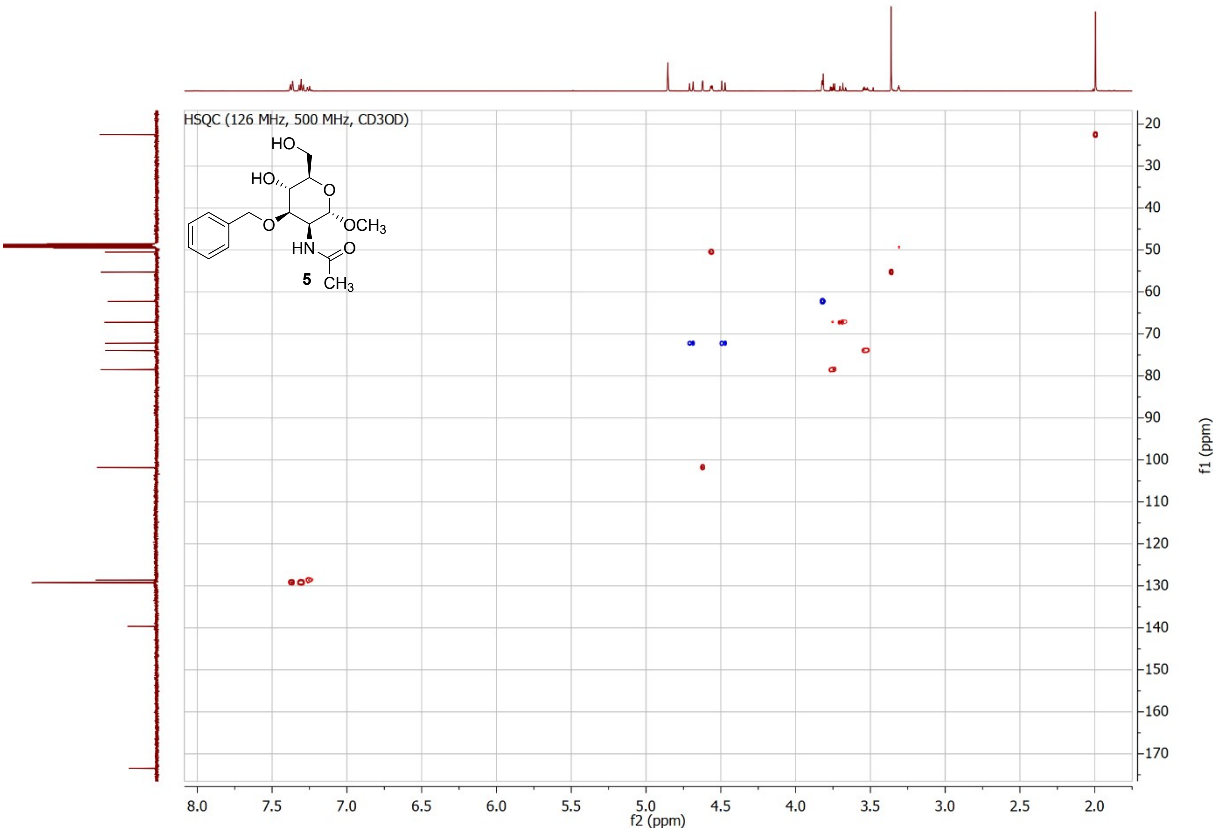

*Figure S33: HSQC spectrum of compound **5**.*

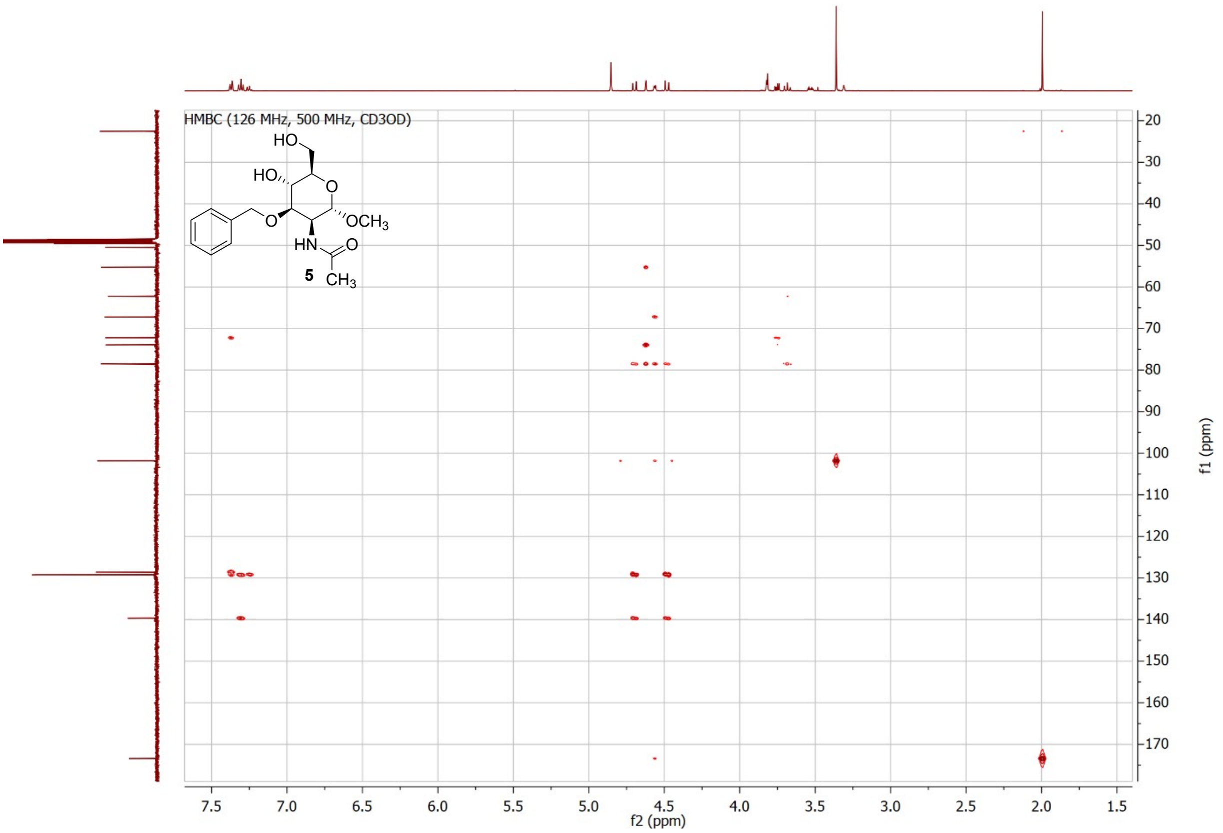

*Figure S34: HMBC spectrum of compound **5**.*

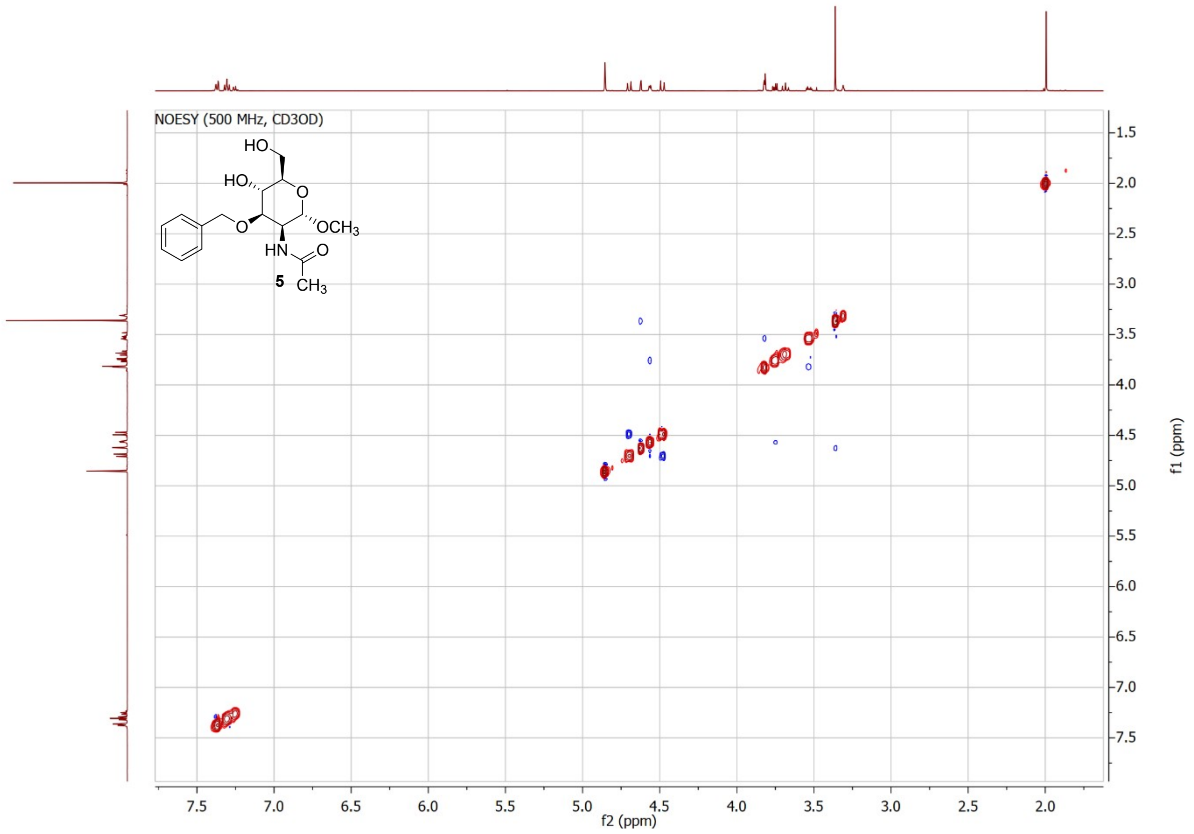

Figure S35: NOESY spectrum of compound **5**.

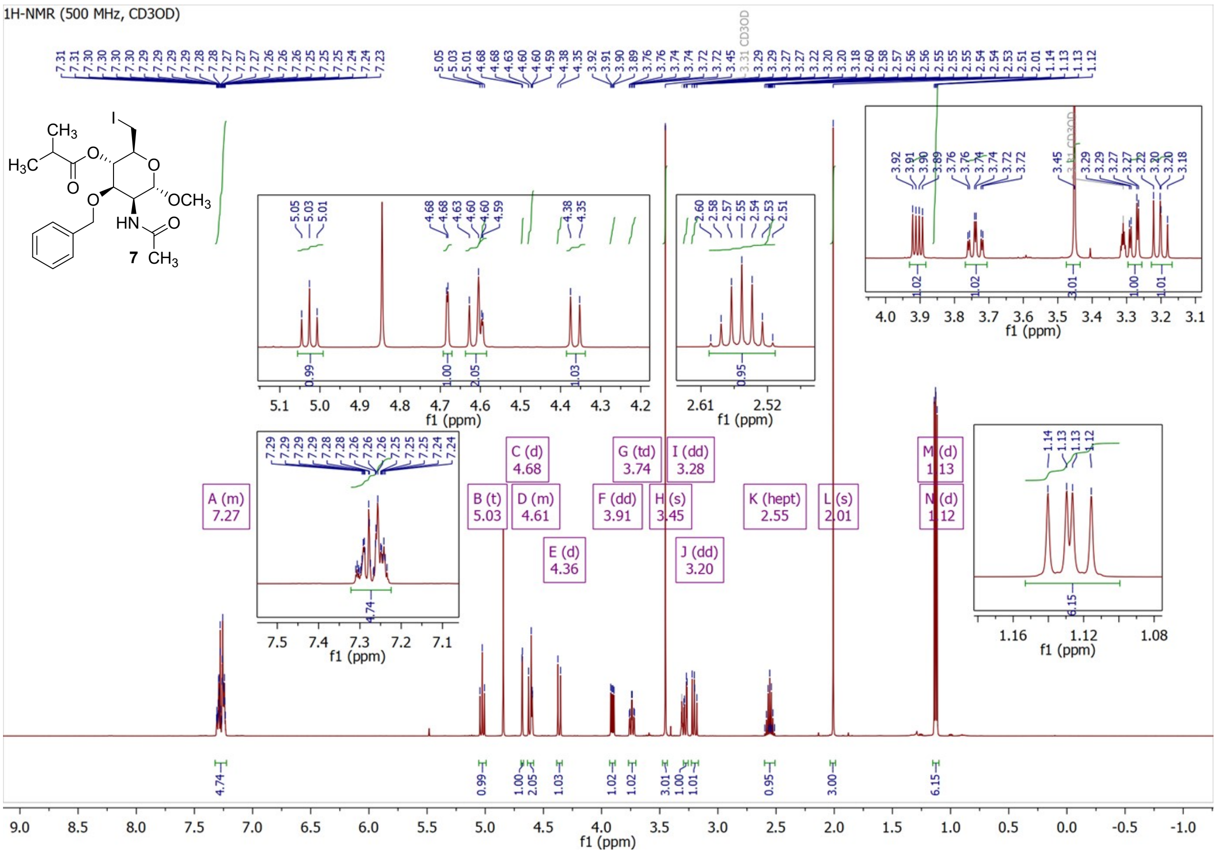

Figure S36: ^{1}H-NMR spectrum of compound **7**.

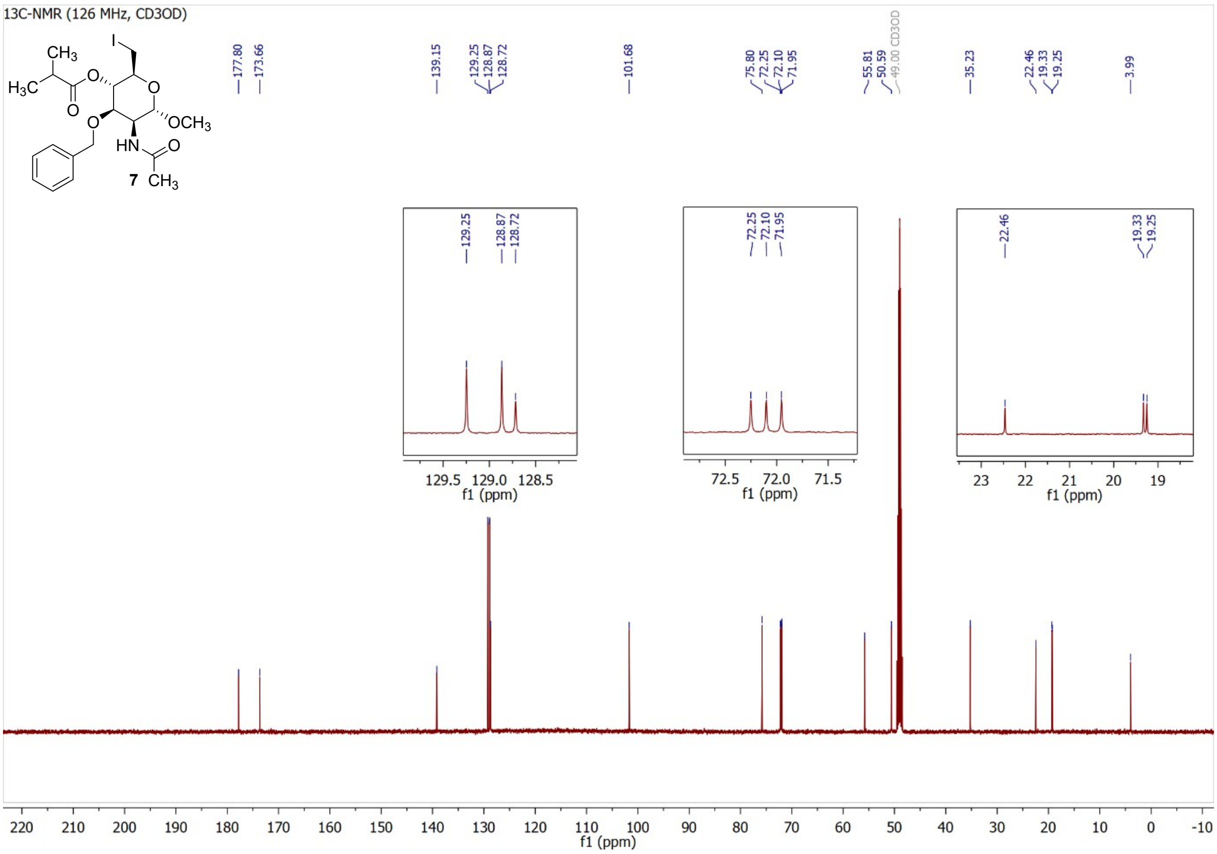

*Figure S37: $^{13}C\{^{1}H\}$-NMR spectrum of compound **7**.*

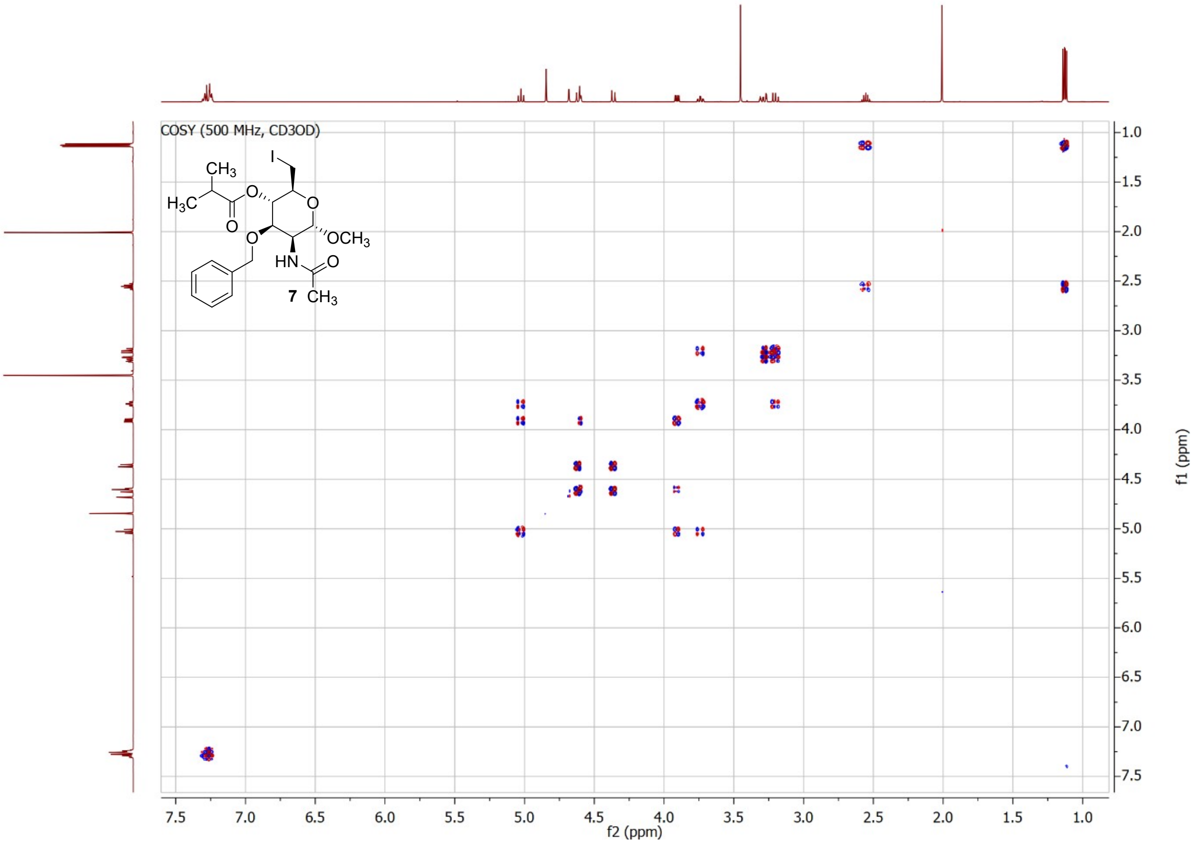

*Figure S38: COSY spectrum of compound **7**.*

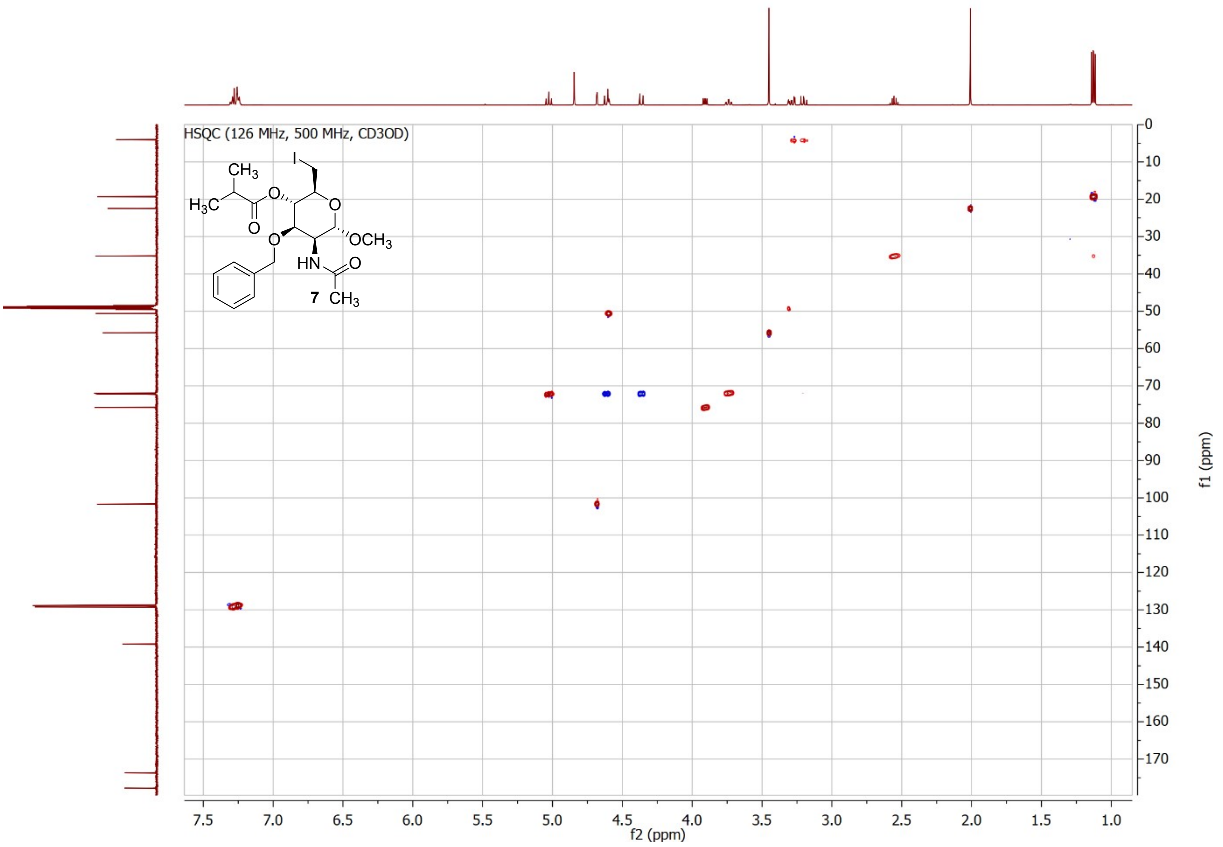

Figure S39: HSQC spectrum of compound ***7****.*

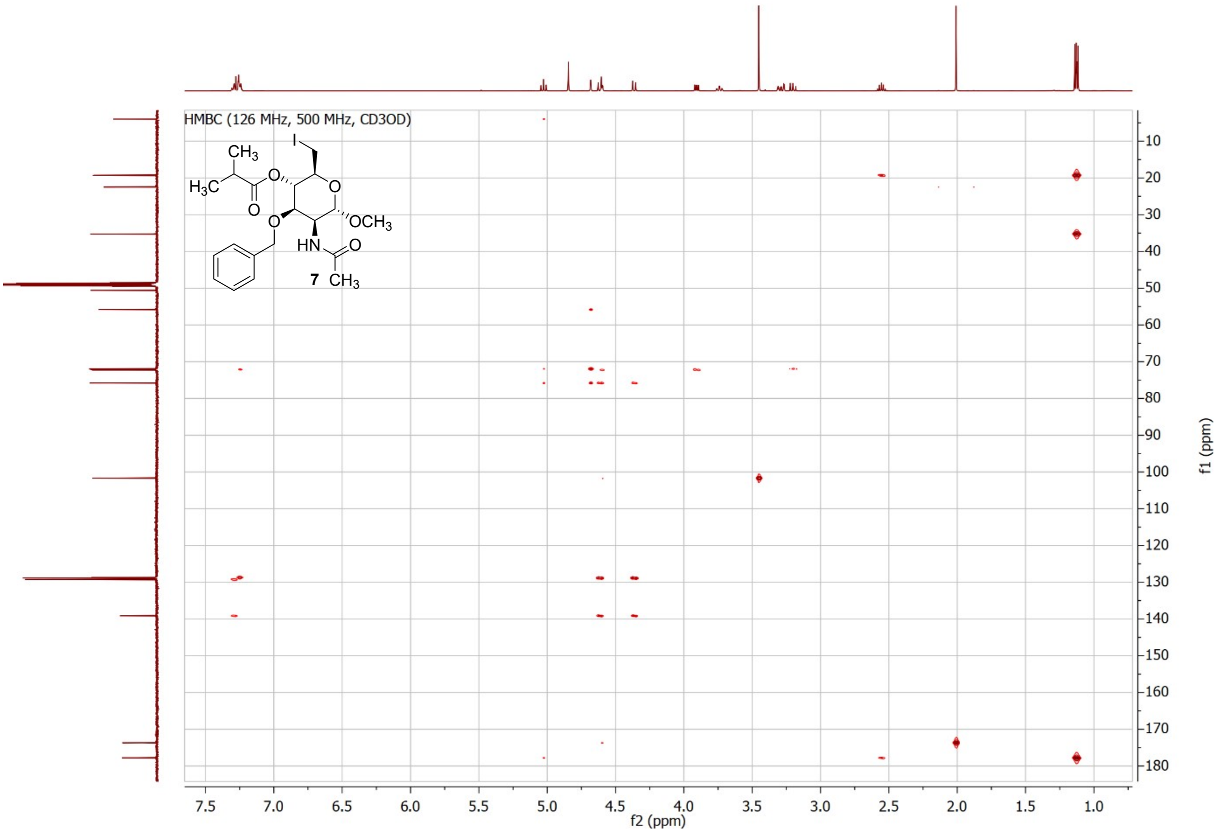

Figure S40: HMBC spectrum of compound ***7****.*

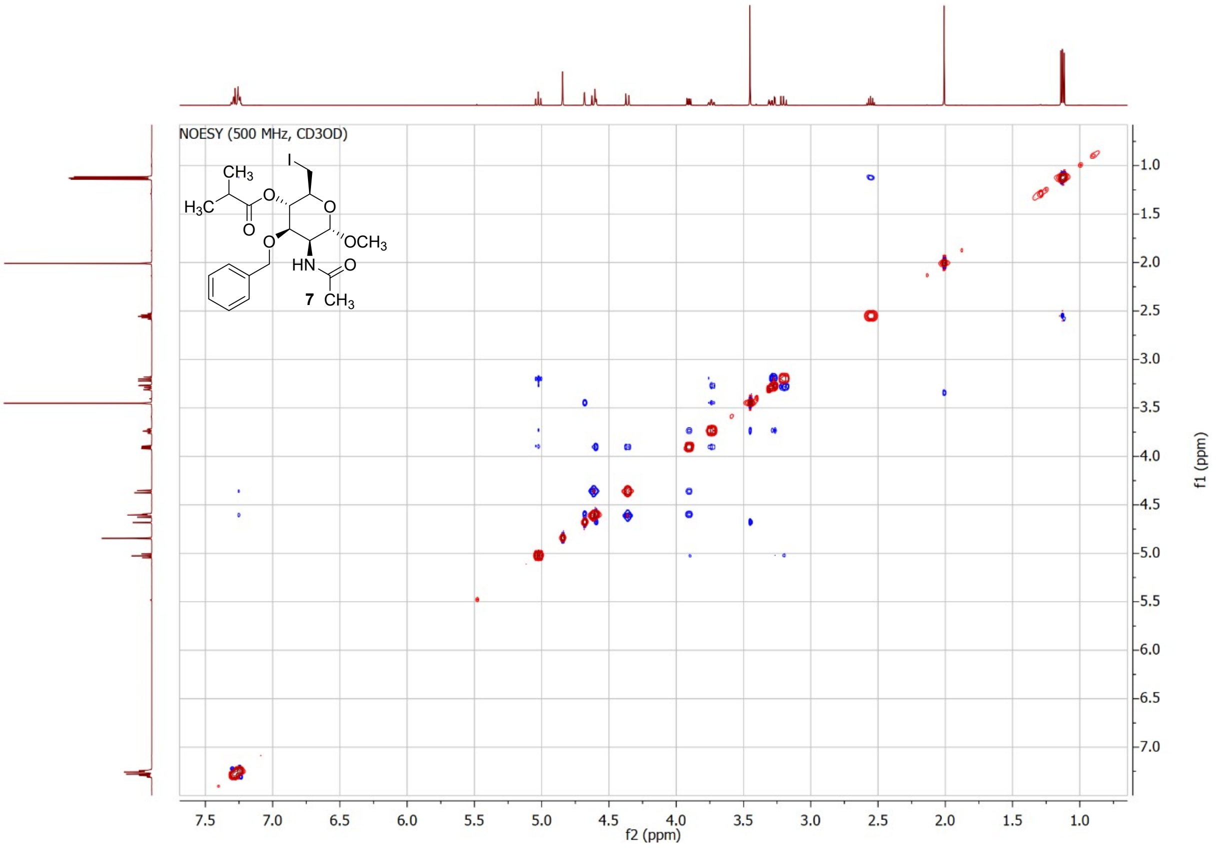

Figure S41: NOESY spectrum of compound ***7****.*

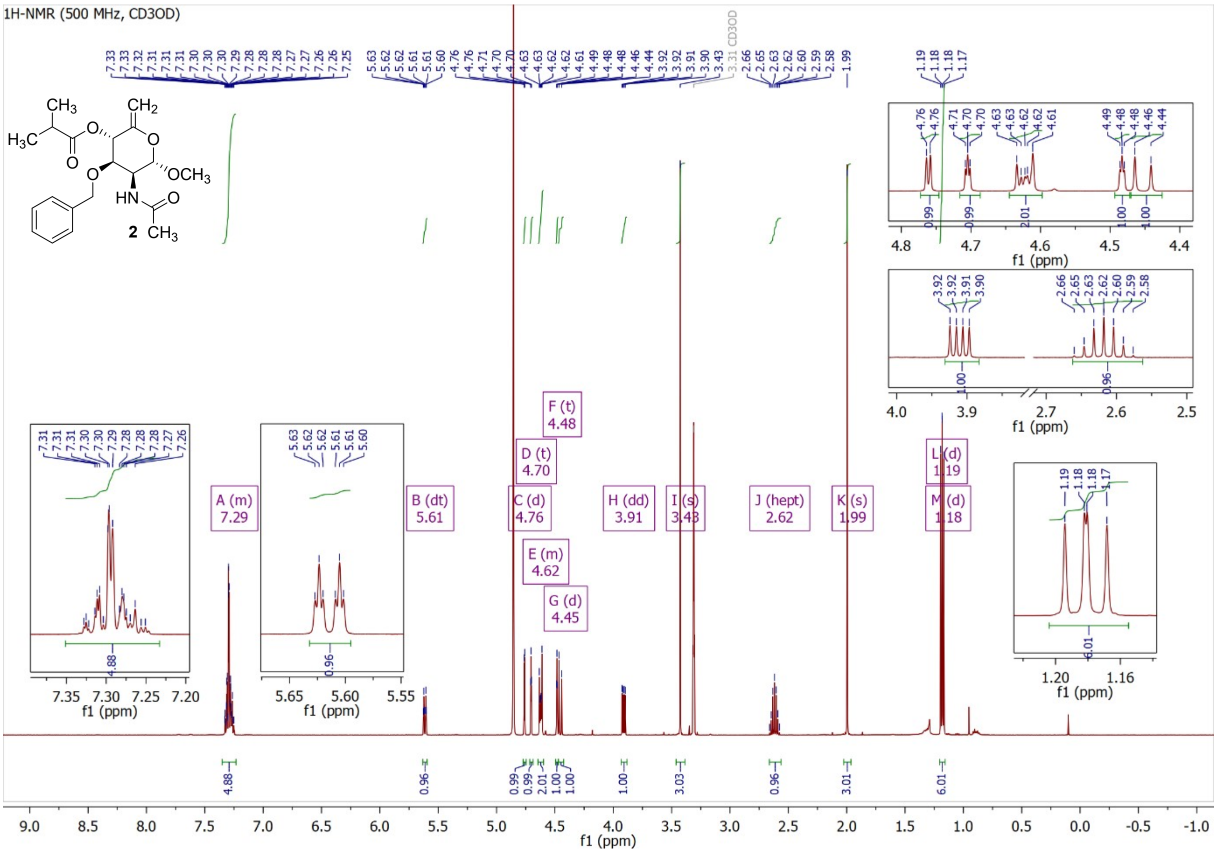

Figure S42: ¹H-NMR spectrum of compound ***2****.*

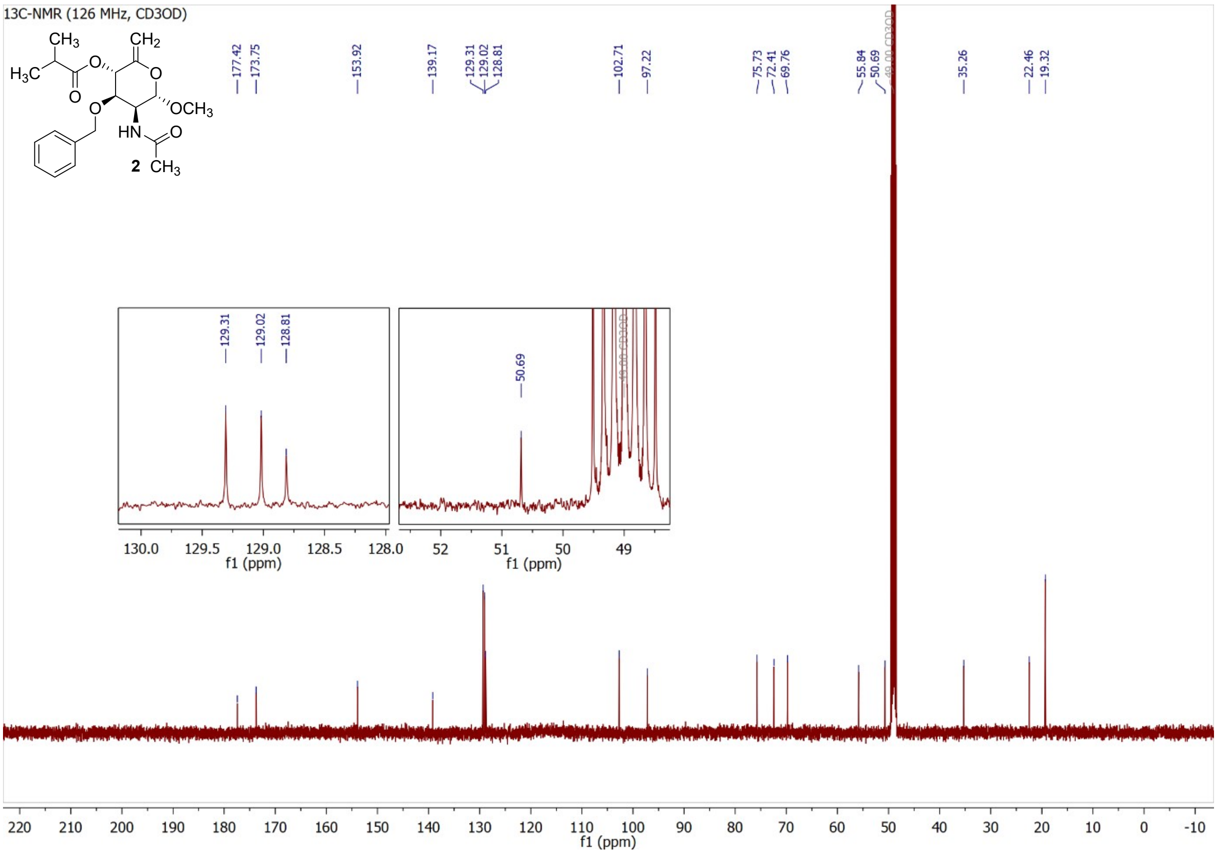

Figure S43: $^{13}C\{^1H\}$-NMR spectrum of compound**2**.

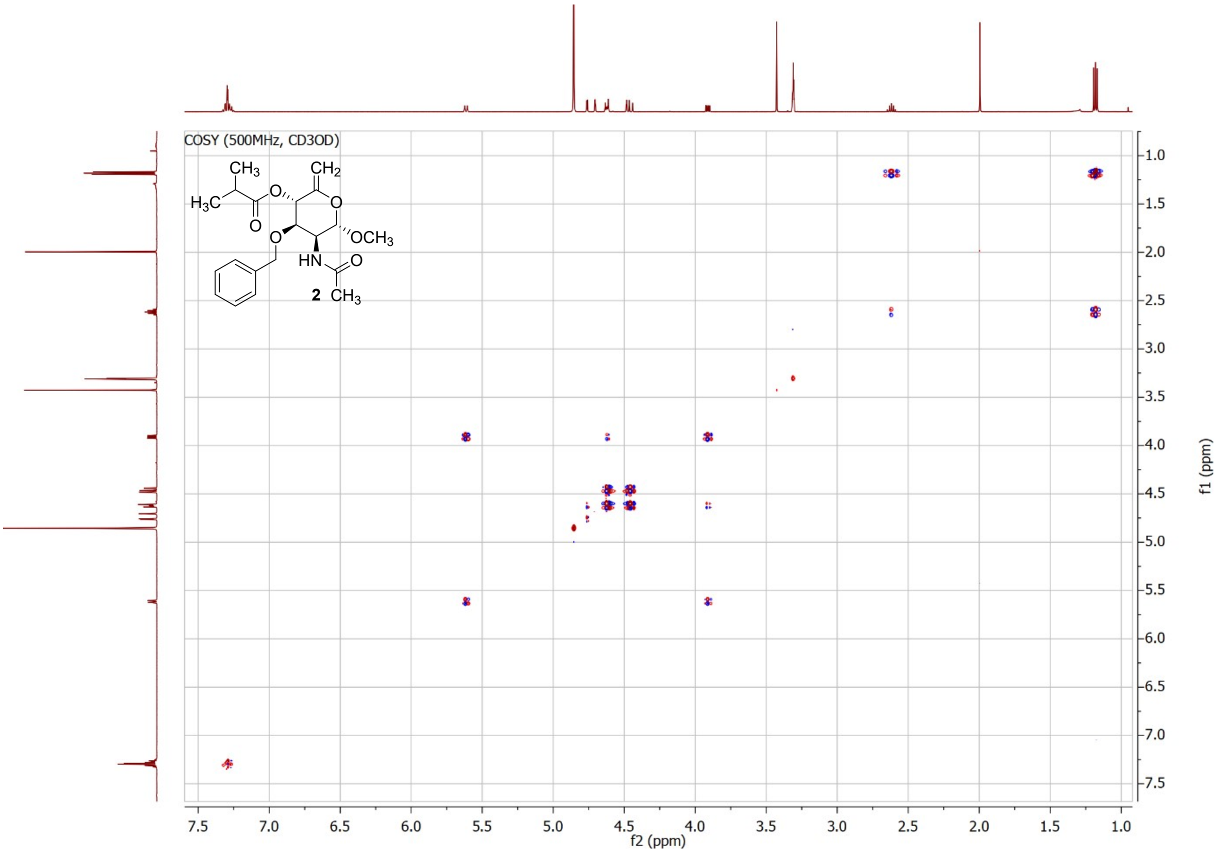

Figure S44: COSY spectrum of compound **2**.

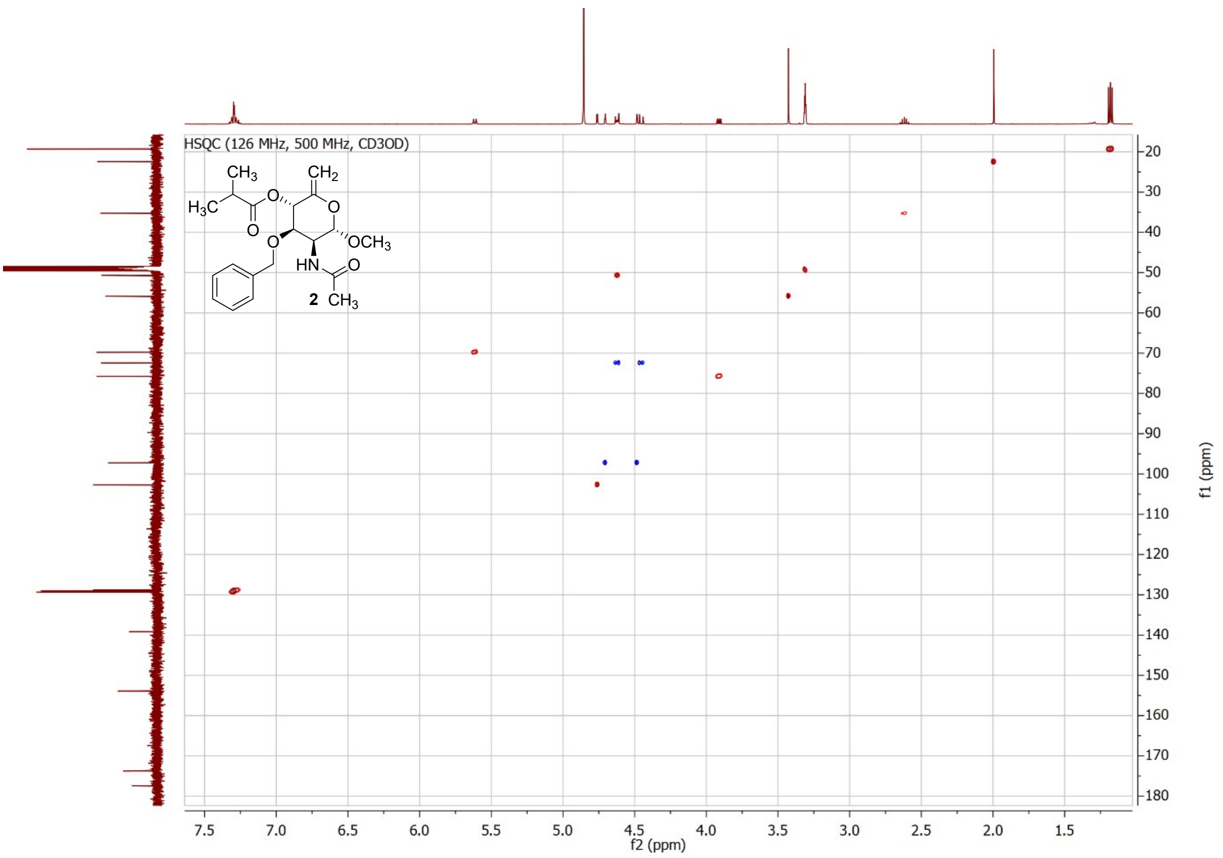

*Figure S45: HSQC spectrum of compound **2**.*

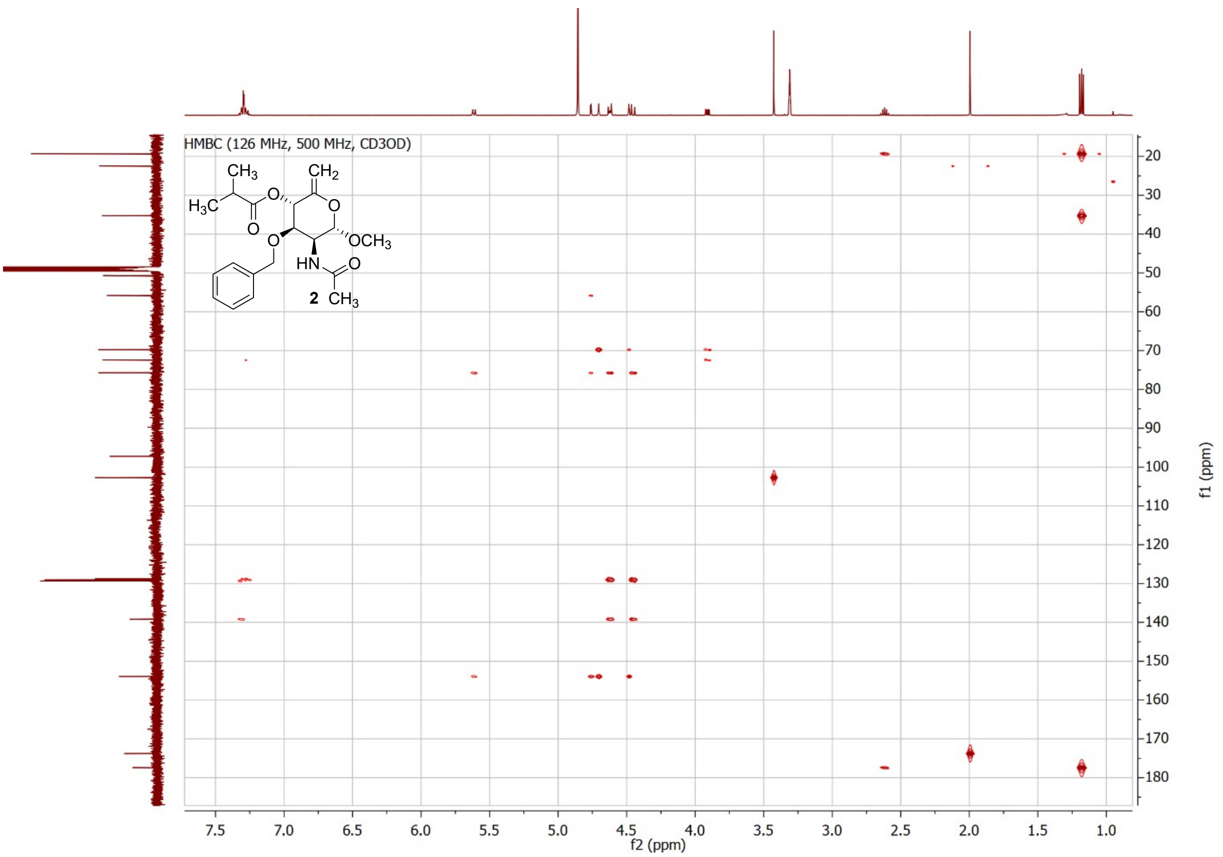

*Figure S46: HMBC spectrum of compound **2**.*

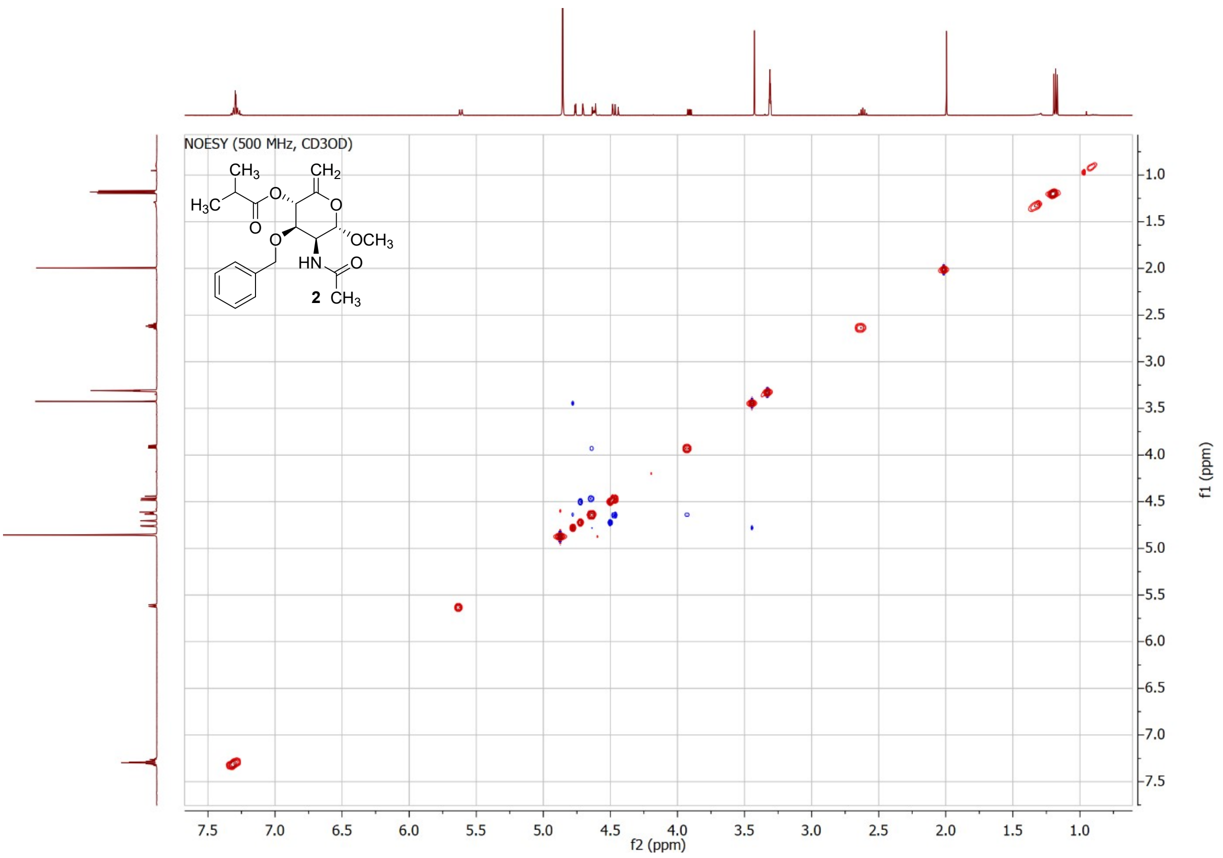

Figure S47: NOESY spectrum of compound ***2****.*

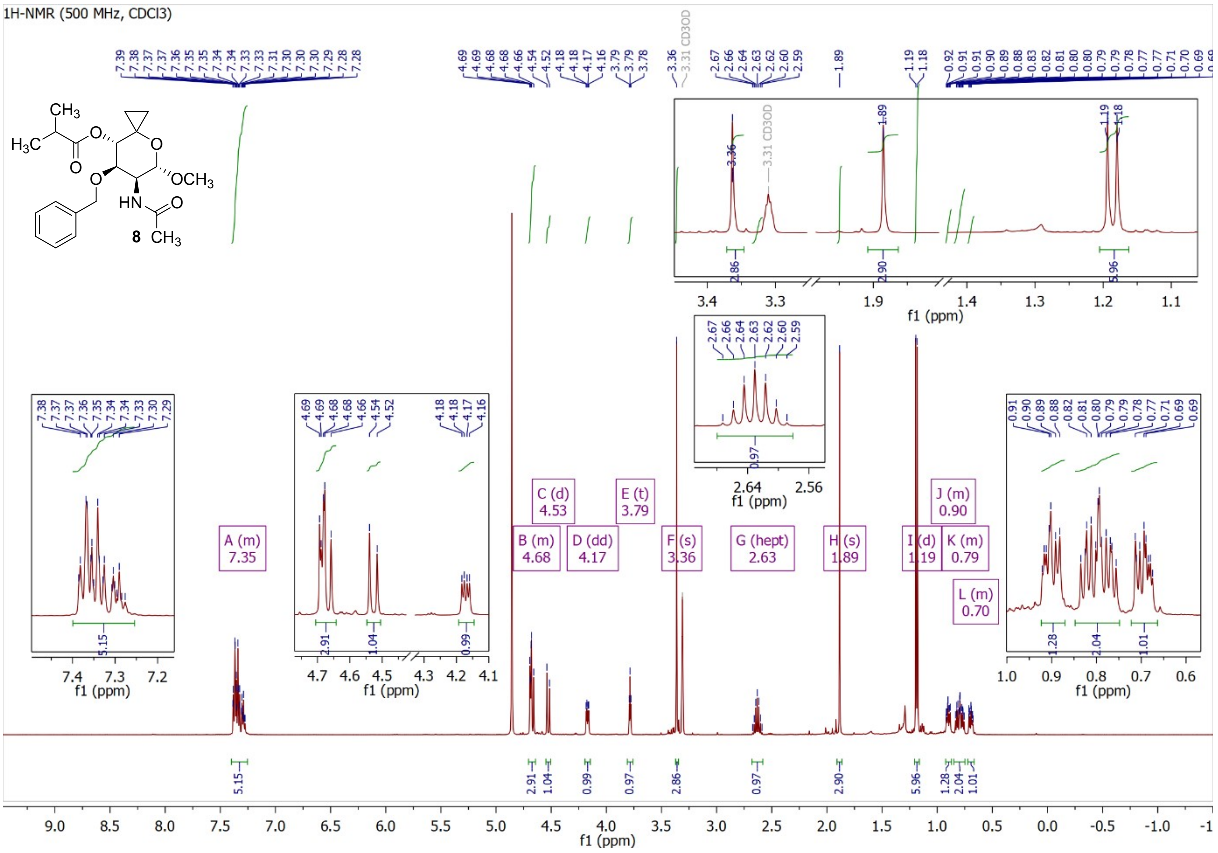

Figure S48: [1]*H-NMR spectrum of compound* ***8****.*

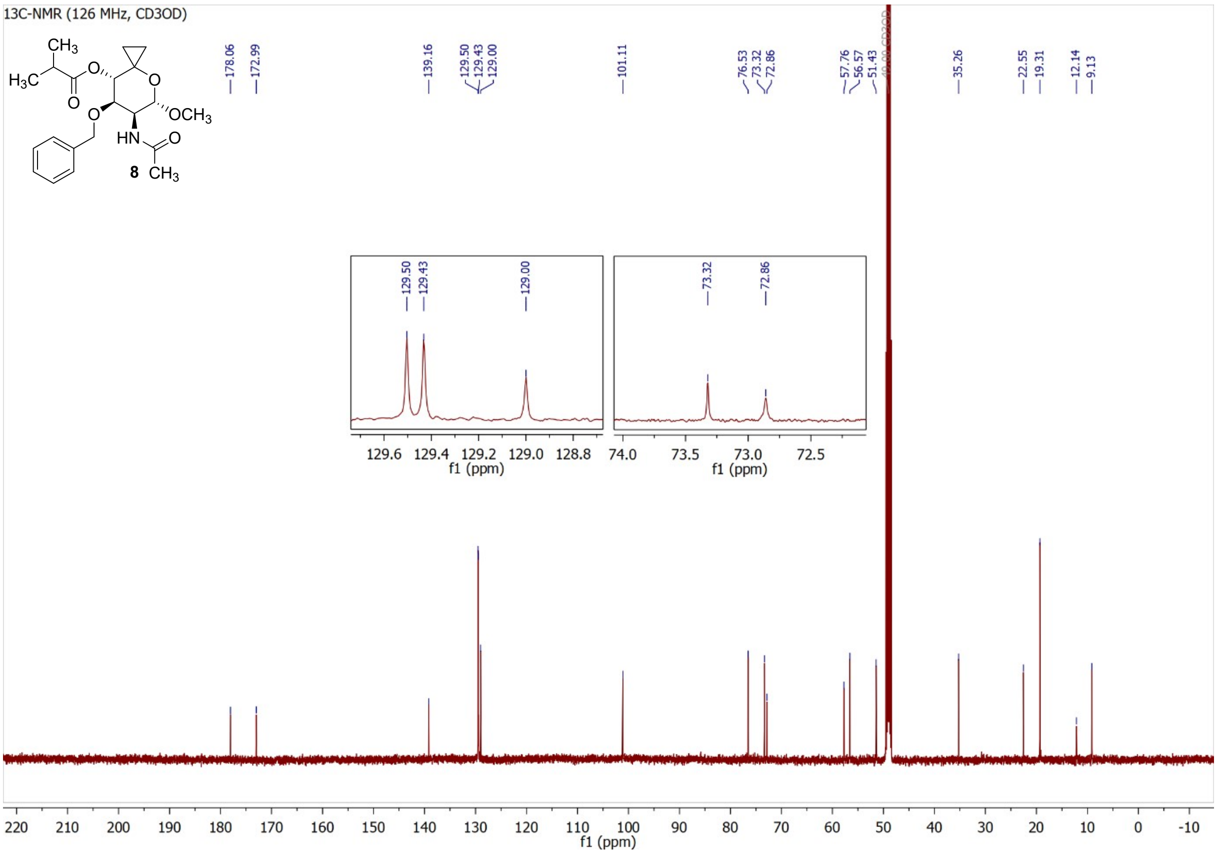

*Figure S49: $^{13}C\{^{1}H\}$-NMR spectrum of compound **8**.*

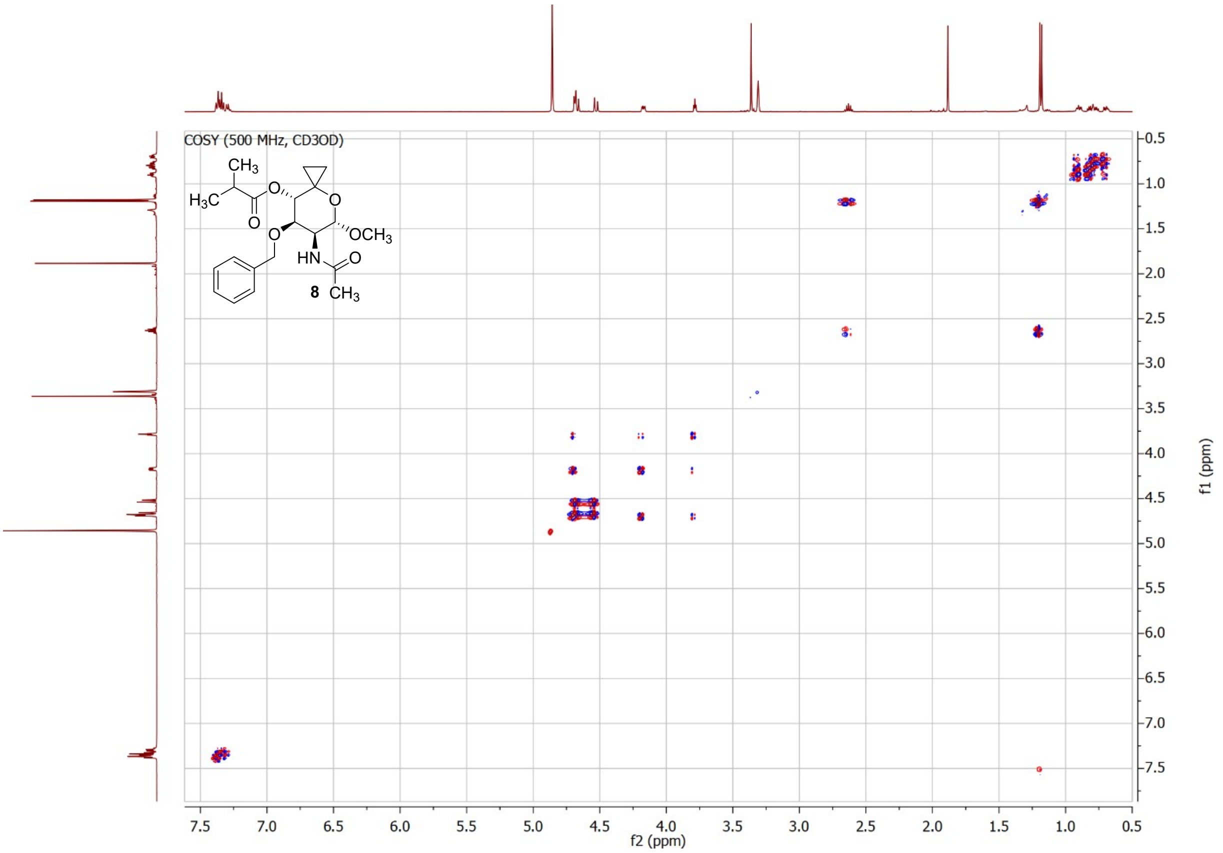

*Figure S50: COSY spectrum of compound **8**.*

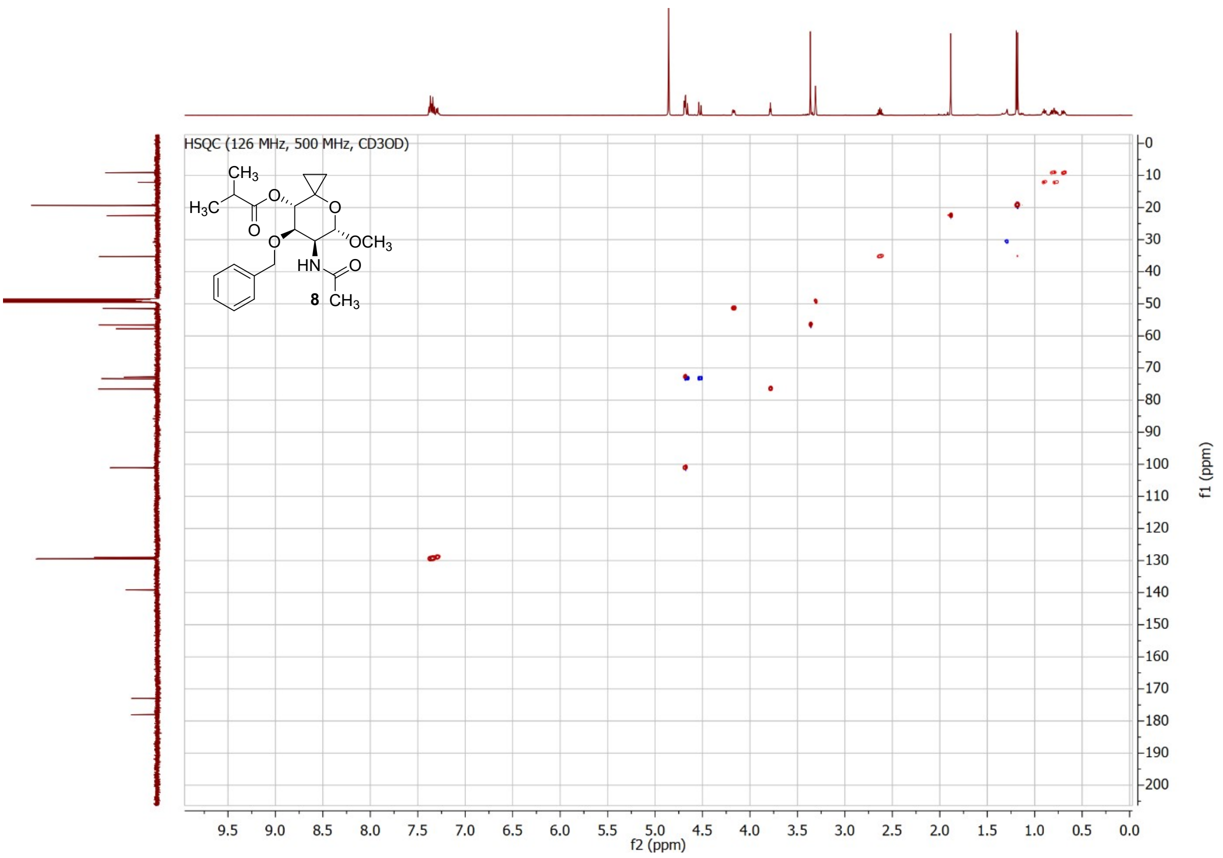

*Figure S51: HSQC spectrum of compound **8**.*

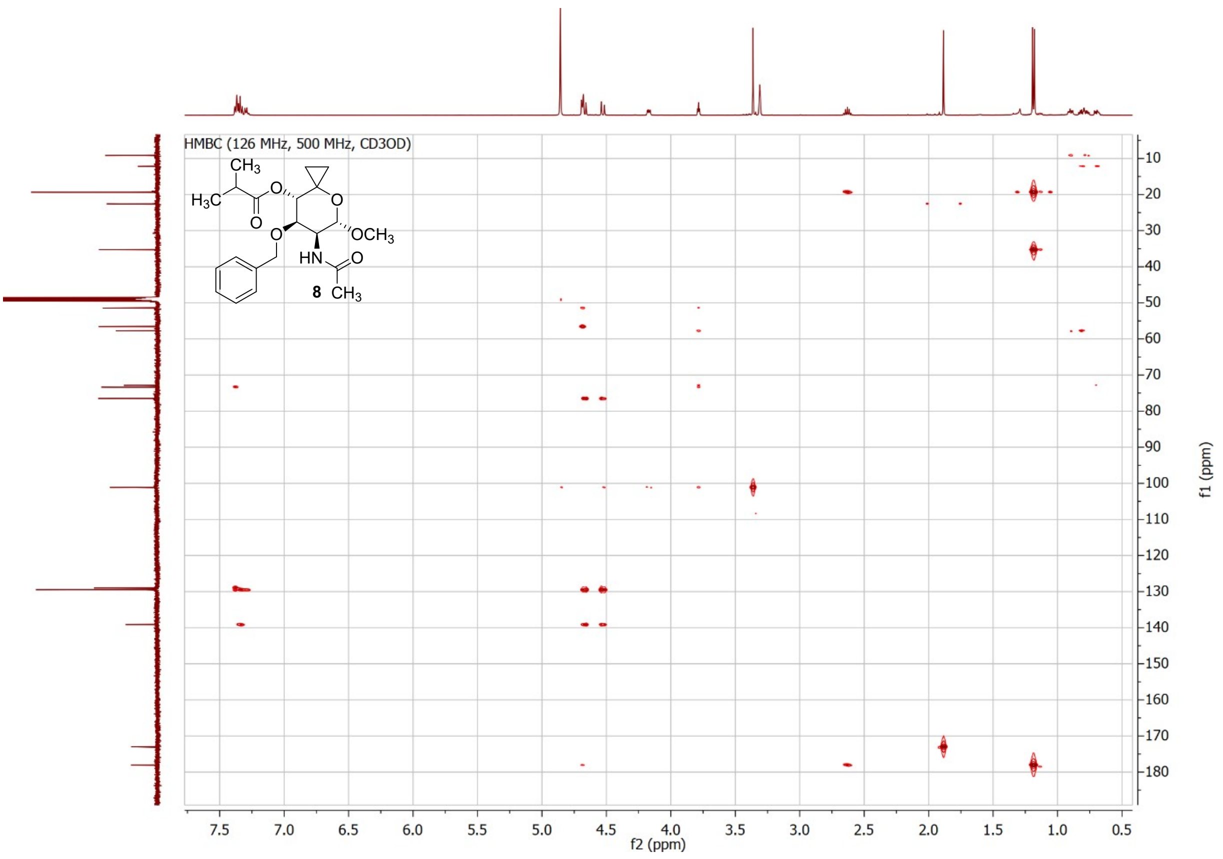

*Figure S52: HMBC spectrum of compound **8**.*

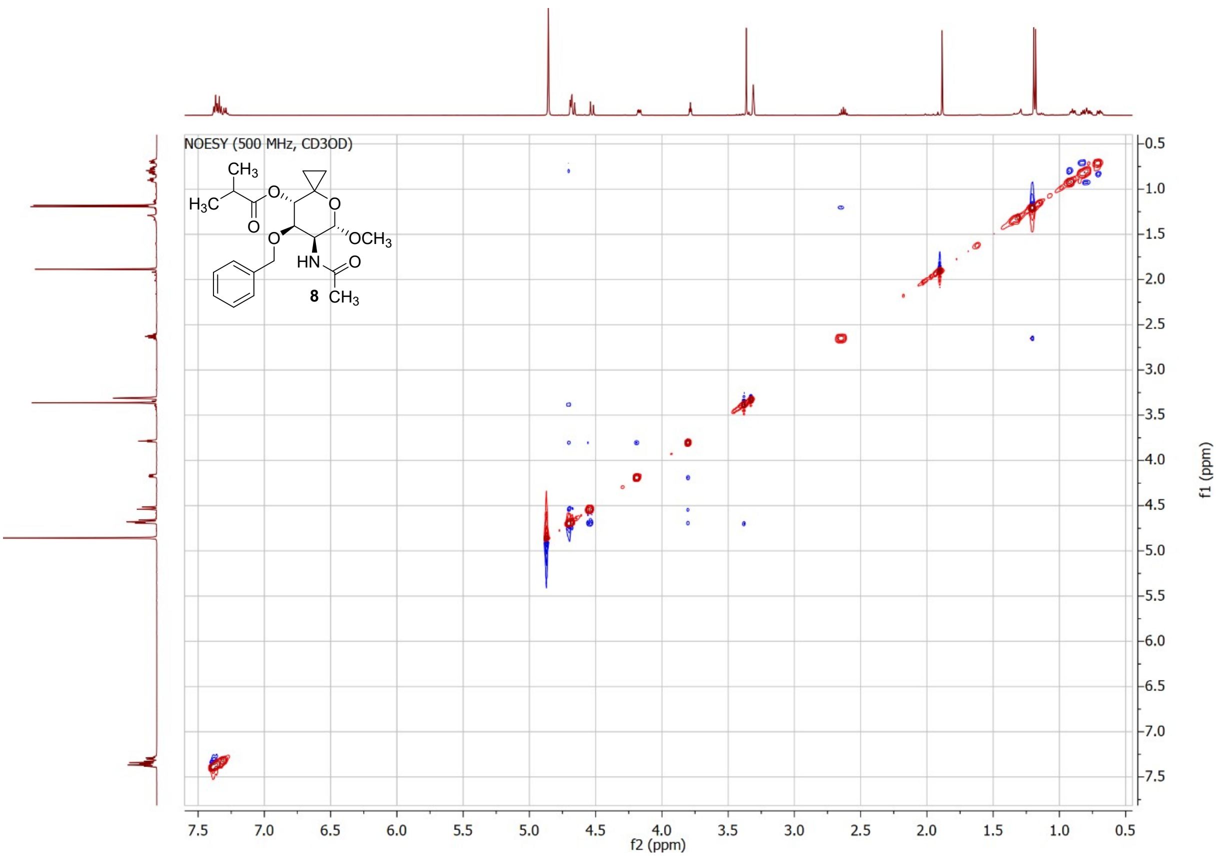

*Figure S53: NOESY spectrum of compound **8**.*

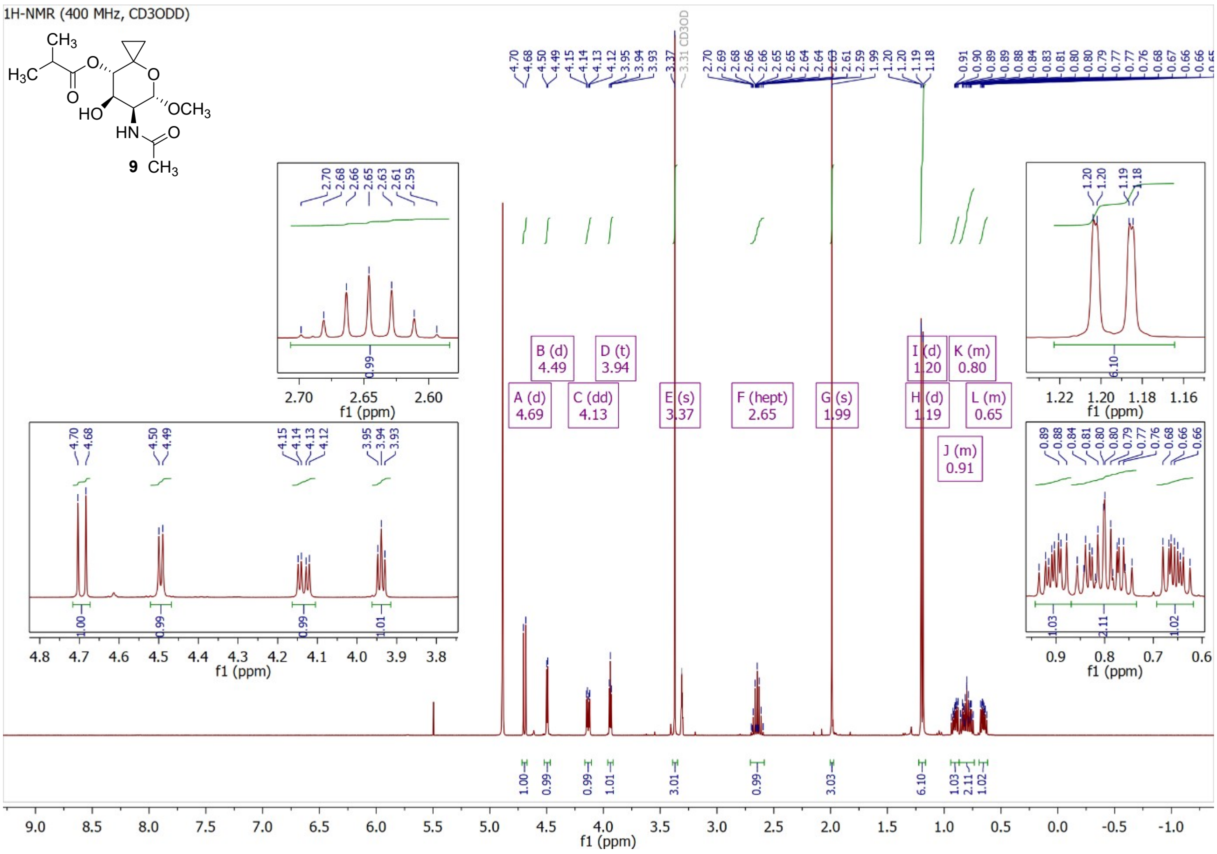

*Figure S54: ^{1}H-NMR spectrum of compound **9**.*

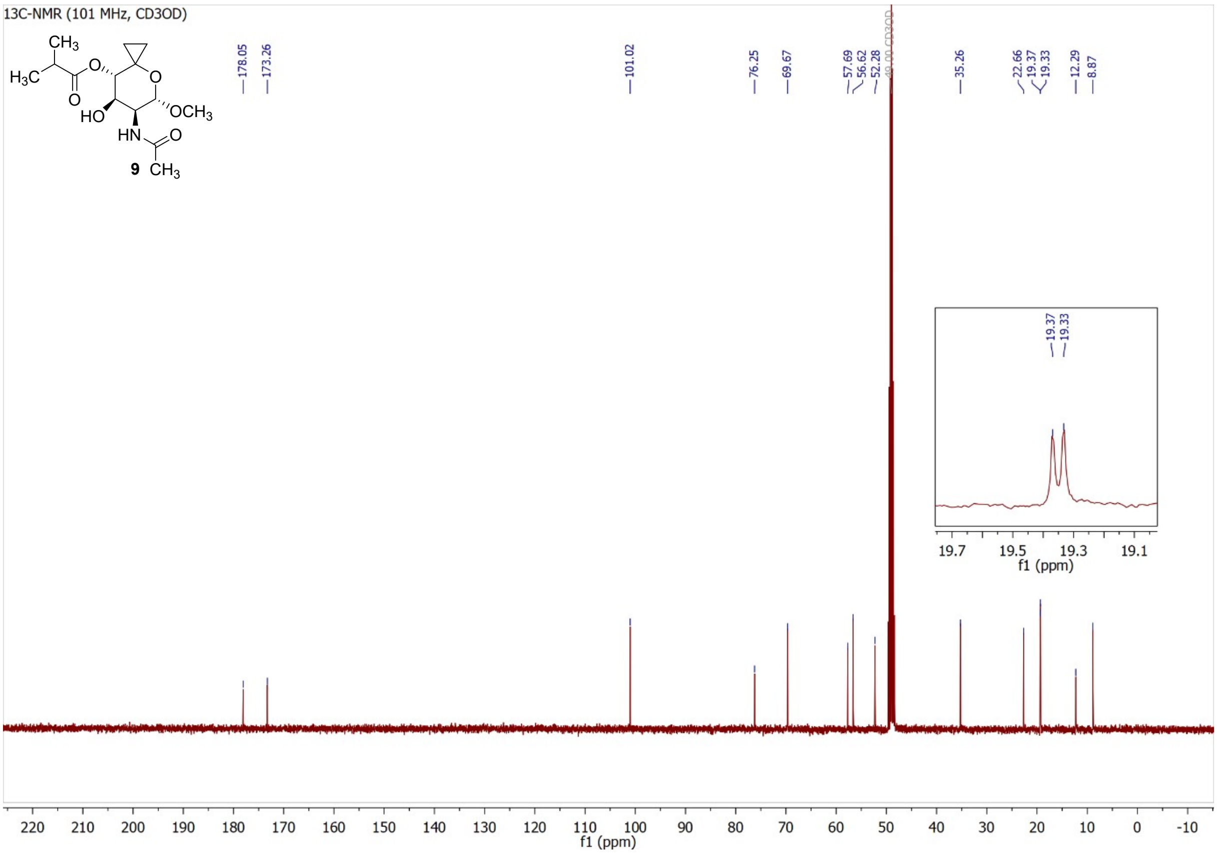

Figure S55: $^{13}C\{^{1}H\}$-NMR spectrum of compound **9**.

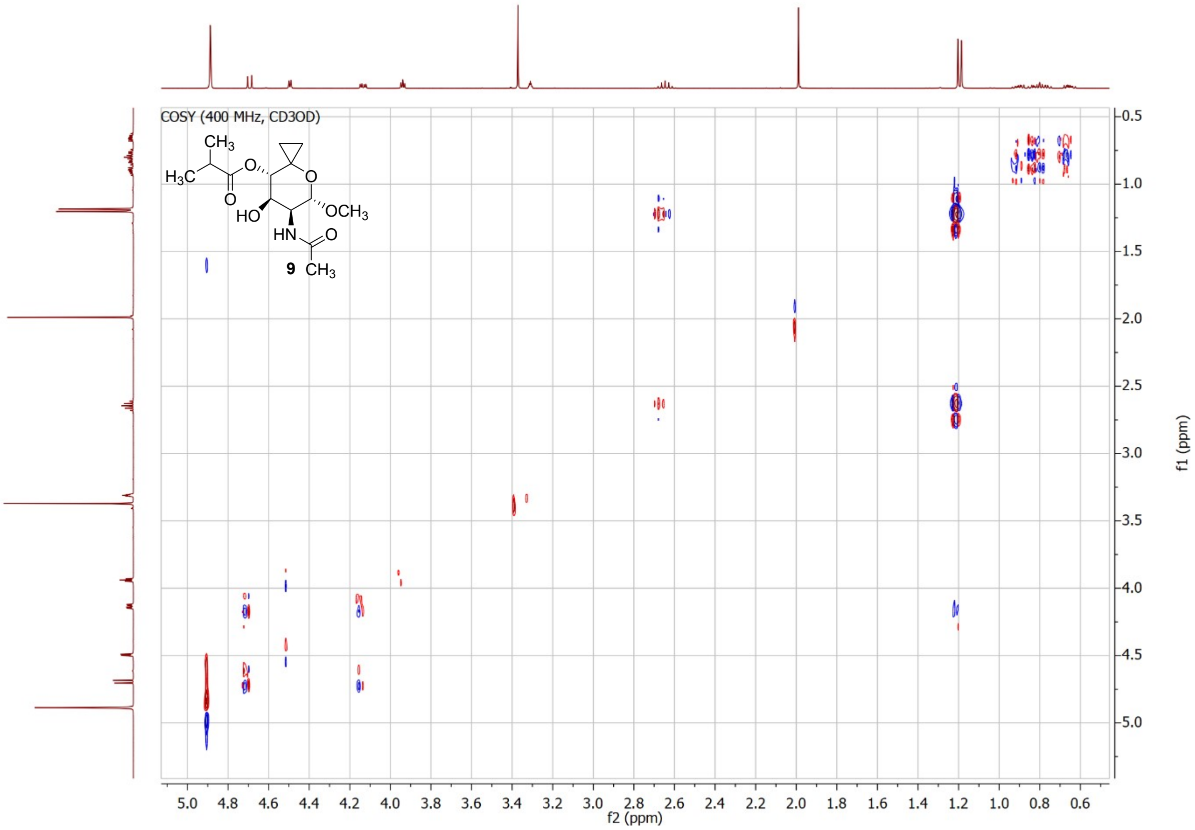

Figure S56: COSY spectrum of compound **9**.

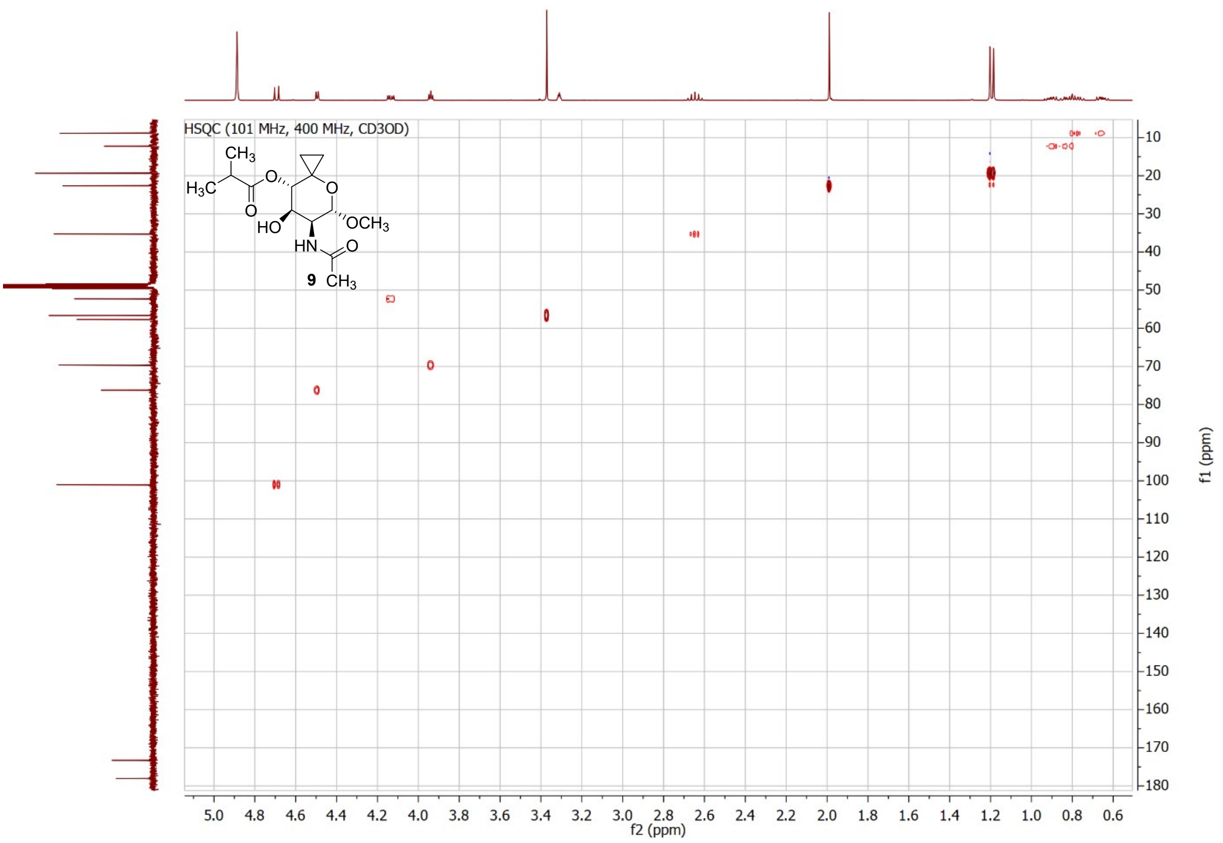

*Figure S57: HSQC spectrum of compound **9**.*

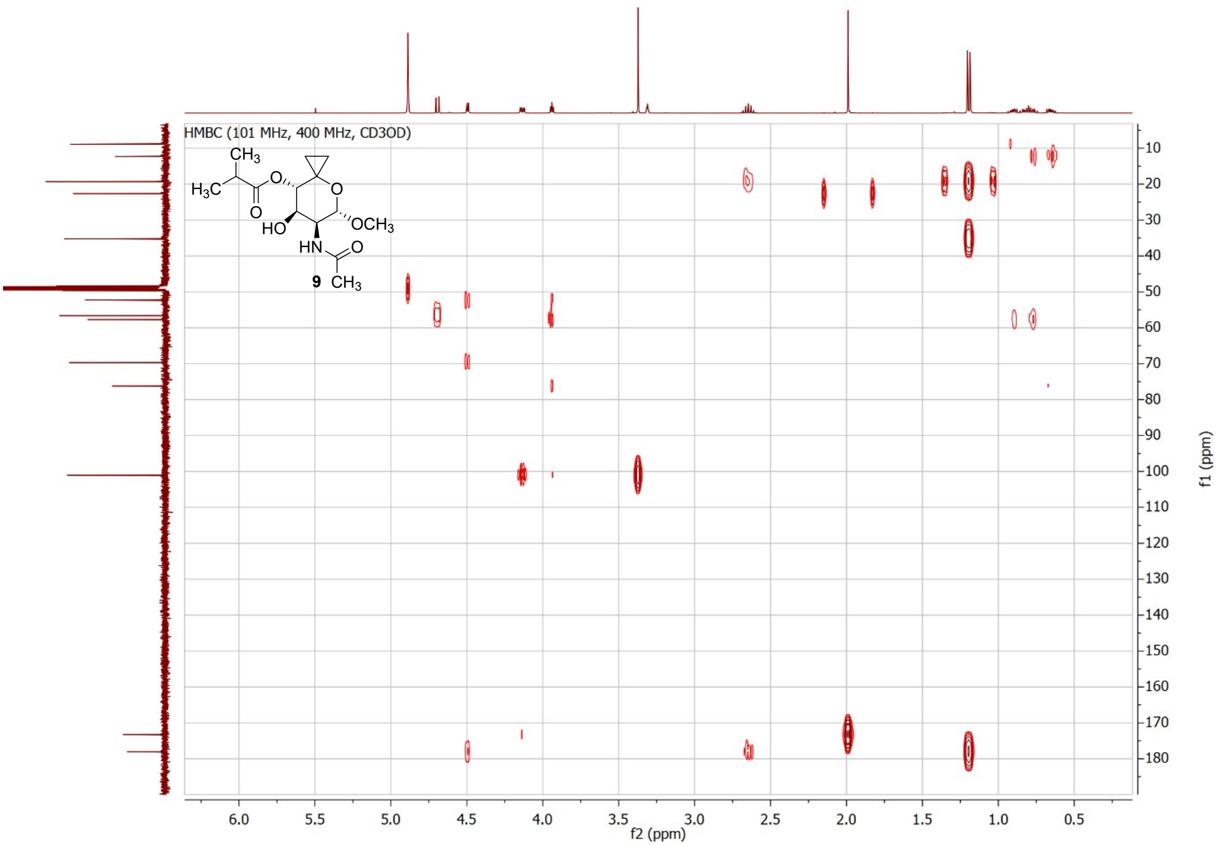

*Figure S58: HMBC spectrum of compound **9**.*

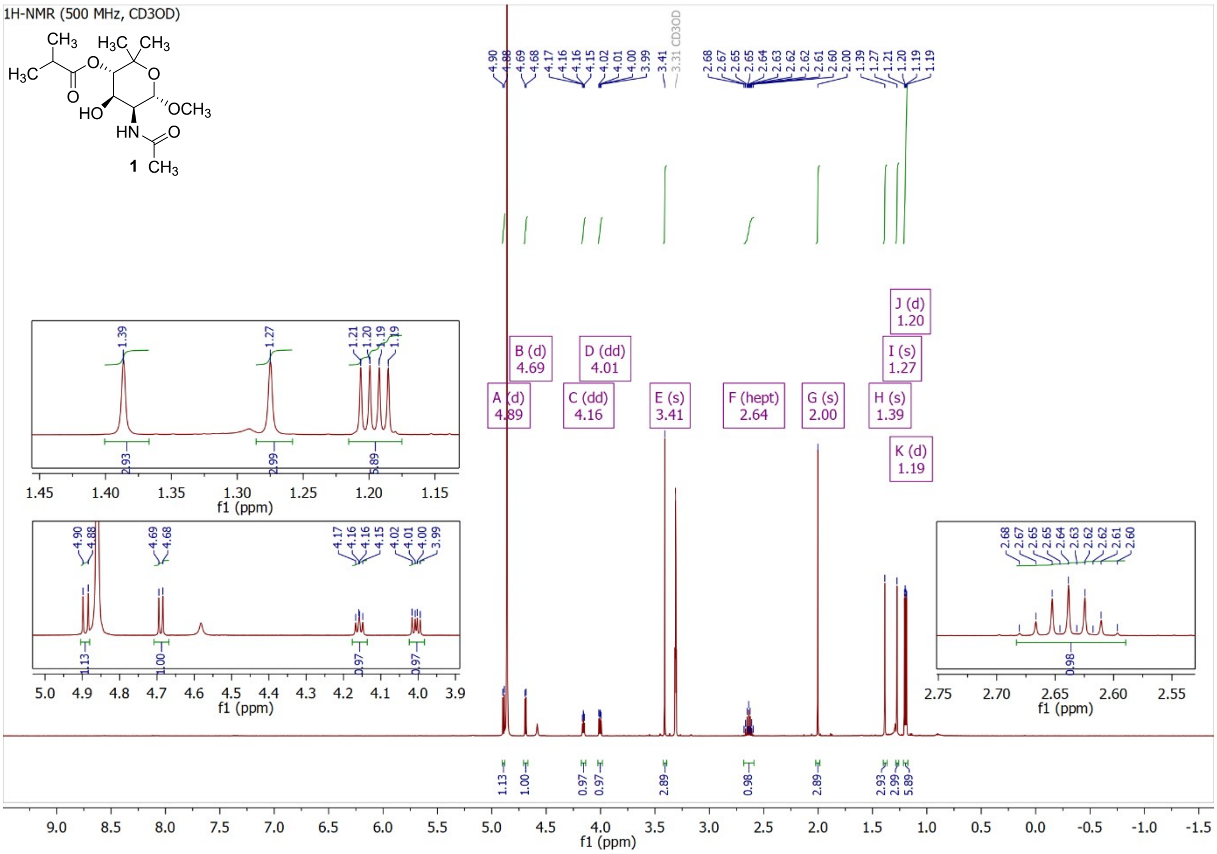

*Figure S59: ^{1}H-NMR spectrum of 2-acetylnoviosamine **1**.*

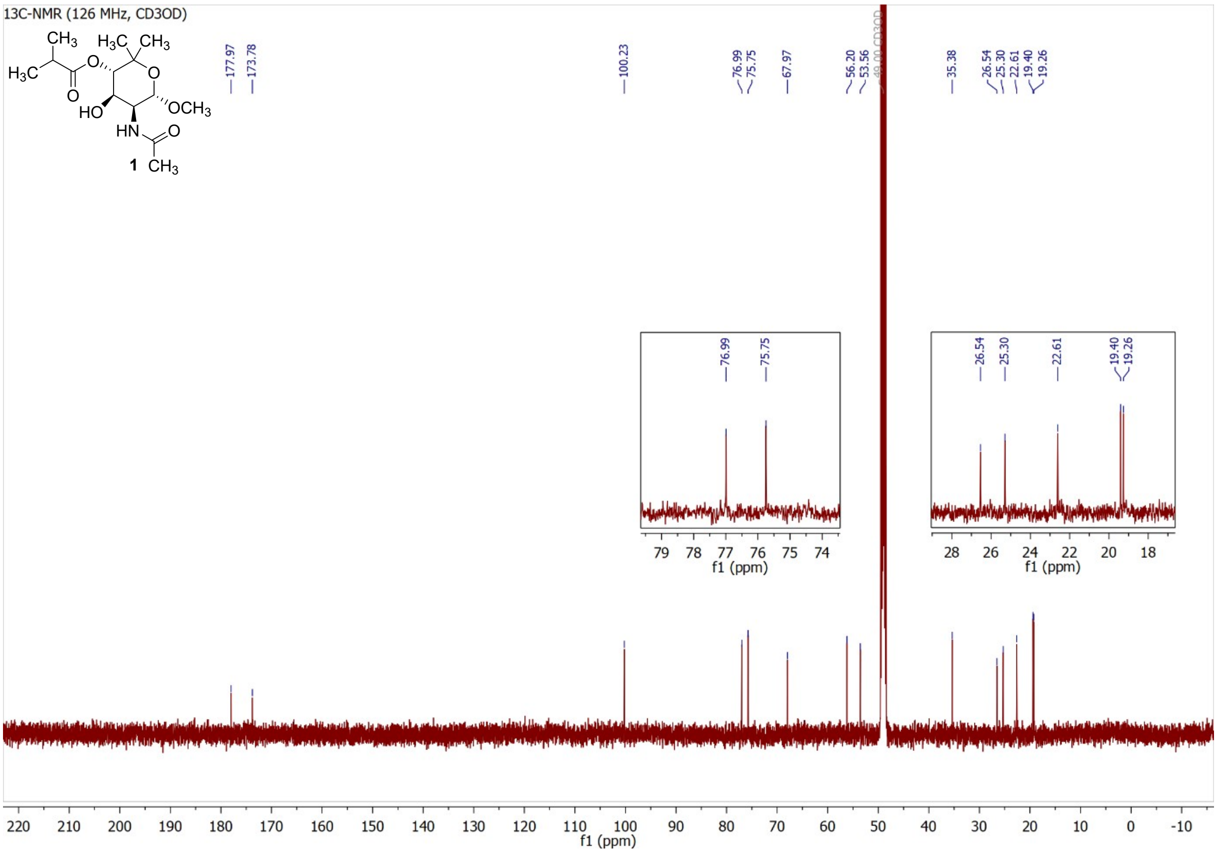

*Figure S60: $^{13}C\{^{1}H\}$-NMR spectrum of 2-acetylnoviosamine **1**.*

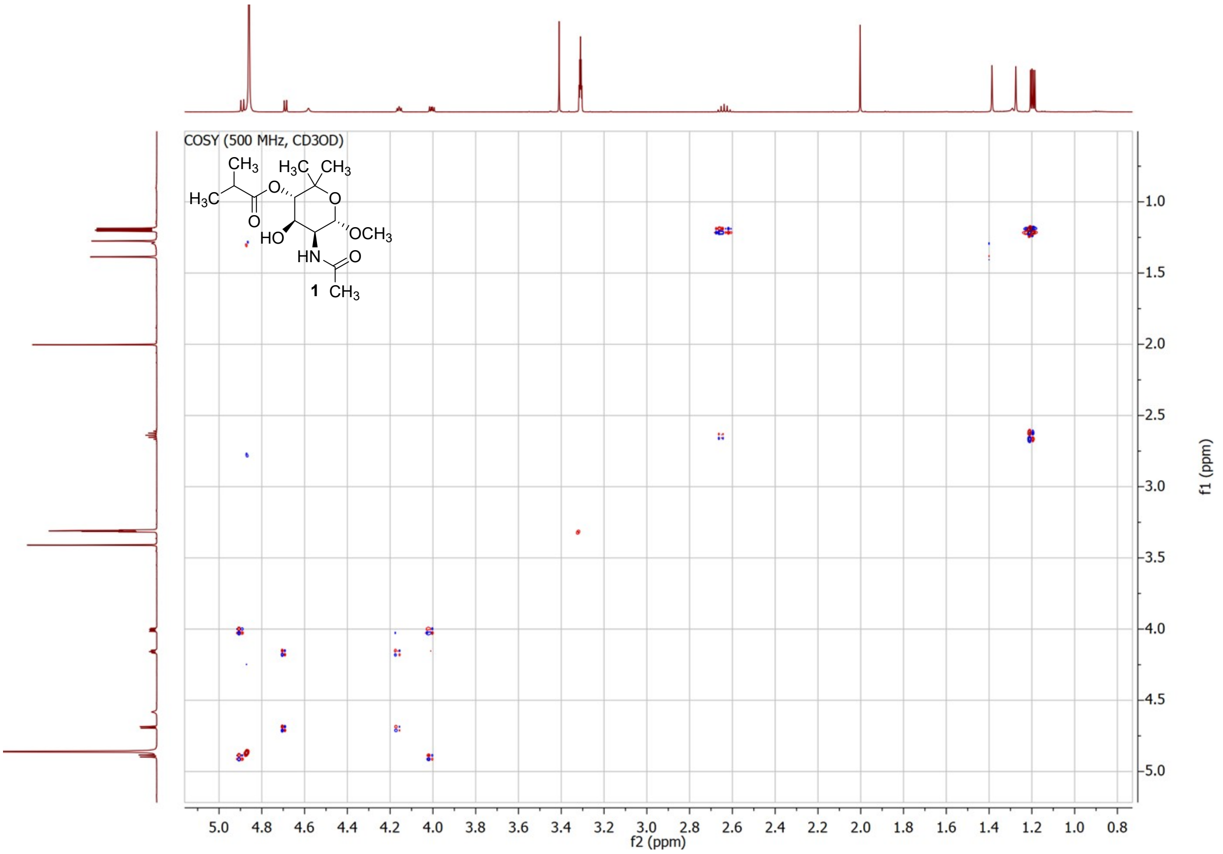

Figure S61: COSY spectrum of 2-acetylnoviosamine ***1****.*

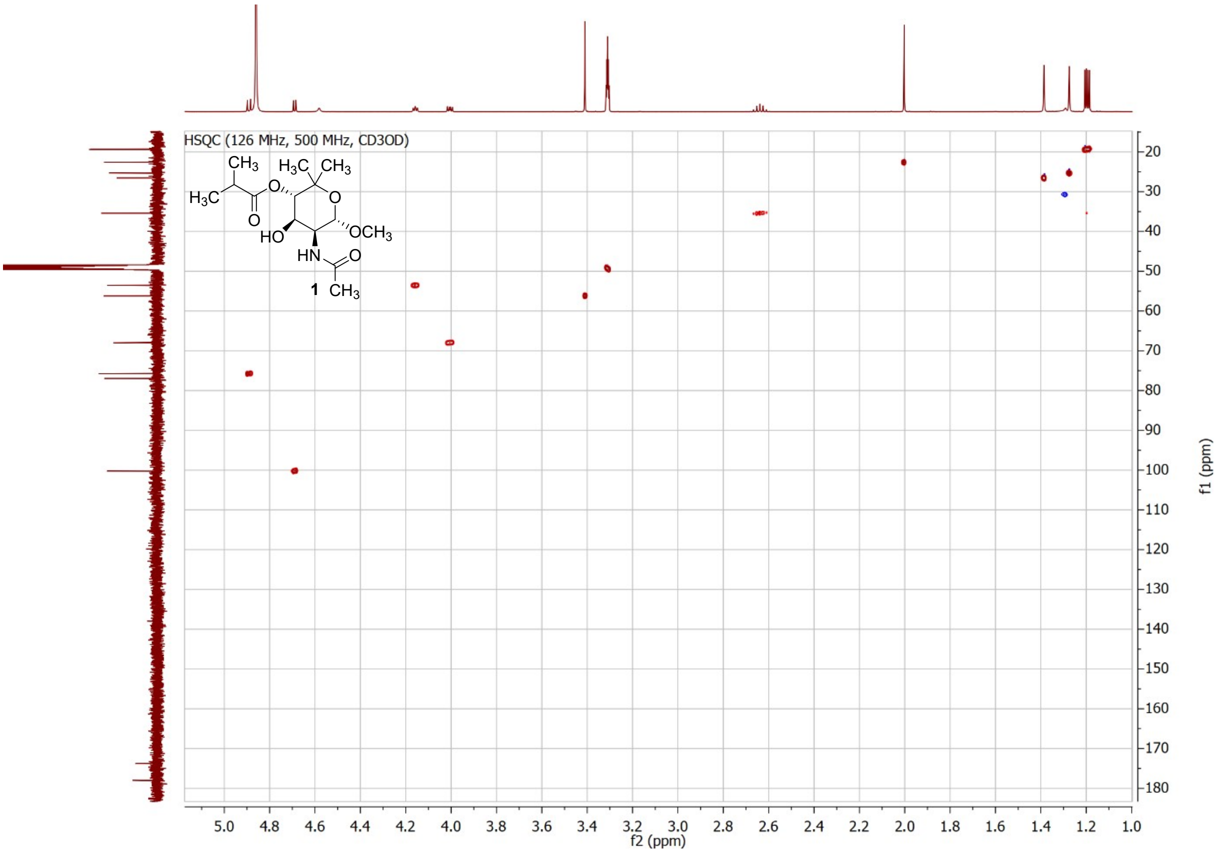

Figure S62: HSQC spectrum of 2-acetylnoviosamine ***1****.*

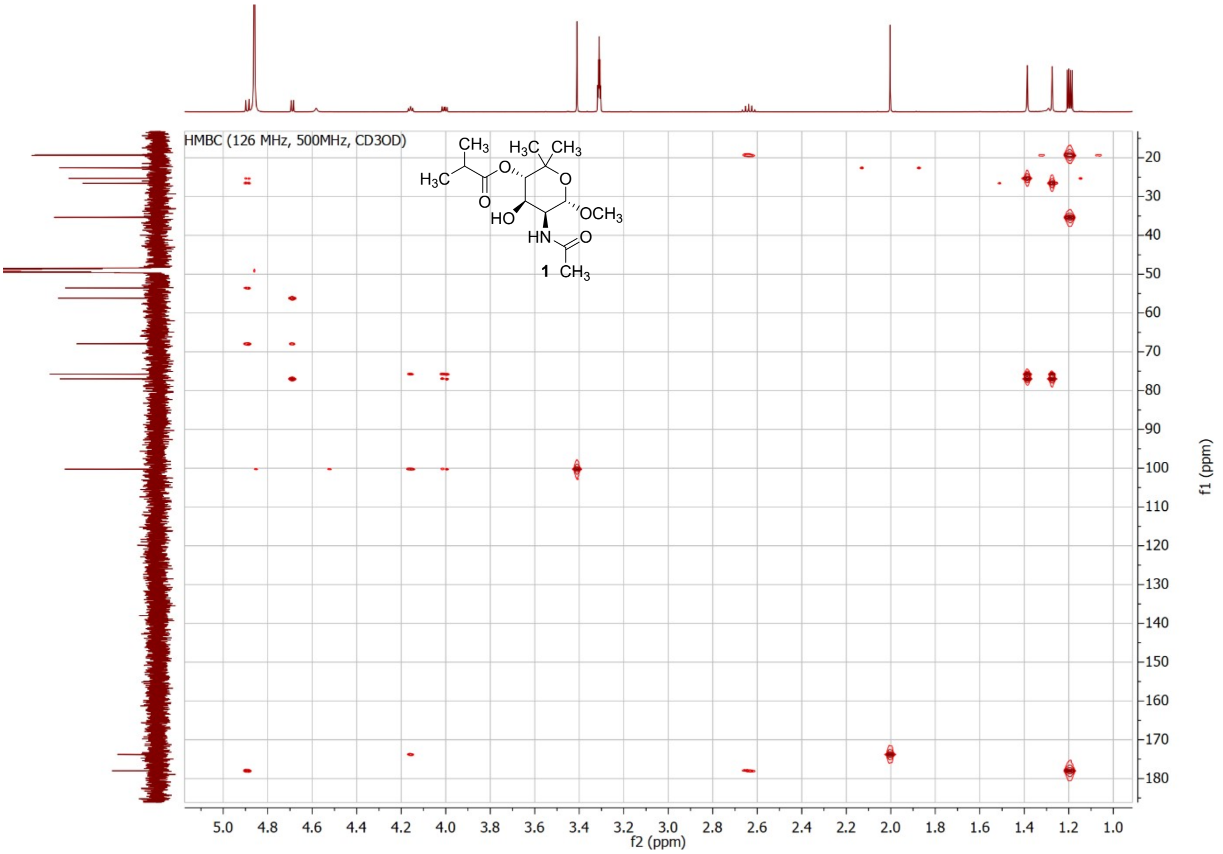

*Figure S63: HMBC spectrum of 2-acetylnoviosamine **1**.*

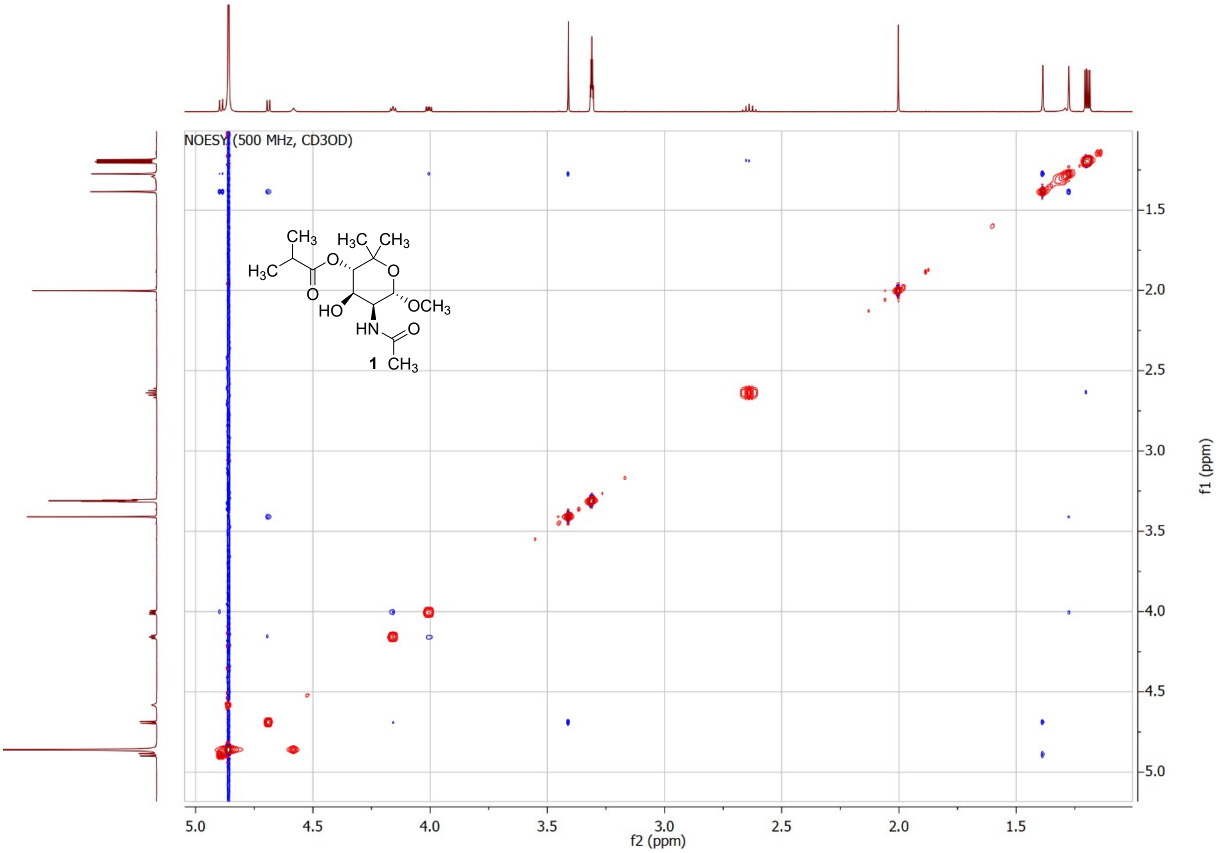

*Figure S64: NOESY spectrum of 2-acetylnoviosamine**1**.*

IX. Crystallographic data XRD

*Table S3: Crystallographic data for olefin **2** (CCDC 2430253) and spirocyclopropane **8** (CCDC 2430254).*

Oak Ridge thermal-ellipsoid plot (p = 0.5)	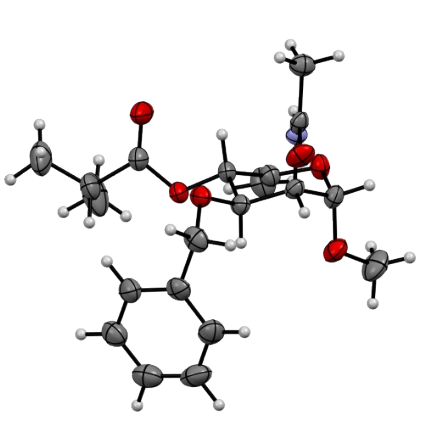	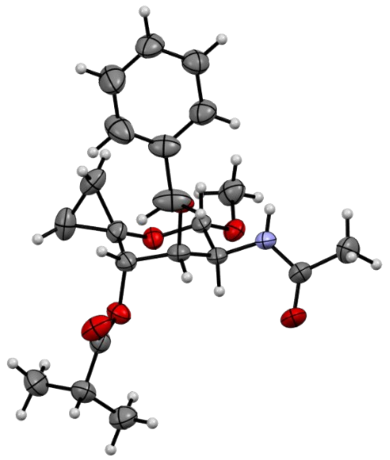
Compound number	**2**	**8**
Empirical formula	$C_{20}H_{27}NO_6$	$C_{21}H_{29}NO_6$
Formula weight	377.42	391.45
Temperature [K]	160.0(1)	160.0(1)
Crystal system	orthorhombic	orthorhombic
Space group	$P2_12_12_1$	$P2_12_12_1$
Unit cell parameters:		
a [Å]	9.5296(2)	14.7354(2)
b [Å]	9.5745(2)	15.1012(2)
c [Å]	45.0172(7)	29.1138(3)
α [°]	90	90
β [°]	90	90
γ [°]	90	90
Volume [$Å^3$]	4107.42(14)	6478.47(14)
Z	8	12
ρ_{calc} [g/cm^3]	1.221	1.204
μ [mm^{-1}]	0.743	0.723
F(000)	1616.0	2520.0
Crystal size [mm^3]	0.14 × 0.07 × 0.06	0.23 × 0.13 × 0.1
Radiation	Cu Kα (λ = 1.54184)	Cu Kα (λ = 1.54184)
2Θ range for data collection [°]	3.926 to 148.98	6.072 to 153.45
Index ranges	-11 ≤ h ≤ 11, -11 ≤ k ≤ 11, -56 ≤ l ≤ 49	-18 ≤ h ≤ 17, -18 ≤ k ≤ 18, -36 ≤ l ≤ 36
Reflections collected	83504	69614
Independent reflections	8376 [R_{int} = 0.0591, R_{sigma} = 0.0261]	13538 [R_{int} = 0.0690, R_{sigma} = 0.0356]
Data/restraints/parameters	8376/286/550	13538/172/831
Goodness-of-fit on F^2	1.030	1.040
Final R indexes [I>=2σ (I)]	R_1 = 0.0383, wR_2 = 0.0968	R_1 = 0.0480, wR_2 = 0.1262
Final R indexes [all data]	R_1 = 0.0445, wR_2 = 0.1022	R_1 = 0.0522, wR_2 = 0.1319
Largest diff. peak/hole / e $Å^{-3}$	0.21/-0.27	0.45/-0.22
Flack parameter	0.00(7)	-0.04(5)

X. References SI

(1) Kumar, A.; Doddi, V. R.; Vankar, Y. D. Mild and Efficient Chemoselective Deprotection of Anomeric O-Methyl Glycosides with Trityl Tetrafluoroborate. *J. Org. Chem.* **2008**, *73* (15), 5993–5995. https://doi.org/10.1021/jo800693w.

(2) Clark, R. C.; Reid, J. S. The Analytical Calculation of Absorption in Multifaceted Crystals. *Acta Crystallogr. Sect. A* **1995**, *51* (6), 887–897. https://doi.org/10.1107/S0108767395007367.

(3) Dolomanov, O. V.; Bourhis, L. J.; Gildea, R. J.; Howard, J. a. K.; Puschmann, H. OLEX2: A Complete Structure Solution, Refinement and Analysis Program. *J. Appl. Crystallogr.* **2009**, *42* (2), 339–341. https://doi.org/10.1107/S0021889808042726.

(4) Sheldrick, G. M. SHELXT – Integrated Space-Group and Crystal-Structure Determination. *Acta Crystallogr. Sect. Found. Adv.* **2015**, *71* (1), 3–8. https://doi.org/10.1107/S2053273314026370.

(5) Sheldrick, G. M. Crystal Structure Refinement with SHELXL. *Acta Crystallogr. Sect. C Struct. Chem.* **2015**, *71* (1), 3–8. https://doi.org/10.1107/S2053229614024218.

(6) Spek, A. L. Structure Validation in Chemical Crystallography. *Acta Crystallogr. D Biol. Crystallogr.* **2009**, *65* (2), 148–155. https://doi.org/10.1107/S090744490804362X.

(7) Petrović, V.; Car, Ž.; Prugovečki, B.; Tomić, S.; Matković-Čalogović, D. Synthesis of Acylated Methyl 2-Acetamido-2-Deoxy-α-D-Mannopyranosides. *J. Carbohydr. Chem.* **2006**, *25* (8–9), 685–695. https://doi.org/10.1080/07328300601039351.

(8) Alex, C.; Visansirikul, S.; Zhang, Y.; Yasomanee, J. P.; Codee, J.; Demchenko, A. V. Synthesis of 2-Azido-2-Deoxy- and 2-Acetamido-2-Deoxy-D-Manno Derivatives as Versatile Building Blocks. *Carbohydr. Res.* **2020**, *488*, 107900. https://doi.org/10.1016/j.carres.2019.107900.

(9) Basu, N.; Maity, S. K.; Roy, S.; Singha, S.; Ghosh, R. FeCl3 Mediated Arylidenation of Carbohydrates. *Carbohydr. Res.* **2011**, *346* (5), 534–539. https://doi.org/10.1016/j.carres.2011.01.003.

(10) Petrakova, E.; Spohr, U.; Lemieux, R. U. Molecular Recognition IX. The Synthesis of the H-Type 2 Human Blood Group Determinant and Congeners Modified at the 6-Position of the *N* - Acetylglucosamine Unit. *Can. J. Chem.* **1992**, *70* (1), 233–240. https://doi.org/10.1139/v92-034.

(11) Scaffidi, A.; Stubbs, K. A.; Dennis, R. J.; Taylor, E. J.; Davies, G. J.; Vocadlo, D. J.; Stick, R. V. A 1-Acetamido Derivative of 6-Epi-Valienamine: An Inhibitor of a Diverse Group of β-N-Acetylglucosaminidases. *Org. Biomol. Chem.* **2007**, *5* (18), 3013–3019. https://doi.org/10.1039/B709681J.

(12) Skaanderup, P. R.; Poulsen, C. S.; Hyldtoft, L.; Jørgensen, M. R.; Madsen, R. Regioselective Conversion of Primary Alcohols into Iodides in Unprotected Methyl Furanosides and Pyranosides. *Synthesis* **2002**, *2002* (12), 1721–1727. https://doi.org/10.1055/s-2002-33641.

(13) Sieber, S.; Carlier, A.; Neuburger, M.; Grabenweger, G.; Eberl, L.; Gademann, K. Isolation and Total Synthesis of Kirkamide, an Aminocyclitol from an Obligate Leaf Nodule Symbiont. *Angew. Chem. Int. Ed.* **2015**, *54* (27), 7968–7970. https://doi.org/10.1002/anie.201502696.

(14) Hedberg, C.; Estrup, M.; Eikeland, E. Z.; Jensen, H. H. Vinyl Grignard-Mediated Stereoselective Carbocyclization of Lactone Acetals. *J. Org. Chem.* **2018**, *83* (4), 2154–2165. https://doi.org/10.1021/acs.joc.7b03079.

(15) Lorenz, J. C.; Long, J.; Yang, Z.; Xue, S.; Xie, Y.; Shi, Y. A Novel Class of Tunable Zinc Reagents (RXZnCH2Y) for Efficient Cyclopropanation of Olefins. *J. Org. Chem.* **2004**, *69* (2), 327–334. https://doi.org/10.1021/jo030312v.

(16) Binkley, R. W.; Hehemann, D. G. A Light-Initiated Process for Rapid Debenzylation of Carbohydrates. *J. Org. Chem.* **1990**, *55* (1), 378–380. https://doi.org/10.1021/jo00288a074.
